# Return of the founder Chikungunya virus to its place of introduction into Brazil is revealed by genomic characterization of exanthematic disease cases

**DOI:** 10.1080/22221751.2019.1701954

**Published:** 2019-12-27

**Authors:** Zuinara Pereira Gusmão Maia, Felicidade Mota Pereira, Rodrigo Fabiano do Carmo Said, Vagner Fonseca, Tiago Gräf, Fernanda de Bruycker Nogueira, Vanessa Brandão Nardy, Joilson Xavier, Maricelia Lima Maia, André L. Abreu, Carlos F. Campelo de Albuquerque, Wanderson Kleber Oliveira, Julio Croda, Ana Maria Bispo de Filippis, Rivaldo Venancio Cunha, Jose Lourenço, Tulio de Oliveira, Nuno Rodrigues Faria, Luiz Carlos Junior Alcantara, Marta Giovanetti

**Affiliations:** a Laboratório Central de Saúde Pública, Departamento de Virologia, Salvador, BH, Brazil; b Coordenação Geral de Vigilância de Arboviroses (CGARB); c Laboratório de Genética Celular e Molecular, Instituto de Ciências Biológicas, Universidade Federal de Minas Gerais, Belo Horizonte, Brazil; d KwaZulu-Natal Research Innovation and Sequencing Platform (KRISP), College of Health Sciences, University of KwaZulu-Natal, Durban, South Africa; e Laboratorio de Patologia Experimental, Instituto Gonçalo Moniz, Fiocruz, Salvador, BH, Brazil; f Laboratório de Flavivírus, Instituto Oswaldo Cruz, Fundação Oswaldo Cruz, Rio de Janeiro, RJ, Brazil; g Universidade Estadual de Feira de Santana, Feira de Santana, Brazil; h Secretaria de Saúde de Feira de Santana, Feira de Santana, Brazil; i Coordenação Geral dos Laboratórios de Saúde Pública/Secretaria de Vigilância em Saúde, Ministério da Saúde, (CGLAB/SVS-MS) Brasília, Distrito Federal, Brazil; j Organização Pan-Americana da Saúde/Organização Mundial da Saúde, Brasília, Distrito Federal, Brazil; k Secretaria de Vigilância em Saúde, Ministério da Saúde (SVS-MS), Brasília, Distrito Federal, Brazil; l Departamento de Vigilância de Doenças Transmissíveis/Secretaria de Vigilância em Saúde, Ministério da Saúde (DEVIT/SVS-MS), Brasilia, Brazil; m Federal University of Mato Grosso do Sul, Brazil; n Fundação Oswaldo Cruz, Coordination of Health Surveillance and Reference Laboratories, Rio de Janeiro, RJ, Brazil; o Department of Zoology, University of Oxford, Oxford, UK

**Keywords:** Chikungunya, East-Central-South-African genotype, Northeast Brazil, surveillance, portable genome sequencing

## Abstract

Between June 2017 and August 2018, several municipalities located in Bahia state (Brazil) reported a large increase in the number of patients presenting with febrile illness similar to that of arboviral infections. Using a combination of portable whole genome sequencing, molecular clock and epidemiological analyses, we revealed the return of the CHIKV-ECSA genotype into Bahia. Our results show local persistence of lineages in some municipalities and the re-introduction of new epidemiological strains from different Brazilian regions, highlighting a complex dynamic of transmission between epidemic seasons and sampled locations. Estimated climate-driven transmission potential of CHIKV remained at similar levels throughout the years, such that large reductions in the total number of confirmed cases suggests a slow, but gradual accumulation of herd-immunity over the 4 years of the epidemic in Bahia after its introduction in 2014. Bahia remains a reservoir of the genetic diversity of CHIKV in the Americas, and genomic surveillance strategies are essential to assist in monitoring and understanding arboviral transmission and persistence both locally and over large distances.

## Text

Chikungunya virus (CHIKV) is an emerging mosquito-borne virus belonging to the genus *Alphavirus* and *Togaviridae* family and poses a significant public health problem in tropical and subtropical regions [1]. CHIKV infection results in an acute, debilitating febrile illness, but may also become a chronic condition, with persistent or relapse symptoms of arthropathy [1]. Virus diversity can be classified into four distinct virus genotypes**:** the Asian, the West African, the East-Central-South-African (ECSA) and the Indian Ocean Lineage (IOL) [1]. In September 2014, the first autochthonous CHIKV infections were confirmed in Brazil. They were characterized by two distinct genotypes: the CHIKV Asian genotype was found in Oiapoque, a city in the North of Brazil, close to the Caribbean; and CHIKV-ECSA, which originated from an infected individual in Angola and was found in Feira de Santana, Bahia in Northeast Brazil [1]. Since then, CHIKV-ECSA has been detected in several other Brazilian states in the Northeast, Southeastern and Northern regions, exerting significant pressures on local public health systems [2–4].

**Figure 1.**
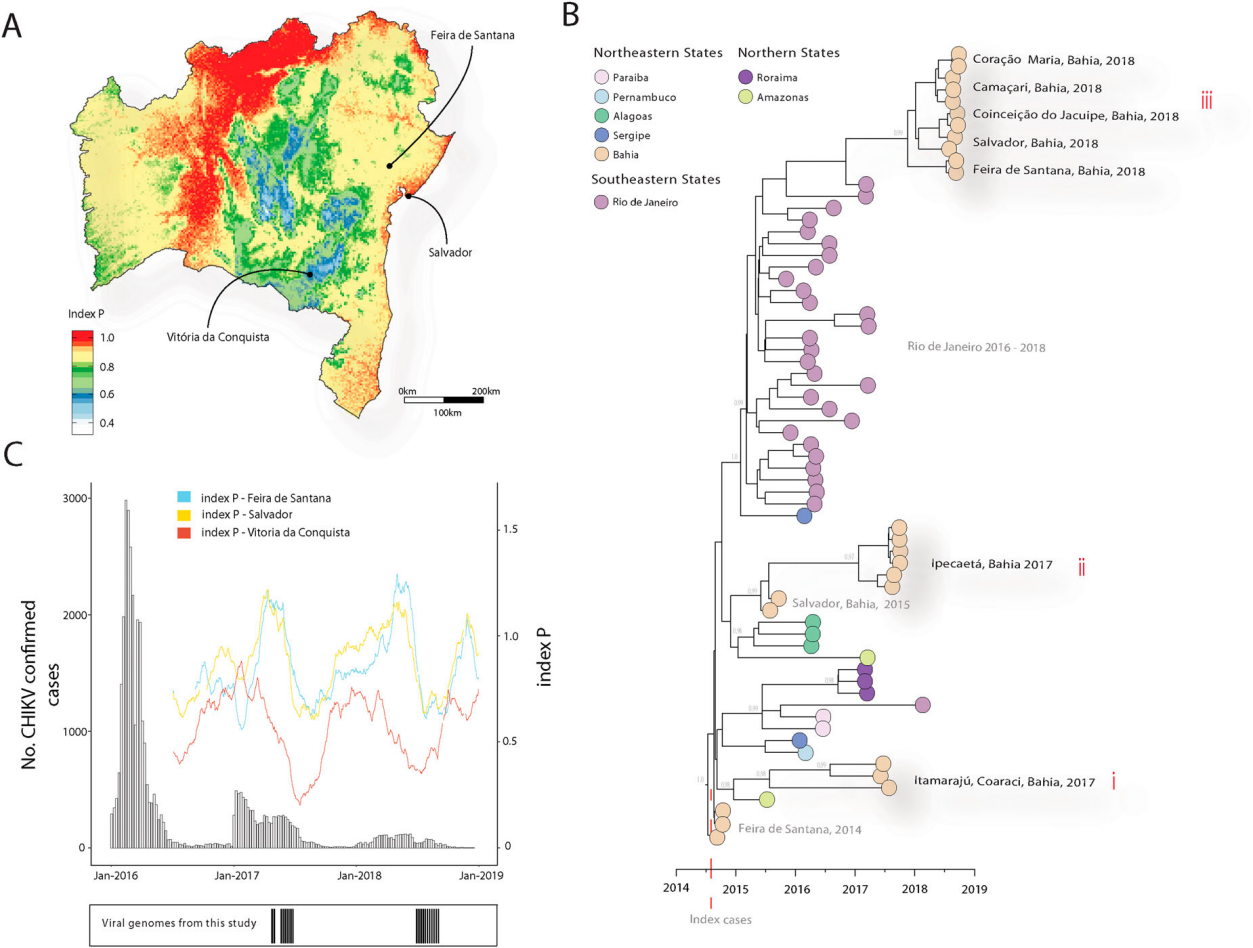
Return of the CHIKV-ECSA genotype into Bahia state northeast Brazil. (A) Spatial estimation of index P across the Bahia state, with Salvador, Feira de Santana and Vitoria da Conquista identified. Each pixel is the yearly mean index P (≈ 20 Km2) estimated using WorldClim V2 dataset as in [5]. Values coloured according to scale on the left. (B) Molecular clock phylogeny obtained using 20 new CHIKV near-complete genome sequences from the 2017–2018 epidemic in the municipality of Feira de Santana (black tips). Salvador, Camaçari, Coração de Maria, Conceição do Jacuipe, Coaraci, Itamarajú e Ipecaetá, in the Bahia State, Northeastern Brazil plus 47 publicly available Brazilian CHIKV-ECSA lineage sequences. Numbers along branches represent clade posterior probability >0.90. Colours represent different locations. (C) Weekly number of laboratory confirmed cases of CHIKV between 2016 and 2018, superimposed with daily mean index P and 95% confidence interval (second Y axis). At the bottom, black bars indicate the collection dates of the viral genome sequences (*n* = 20) generated in this study.

Between June 2017 and August 2018, Bahia (Figure 1 panel A) reported several cases (across municipalities) of patients presenting febrile illness, compatible with arboviral infection, characterized by severe joint pain accompanied by edema, rash, skin and ocular manifestations, plantar fasciitis and morning joint stiffness. Using a combination of portable genome sequencing and genomic epidemiology in this study, we revealed the re-introduction of ECSA genotype into Bahia.

Serum samples obtained from 24 patients presenting symptoms compatible with arboviral infection were collected for molecular diagnostics and sent for testing at the Central Laboratory of Public Health (LACEN) in Salvador, Bahia. These samples were from several municipalities in Bahia and represented the main outbreak period of 2017 and 2018 (Figure 1C). Viral RNA was extracted from clinical samples using the QIAmp Viral RNA Mini kit (Qiagen) and submitted to real time RT-qPCR protocols for the detection of Zika virus (ZIKV), dengue virus (DENV 1-4), mayaro virus (MAYV), oropouche virus (OROV) and CHIKV as previously described [6–9]. Samples were selected for sequencing based on Ct-value <33 (to maximize genome coverage from clinical samples by nanopore sequencing [10]). Extracted RNA was converted to cDNA [10] using ProtoScript II First Strand cDNA Synthesis Kit. Then, a multiplex tiling PCR was conducted using Q5 High Fidelity Hot-Start DNA Polymerase (New England Biolabs) and the CHIKV primers scheme, as previously described [10]. DNA library preparation was performed using the Ligation Sequencing Kit (Oxford Nanopore Technologies, ONT) and the Native Barcoding Kit (NBD103, ONT). A sequencing library was generated from the barcoded products using the Genomic DNA Sequencing Kit SQK-MAP007/SQK-LSK109 (ONT) then loaded onto a R9.4 flow cell (ONT).

Genotyping was conducted using the phylogenetic arbovirus subtyping tool available at http://genomedetective.com/app/typingtool/chikungunya [11] and then confirmed using Maximum Likelihood (ML) phylogeny. To investigate the dynamics of the CHIKV infection at a larger spatial scale, we generated a dataset that included all CHIKV-ECSA sequences from Brazil (ECSA-BR dataset) (*n* = 67). The ML tree was estimated using IQ-TREE 1.6.8 software [12] under the HKY nucleotide substitution model with four gamma categories (HKY+G4), which was inferred in jModelTest as the best fitting model [3]. The statistical robustness of tree topology was inspected using 100 bootstrap replicates. The ML phylogeny was used as a starting tree for Bayesian time-scaled phylogenetic analysis using BEAST 1.10.4 [13]. Data of weekly confirmed CHIKV cases in Bahia were supplied by the Brazilian Ministry of Health and were plotted using the R software version 3.5.1 (The R Foundation).

Diagnostic assays showed that, of the viruses tested, all the samples were positive only for CHIKV infection. From serum sampled during the two outbreaks in 2017 and 2018, we selected 20 RT-qPCR+ samples geographically widespread across eight municipalities in Bahia with a cycle threshold (Ct) ≤30 (mean 23.08, range: 16.25–27.09). From these samples near-complete genomes were obtained (coverage range 63%–96%, mean = 73%) using a nanopore sequencing approach and the pipeline from Quick et al. for the generation of consensus sequences (Supplementary table 1) (9).

Manual and automated phylogenetic analysis revealed that all the generated genomes belong to CHIKV-ECSA genotype. ML and Bayesian phylogenetic analyses showed that since 2014, Bahia has experienced different events of CHIKV re-introduction and epidemic spread (Figure 1 panel B). The first re-introduction event dates back to July 2015 (95% Bayesian credible interval, BCI, January 2015–November 2016). Thus, samples from the municipalities of Itamarajú and Coaraci in South Bahia, are related to a basal strain from Feira de Santana, that was shown to be the founder ECSA virus from Angola [2] (clade i, Figure 1 panel B). The second re-introduction event, which dates back to January 2017 (95% BCI, October 2016–August 2017) suggests the persistence of a second lineage in the municipality of Ipecaetá (clade ii, Figure 1 panel B). Interestingly, a 2015 strain from the capital city of Bahia (Salvador, at 150Km from Ipecaetá) is associated with this re-introduction. Finally, a third re-introduction to several municipalities in the metropolitan regions of Salvador and Feira de Santana (clade iii, Figure 1 panel B) is estimated to date back to November 2018 (95% BCI, August 2017 to May 2018) with source in Rio de Janeiro (Southeastern Brazil); a city that experienced an explosive ECSA epidemic between 2016–2018 (5).

Using freely accessible climatic data from the US National Climate Data Center [14] we estimated mosquito-borne viral suitability using the index P (transmission potential [15]) for the three largest urban centres of Bahia; assuming *Aedes*-like priors as in Lourenco et al. 2019 and CHIKV-like priors with human incubation period of mean 3 days (standard deviation, STD 1) and human infectious period 4.5 days (STD 2). We estimated Higher potential in Salvador and Feira de Santana when compared with Vitoria da Conquista, with peaks in the latter mirroring the troughs in the other two urban centres (Figure 1 panel C). These differences reflected a more general spatial pattern of low transmission potential at high altitudes which typically present unfavourable conditions for the vectors (Figure 1 panel A); with Vitoria da Conquista at 950 metres (m), Feira de Santana at 200 m and Salvador at 2 m, above sea level.

Confirmed cases presented three CHIKV epidemic waves, characterized by a continuous reduction in total cases per year from 2016 to 2018 (Figure 1 panel C). Importantly, our estimations of transmission potential remained roughly similar throughout the years; thus suggesting that the decreasing trend in the number of confirmed cases is likely a consequence of a gradual accumulation of herd-immunity over the 4 years of continuous circulation in Bahia after its introduction in 2014; similarly to what has been described for ZIKV in Feira de Santana [5].

In this study, we demonstrate the re-introduction of CHIKV-ECSA into Bahia between 2016 and 2018. Bahia was the introductory point of CHIKV-ECSA in the Americas and it is the region in Brazil that seems to have the highest genetic diversity of this genotype. Since the first registered cases in 2014, no new genomic surveillance data have been released. Our phylogenetic reconstructions show the persistence of local lineages in some municipalities and the re-introduction of new epidemiological strains from different Brazilian regions, highlighting a complex dynamic of transmission between epidemic seasons and sampled locations. Several factors including the high connectivity by air and land transport between different regions of the country, vector suitability, insufficient herd immunity and poor living conditions, might be shaping this complex viral spread between different Brazilian regions [4,6]. Focusing on the three main urban centres of Bahia, we also revealed the reconstructed transmission potential of CHIKV has remained reasonably stable throughout the years, such that the observed large reduction in incidence is likely a consequence of an expected, gradual accumulation of herd-immunity over the 4 years since its introduction in 2014.

Due to the co-circulation of different mosquito-borne viruses (i.e. zika, dengue, Mayaro, oropouche, yellow fever viruses) exanthematic disease is increasing in prevalence in Brazil, making it difficult for epidemiological data alone (e.g. case counts) to help us understand the transmission of any one of the viruses. Genomics plays a crucial role in rapidly detecting and tracking mosquito-borne viral transmission. Our study shows that genomic data generated using portable sequencing technology can be employed to assist public health laboratories in monitoring the diversity of circulating mosquito-borne viruses in order to better understand the complex dynamics of viral importation and persistence among regions of Brazil.

## Ethical statement

This research was approved by the Pan American Health Organization Ethics Review Committee (PAHOERC) (Ref. No. PAHO-2016-08-0029). Processing of human samples was approved by the Institutional Review Board from the Fundação Oswaldo Cruz (CEP/CAAE: approval number 1.184.454).

## Author contributions

Conceptualization: Z.P.G.M., J.L., F.M.P., R.C., A.M.B.F., L.C.J.A. and M.G. Data Curation: Z.P.G.M., J.L., F.M.P., A.L.S.M., M.G.; R.V.C., V.F., V.B.N., T.O. Formal Analysis: J.L., V.F., Z.P.G.M., and M.G. Investigation: V.B.N., F.M.P., Z.P.G.M., F.B.N., N.R.C.F., N.R.F., R.V.C., J.L., and M.G. Validation: J.L., L.C.J.A, and M.G. Writing–Original Draft Preparation: Z.P.G.M., J.L., F.M.P., L.C.J.A., and M.G. Resources: Z.P.G.M., F.M.P., A.L.S.M., A.L.A., C.F.C.A., R.V.C., W.K.O. R.S., and J.C. Draft Revision: Z.P.G.M., R.S., F.M.P., F.B.N., N.R.C.F., J.X., A.L.S.M., A.L.A., C.F.C.A., W.K.O., J.C., T.G., N.F., A.M.B.F., R.V.C., J.L., L.C.J.A. and M.G.

## Supplementary Material

Supplemental Material

## Data Availability

New genome sequences obtained in this study have been deposited in GenBank under accession numbers MK156053 - MK156056; MK156058 - MK156064; MK752950 - MK752958.

## References

[1] Nunes MR, Faria NR, de Vasconcelos JM, et al. Emergence and potential for spread of Chikungunya virus in Brazil. BMC Med. 2015;13:102–103. doi: 10.1186/s12916-015-0348-x 25976325 PMC4433093

[2] Naveca FG, Claro I, Giovanetti M, et al. Genomic, epidemiological and digital surveillance of Chikungunya virus in the Brazilian Amazon. PLoS Negl Trop Dis. 2019;7:13–13. 10.1371/journal.pntd.0007065 PMC6424459 30845267

[3] Xavier J, Giovanetti M, Fonseca V, et al. Circulation of Chikungunya virus East/Central/South African lineage in Rio de Janeiro, Brazil. PLoS One. 2019;11:14–16. 10.1371/journal.pone.0217871 PMC6559644 31185030

[4] Brasil. Boletim Epidemiológico - Monitoramento dos casos de dengue, febre de chikungunya e doença aguda pelo vírus Zika até a Semana Epidemiológica 52 de 2018. Ministério da Saúde. Secretaria de Vigilância em Saúde; 2019. Available from http://portalarquivos2.saude.gov.br/images/pdf/2019/janeiro/02/2018-067.pdf.

[5] Lourenço J, de Lima M M, Faria NR, et al. Epidemiological and ecological determinants of Zika virus transmission in an urban setting. Elife. 2017;9:6–29. 10.7554/eLife.29820 PMC5638629 28887877

[6] Lanciotti RS, Kosoy OL, Laven JJ, et al. Genetic and serologic properties of Zika virus associated with an epidemic, Yap state, Micronesia, 2007. Emerg Infect Dis 2008;14:1232–1239. doi: 10.3201/eid1408.080287 18680646 PMC2600394

[7] Lanciotti RS, Calisher CH, Gubler DJ, et al. Rapid detection and typing of dengue viruses from clinical samples by using reverse transcriptase-polymerase chain reaction. J Clin Microbiol. 1992;30:545–551. 1372617 10.1128/jcm.30.3.545-551.1992 PMC265106

[8] Naveca FG, Nascimento VAD, Souza VC, et al. Multiplexed reverse transcription real-time polymerase chain reaction for simultaneous detection of Mayaro, Oropouche, and Oropouche-like viruses. Mem Inst Oswaldo Cruz. 2017;112:510–513. doi: 10.1590/0074-02760160062 28591313 PMC5452489

[9] Lanciotti RS, Kosoy OL, Laven JJ, et al. Chikungunya virus in US travelers returning from India, 2006. Emerg Infect Dis. 2007;13:764–767. doi: 10.3201/eid1305.070015 17553261 PMC2738459

[10] Quick J, Grubaugh ND, Pullan ST, et al. Multiplex PCR method for MinION and Illumina sequencing of Zika and other virus genomes directly from clinical samples. Nat Protoc. 2017;12:1261–1276. doi: 10.1038/nprot.2017.066 28538739 PMC5902022

[11] Fonseca V, Libin PJK, Theys K, et al. A computational method for the identification of Dengue, Zika and Chikungunya virus species and genotypes. PLoS Negl Trop Dis. 2019;8:13–15. 10.1371/journal.pntd.0007231 PMC6527240 31067235

[12] Nguyen LT, Schmidt HA, von Haeseler A, et al. IQ-TREE: a fast and effective stochastic algorithm for estimating maximum-likelihood phylogenies. Mol Biol Evol. 2015;32:268–274. doi: 10.1093/molbev/msu300 25371430 PMC4271533

[13] Suchard MA, Lemey P, Baele G, et al. Bayesian phylogenetic and phylodynamic data integration using BEAST 1.10. Virus Evol. 2018;4:1–016. doi: 10.1093/ve/vey016 PMC6007674 29942656

[14] US National Climate Data Center. Available from https://www7.ncdc.noaa.gov/CDO/cdoselect.cmd?datasetabbv=GSOD.

[15] Obolski U, Perez PN, Villabona-Arenas CJ, et al. MVSE: an R-package that estimates a climate-driven mosquito-borne viral suitability index. Methods Ecol Evol. 2019;4:1–14. 10.1111/2041-210X.13205 PMC7202302 32391139

