# Supplementary material for: Return of the founder Chikungunya virus to its place of introduction into Brazil is revealed by genomic characterization of exanthematic disease cases
Source: Emerg Microbes Infect. 2019 Dec 27;9(1):53–7. doi: 10.1080/22221751.2019.1701954 (PMC6968431; doi:10.1080/22221751.2019.1701954)
Supplement: Supplemental Material [file TEMI_A_1701954_SM8910.zip › Supplementary_Material_for_revision_final.doc]

**Data Availability:** Genomic data obtained from this study. Those data have been already deposited on the National Centre for Biotechnology Information (MK156053-MK156056; MK156058-MK156064; MK752950-MK752958), and will be available upon the manuscript accepted.

>MK156060

cgtctgcccgatgcgcagcgcagaagatcccgagagactcgccaattatgcgagaaagctagcatctgctgcaggaaaagtcctggacagaaacatctctggaaagatcggggacttacaagcagtaatggccgtgccagacacggagacgccaacattctgcttgcacacagatgtctcatgtagacagagagcagacgtcgcgatataccaagacgtctatgctgtacacgcacccacgtcgctataccaccaggcgattaaaggggtccgagtggcgtactgggtagggttcgacacaaccccgttcatgtacaatgccatggcgggtgcctacccctcatattcgacaaactgggcagacgagcaggtactgaaggctaagaacataggattatgttcaacagacctgacggaaggtagacgaggtaaattgtctattatgagagggaagaagctaaaaccgtgtgaccgtgtgctgttctcagtagggtcaacgctctacccggaaagccgcaagctacttaagagctggcacttaccatcggtgttccatctaaagggcaaactcagcttcacatgccgctgtgatacagtggtttcgtgcgagggctacgtcgttaagagaataacgatgagcccaggcctttatggaaaaaccacagggtatgcggtaacccaccacgcagacggattcttgatgtgcaagactaccgacacggttgacggcgaaagagtgtcattctcggtgtgcacatacgtgccggcgaccatttgtgatcaaatgaccggcatccttgctacagaagtcacgccggaggatgcacagaagctgttggtggggctgaaccagagaatagtggttaacggcagaacgcaacggaatacgaacaccatgaaaaactatctgcttcccgtggtcgcccaagccttcagtaagtgggcaaaggagtgccggaaagacatggaagatgaaaaactcctgggggtcagagaaagaacactgacctgctgctgtctatgggcattcaagaagcagaaaacacacacggtctacaagaggcctgatacccagtcaattcagaaggttcaggccgagtttgacagctttgtggtaccgagcctgtggtcgtccgggttgtcaatccccttgaggactagaatcaaatggttgttaagcaaggtgccaaaaaccgacctgatcccatatagcggggacgcccaagaagcccgggacgcagaaaaagaagcagaggaagaacgagaagcagaactgactcgtgaagccctaccacctctacaggcagcacaggaagatgttcaggtcgaaatcgacgtggaacagcttgaggacagagcgggtgcaggaataatagagactcctagaggagctatcaaagttactgcccaaccaacagaccacgtcgtgggagagtacctggtactttccccgcagaccgtactacggagccagaagctcagcctaattcacgctttggcggagcaagtgaagacgtgnnnncacagcggacgagcagggaggtacgcggtcgaagcgtacgacggccgagtcctagtgccctcaggctatgcaatctcgcctgaagacttccagagtctaagcgaaagcgcaacgatggtgtataacgaaagagagttcgtaaacagaaagctacaccacattgcgatgcacggaccagccctgaacaccgacgaagagtcgtatgagctggtgagggcagagaggacagaacacgagtacgtctacgatgtggaccagagaagatgctgtaagaaggaagaagctgcaggactggtactggtgggcgacttgactaatccgccctaccacgaattcgcatatgaagggctaaaaatccgccctgcctgcccatacaaaattgcagtcataggagtcttcggagtaccaggatctggcaagtcagctattatcaagaacctagttaccaggcaagacctggtgactagcggaaagaaagaaaactgccaagaaatcaccaccgacgtgatgagacagagaggtctagagatatctgcacgtacggttgactcgctgctcttgaatggatgcaacaggccagtcgacgtgttgtacgtagacgaggcgtttgcgtgccactctggaacgttacttgctttgatcgccttggttagaccaaggcagaaagttgtactctgtggtgacccgaagcagtgcggcttcttcaatatgatgcagatgaaagtcaactataatcacaacatctgcacccaagtgtaccacaaaagtatctccaggcggtgtacactgcctgtgaccgccattgtgtcatcgttncattacgaaggcaaaatgcgcactacgaatgagtacaacaagccgattgtagtggacactacaggctcaacaaaacctgacgctggagatctcgtgttaacgtgcttcagagggtgggttaaacaactgcaaattgactatcgtggatacgaggtcatgacagcagccgcatcccaagggttaaccagaaaaggagtttatgcagttaggcaaaaagttaatgaaaacccgctctatgcatcaacgtcagagcacgtcaacgtactcctaacacgtacggaaggtaaactggtatggaagacactttccggtgacccgtggataaagacgctgcagaacccaccgaaaggaaacttcaaagcaactatcaaggagtgggaggtggagcatgcatcaataatggcgggcatctgcagtcaccaaatgacctttgatacattccaaaacaaagccaatgtttgttgggctaagagcttggtccctatcctcgaaacagcggggataaaactaaatgataggcagtggtcccagataattcaagccttcaaagaagataaagcatactcacctgaagtagccctgaatgaaatatgcacgcgcatgtatggggtggatttagacagtgggctattttctaaaccattggtgtctgtgtactactcggataaccactgggataataggcctggagggaagatgttcggattcaaccccgaggcagcatccattctagaaagaaagtatccattcacaaaagggaagtggaacatcaacaagcagatctgtgtgaccaccaggaggatagaagactttaaccctaccaccaatattataccggccaataggagactaccacactcattagtggccgaacaccgcccagtaaaaggggaaagaatggaatggctggttaataagataaacggccaccacgtgctcctggtcagtggctataatcttgtactgcctactaagagagtcacttgggtagcgccactaggtgtccgcggagcggactacacatacaacctagagttgggtctgccagcaacgcttggcaggtatgacctagtggtcataaacatccacacaccttttcgcatacaccattaccaacagtgcgtagaccacgcaatgaaactgcaaatgctcgggggtgactcattgagactgctcaaaccgggtggctctctattgannnnnnnnnnnnnnnnnnnnnntagaaccagtgaacgagtcatctgcgtgttgggacgcaagttcagatcgtctagagcgttgaaaccaccatgtgtcaccagcaacactgagatgtttttcctattcagcaactttgacaatggcagaaggaatttcacaactcatgtcatgaataatcaactgaatgcagcctttgtaggacaggccacccgagcaggatgtgcaccgtcgtaccgggtaaaacgcatggacatcgcgaagaacgatnnagngtgcnnnnnnnnnnnnnnnnnnnnnnnnnnnnnnnnnnnnnnnnnnnnnnnnnnnnnnnnnnacnaaaaatggccggagtcctttaagaatagtgcaacaccagtgggaaccgcaaaaacagtcatgtgcggtacgtatccagtaatccacgccgttggaccgaatttctctaattatttggaatctgaaggggaccgggaattggcagctgcctatcgagaagtcgcaaaggaagtaactagactgggagtaaatagtgtagctatacctctcctctccacaggtnnnnnnnnnnnnnnnnnnnnnnnnnnnnnnnnnnnnnnnnnnnnnnnnnnnnnnnnnnnnnnnnnnnnnnnnnnnnnnnnnnnnnnnnnnnnnnnnnnnnnnnnnnnnnnnnnnnnnnnnnnnnnnnnnnnnnnnnnnnnnnnnnnnnnnnnnnnnnnnnnnnnnnnnnnnnnnnnnnnnnnnnnnnnnnnnnnnnnnnnnnnnnnnnnnnnnnnnnnnnnnnnnnnnnnnnnnnnnnnnnnnnnnnnnnnnnnnnnnnnnnnnnnnnnnnnnnnnnnnnnnnnnnnnnnggcagtggacatggcagagatatatactatgtggccaaagcaaacagaggccaacgagcaagtctgcctatatgccctgggggaaagtattgaatcgatcaggcagaaatgcccggtggatgatgcagacgcatcgtctcctccaaaaactgtcccgtgcctttgccgttacgccatgactccggaacgcgtcaccagacttcgcatgaaccacgtcacaagtataattgtgtgttcctcgtttccccttccaaagtacaaaatagaaggagtgcaaaaagtcaaatgctctaaggtaatgctatttgaccacaacgtgccatcacgcgtaagtccaagggaatatagatcttccccggaggctgcacaggaagcgagtacaaccacgtcactgacgcatagccaattcgacctaagcgttgatggcgagatactacccgtcccgtcagatctggatgctgacgccccagccctagaaccagctctagacgacagggcgacacacacgttgccatccacaaccggaaaccttgcggccgtgtctgactgggtaatgagcaccgtacctgtcgcgccgcccagaagaaggcgagggagaaacctgactgtgacatgcgacgagagagaagggaacataacacccatggctagcgtccgattctttagggcagagctgcgtccggtcgtacaagaaacagcggagacgcgtgacacagcaatgtctcttcaggcaccgccgagtaccgccacggaactgaatcatccgccgatctccttcggagcaccaagnnnnnnnnnnnnnnnnnnnnnnnnnnnnnnnnnnnnnnnnnnnnnnnnnnnnnnnnnnnnnnnnnnnnnnnnnnnnnnnnnnnnnnnnnnnnnnnnnnnnnnnnnnnnnnnnnnnnnnnnnnnnnnnnnnnnnnnnnnnnnnnnnnnnnnnnnnnnagacagggcaggtgggtatatattctcgtcggacaccggtccaggtcatttacaacagaagtcagtacgccagtcagtgctgccggtgaacaccctggaggaagtccacgtggagaagtgttacccacctaagttggatgaagtaaaggagcaactattacttaagaaactccaggagagtgcatccatggccaacagaagcaggtaccagtcgcgcaaagtagaaaacatgaaagcaacaatcatccagagactaaagagaggctgtagactatacttaatgtcagagacctcaaaagtccctacttaccggaccacatatccggcgcctgtgtactcgcctccggtcaacgtccgactgtccaaccccgagtccgcagtggcagcatgcaatgagttcctagctagaaactatccaactgtctcatcataccaaattaccgacgagtatgatgcatatttagacatggtggacgggtcggagagttgcctggatcgagcgacattcaatccgtcaaaacttaggagctacccgaaacagcacgcttaccatgcgccctccatcagaagcgctgtaccgtccccattccagaacacactacagaatgtactggcagcagccacgaaaagaaactgcaacgtcacacagatgagggaattacccactttggactcagcagtattcaacgtggagtgttttaaaaaattcgcatgcaaccaagaatactgggaagaatttgctgccagccctattaggataacaactgagaatttaacaacctatgttactaaactaaaagggccaaaagcagcagcgctatttgcnnnnnnnnnnnnnnnnnnnccactgcaggaagtaccaatggataggttcacagtagatatgaaaagggacgtgaaggtgactcctggtacaaagcacacagaggaaagacctaaggtgcaggttatacaggcggctgaacccttggcgacagcatacctatgtgggattcacagagagctggttaggaggctgaacgccgtcctcctacccaatgtacatacactatttgatatgtctgccgaggatttcgatgccatcatagccgcacactttaagccaggagacactgttttagaaacggacatagcctcctttgacaagagccaagatgattcacttgcgcttactgccttgatgttgttagaagatttaggggtggatcactccctgttggacttgatagaggccgcttttggagagatttccagctgtcannnnnnnnnnnnnnnnnnnnnnnnnnnnggcgccatgatgaaatctggtatgttcctaactctgttcgtcaacacattgctaaacatcaccatcgctagccgcgtgctggaagatcgtctgacaaaatccgcgtgcgcggccttcatcggcgacgacaacataatacatggagtcgtctccgatgaattgatggcagacagatgcgccacttggatgaacatggaagtgaagatcatagatgcagttgtatccttgaaagccccttacttttgtggagggtttatactgcacgatactgtgacgggaacagcctgcagagtggcagacccgctaaaaaggctattcaaactgggcaaaccgctagcggcaggtgacgaacaagatgaagatagaagacgagcgctggctgacgaagtgatcagatggcaacgaacagggctaattgatgagttggagaaagcggtatactctaggtatgaagtgcagggtatatcagttgtggtaatgtccatggccacctttgcaagctccagatctaacttcgagaagctcagaggacccnnnnnnnnnnnnnnnnnnnnnnnnnnnnnnnnnnnnnnnnnnnnnnnnnnnnnnnnnnnnnnnnnnnnnnnnnnnnnnnnnnnnnnnnnnnnnnnnnnnnnnnnnnnnnnnnnnnnnnnnnnnnnnnnnnnnnnnnnnnnnnnnnnnnnnnnnnnnnnnnnnnnnnnnnnnnnnnnnnnnnnnnnnnnnnnnnnnnnnnnnnnnnnnnnnnnnnnnnnnnnnnnnnnnnnnnnnnnnnnnnnnnnttggtgggggacaaagtaatgaaaccagcacacgtaaaggggaccatcgataacgcggacctggccaaactggcctttaagcggtcatctaagtatgaccttgaatgcgcgcagatacccgtgcacatgaagtccgacgcttcgaagttcacccatgagaaaccagaggggtactacaactggcaccacggagcagtacagtattcaggaggccggttcaccatccccacaggtgctggcaaaccaggggacagcggcagaccgatcttcgacaacaagggacgcgtggtggccatagtcttaggaggagctaatgaaggagcccgtacagccctctcggtggtgacctggaataaagacattgtcactaaaatcacccccgagggggccgaagagtggagtcttgccatcccagtcatgtgcctgctggcaaataccacgttcccctgctcccagcccccttgcactccctgctgctatgaaagggaaccagaggaaaccctacgcatgcttgaggacaacgtcatgagacctgggtactatcagctgctacaagcattaaagacggatgatagccacgattggaccaagctgcgttacatggacaaccacacgccagcggacgcagagagggcggggctatttgtaagaacatcagcaccgtgcacgattactggaacaatgggacacttcatcctgacccgatgtccgaaaggggaaactctgacggtgggattcactgacagtaggaagatcagtcactcatgtacgcacccatttcaccacgaccctcctgtgataggccgggagaaattccattcccgaccgcagcacngtaaagagctgccttgcagcacgtacgtgcagagcaccgccgcaactaccgaggagatagaggtacacatgcccccagacacccctgatcgcacactgatgtcacaacagtccggcaacgtaaagatcacagttaatggccagacggtgcggtacaagtgtaattgcggtggctcaaatgaaggactaataactacagacaaagtgattaataactgcaaagttgatcaatgtcatgccgcggtcaccaatcacaaaaagtggcagtacaactcccctctggtcccgcgtaatgctgaacttggggaccgaaaaggaaaaatccacatcccgtttccgctggcaaatgtaacatgcagggtgcctaaagcaaggaaccccaccgtgacgtacgggaaaaaccaagtcatcatgctactgtatcccgaccacccaacactcctgtcctaccggaacatgggagaagaaccaaactaccaagaagagtgggtgacgcataagaaggaagtcgtgctaaccgtgccgactgaagggctcgnnnnnnnnnnnnnnnnnnnnnnnnnnnnnnnnnnnnnnnnnnnnnnnnnnnnnnnnnnnnnnnnnnnnnnnnnnnnnnnnnnnnnnnnnnnnnnnnnnnnnnnnnnnnnnnnnnnnnnnnnnnnnnnnnnnnnnnnnnnnnnnnnnnnnnnnnnnnnnnnnnnnnnnnnnnnnnnnnnnnnnnnnnnnnnnnnnnnnnnnnnnnnnnnnnnnnnnnnnnnnnnnnnnnnnnnnnnnnnnnnnnnnnnnnnnnnnnnnnnnnnnnnnnnnnnnnnnnnnnnnnnnnnnnnnnnnnnnnnnnnnnnnnnnnnnnnnnnnnnnnnnnnnnnnnnnnnnnnnnnnnnnnnnnnnnnnnnnnnnnnnnnnnnnnnnnnnnnnnnnnnnnnnnnnnnnnnnnnnnnnnnnnnnnnnnnnnnnnnnnnnnnnnnnnnnnnnnnnnnnnnnnnnnnnnnnnnnnnnnnnnnnnnnagcgcgtacgaacacgtaacagtgatcccgaacacggtgggagtaccgtataagactctagtcaatagaccgggctacagtcccatggtattggagatggaactactgtcagtcactttggagccaacgctatcgcttgattacatcacgtgcgagtacaaaaccgttatcccgtctccgtacgtgaaatgctgcggtacagcagagtgcaaggacaaaaacctacctgactacagctgtaaggtcttcaccggcgtctacccatttatgtggggcggagcctgccaaattcattgtggggccaatgtcttcagcctggacacctttcgacaacaaaattgtggtgtacaaaggtgacgtctataacatggactacccgccctttggcgcaggaagaccaggacaatttggcgatatccaaagtcgcacacctgagagtacagacgtctatgctaatacacaactggtactgcagagaccggctgcgggtacggtacatgtgccatactctcaggcaccatctggctttaagtattggctaaaagaacgaggggcgtcgctgcagcacacagcaccatttggctgccaaatagcaacaaacccggtaagagcggtgaattgcgccgtagggaacatgcccatctccatcgacataccgga

>MK156053

cgtctgcccgatgcgcagcgcagaagatcccgagagactcgccaattatgcgagaaagctagcatctgctgcaggaaaagtcctggacagaaacatctctggaaagatcggggacttacaagcagtaatggccgtgccagacacggagacgccaacattctgcttgcacacagatgtctcatgtagacagagagcagacgtcgcgatataccaagacgtctatgctgtacacgcacccacgtcgctataccaccaggcgattaaaggggtccgagtggcgtactgggtagggttcgacacaaccccgttcatgtacaatgccatggcgggtgcctacccctcatattcgacaaactgggcagacgagcaggtactgaaggctaagaacataggattatgttcaacagacctgacggaaggtagacgaggtaaattgtctattatgagagggaagaagctaaaaccgtgtgaccgtgtgctgttctcagtagggtcaacgctctacccggaaagccgcaagctacttaagagctggcacttaccatcggtgttccatctaaagggcaaactcagcttcacatgccgctgtgatacagtggtttcgtgcgagggctacgtcgttaagagaataacgatgagcccaggcctttatggaaaaaccacagggtatgcggtaacccaccacgcagacggattcttgatgtgcaagactaccgacacggttgacggcgaaagagtgtcattctcggtgtgcacatacgtgccggcgaccatttgtgatcaaatgaccggcatccttgctacagaagtcacgccggaggatgcacagaagctgttggtggggctgaaccagagaatagtggttaacggcagaacgcaacggaatacgaacaccatgaaaaactatctgcttcccgtggtcgcccaagccttcagtaagtgggcaaaggagtgccggaaagacatggaagatgaaaaactcctgggggtcagagaaagaacactgacctgctgctgtctatgggcattcaagaagcagaaaacacacacggtctacaagaggcctgatacccagtcaattcagaaggttcaggccgagtttgacagctttgtggtaccgagcctgtggtcgtccgggttgtcaatccccttgaggactagaatcaaatggttgttaagcaaggtgccaaaaaccgacctgatcccatatagcggggacgcccaagaagcccgggacgcagaaaaagaagcagaggaagaacgagaagcagnnnnnnnnnnnnnnnnnnnnnnnnnnnnnnnnnnnnnnnnnnnnnnnnnnnnnnnnnnnnnnnnnnnnnnnnnnnnnnnnnnnnnnnnnnnnnnnnnnnnnnnnnnnnnnnnnnnnnnnnnnnnnnnnnnnnnnnnnnnnnnnnnnnnnnnnnnnnnnnnnnnnnnnnnnnnnnnnnnnnnnnnnnnnnnnnnnnnnnnnnnnnnnnnnnnnnnnnnnnnnnnnnnnnnnnnnnnnnnnnnnnnnnnnnnncacagcggacgagcagggaggtacgcggtcgaagcgtacgacggccgagtcctagtgccctcaggctatgcaatctcgcctgaagacttccagagtctaagcgaaagcgcaacgatggtgtataacgaaagagagttcgtaaacagaaagctacaccacattgcgatgcacggaccagccctgaacaccgacgaagagtcgtatgagctggtgagggcagagaggacagaacacgagtacgtctacgatgtggaccagagaagatgctgtaagaaggaagaagctgcaggactggtattggtgggcgacttgactaatccgccctaccacgaattcgcatatgaagggctaaaaatccgccctgcctgcccatacaaaattgcagtcataggagtcttcggagtaccaggatctggcaagtcagctattatcaagaacctagttaccaggcaagacctggtgactagcggaaagaaagaaaactgccaagaaatcaccaccgacgtgatgagacagagaggtctagagatatctgcacgtacggttgactcgctgctcttgaatggatgcaacaggccagtcgacgtgttgtacgtagacgaggcgtttgngtgnnnnnnnnnnnnnnnnnnnnnnnnnnnnnnnnnnnnnnnnnnnnnnnnnnnnnnnnnnnnnnnnnnnnnnnnnnnnnnnnnnnnnnnnnnnnnnnnnnnnnnnnnnnnnnnnnnnnnnnnnnnnnnnnnnnnnnnnnnnnnnnnnnnnnnnnnnnnnnnnnnnnnnnnnnnnnnnnnnnnnnnnnnnnnnnnnnnnnnnnnnnnnnnnnnnnnnnnnnnnnnnnnnnnnnnnnagtacaacaagccgattgtagtggacactacaggctcaacaaaacctgacgctggagatctcgtgttaacgtgcttcagagggtgggttaaacaactgcaaattgactatcgtggatacgaggtcatgacagcagccgcatcccaagggttaaccagaaaaggagtttatgcagttaggcaaaaagttaatgaaaacccgctctatgcatcaacgtcagagcacgtcaacgnnnnnnnnnnnnnnnnnnnnnnnnnnnnnnnnnnnnnnnnnnnnnnnnnnnnnnnnnnnnnnnnnnnnnnnnnnnnnnnnnnnnnnnnnnnnnnnnnnnnnnnnnnnnnnnnnnnnnnnnnnnnnnnnnnnnnnnnnnnnnnnnnnnnnnnnnnnnnnnnnnnnnnnnnnnnnnnnnnnnnnnnnnnnnnnnnnnnnnnnnnnnnnnnnnnnnnnnnnnnnnnnnnnnnnnnnnnngggataaaactaaatgataggcagtggtcccagataattcaagccttcaaagaagataaagcatactcacctgaagtagccctgaatgaaatatgcacgcgcatgtatggggtggatttagacagtgggctattttctaaaccattggtgtctgtgtactactcggataaccactgggataataggcctggagggaagatgttcggattcaaccccgaggcagcatccattctagaaagaaagtatccattcacaaaagggaagtggaacatcaacaagnnnnnnnnnnnnnnnnnnnnnnnnnnnnnnnnnnnnnnnnnnnnnnnnnnnnnnnnnnnnnnnnnnnnnnnnnnnnnnnnnnnnnnnnnnnnnnnnnnnnnnnnnnnnnnnnnnnnnnnnnnnnnnnnnnnnnnnnnnnnnnnnnnnnnnnnnnnnnnnnnnnnnnnnnnnnnnnnnnnnnnnnnnnnnnnnnnnnnnnnnnnnnnnnnnnnnnnnnnnnnnnnnnnnnnnnnnnnnnnnnnnnnnnnnnnnnnnnnnnnnnnnnnnnnnnnnnnnnnnnnnnnnnnnnnnnnnnnnnncataaacatccacacaccntttcgcatacaccattaccaacagtgcgtagaccacgcaatgaaactgcaaatgctcnggggtgactcattgagactgctcaaaccgggtggctctctattgatcagagnatatggttacgcagatagaaccagtgaacgagtcatctgcgtgttgggacgcaagttcagatcgtctagagcgttgaaaccaccatgtgtcaccagcaacactgagatgtttttcctattcagcaactttgacaatggcagaaggaatttcacaactcatgtcatgaataatcaactgaatgcagcctttgtaggacaggccacccgagcaggatgtgcaccgtcgtaccgggtaaaacgcatggacatcgcgaagaacgatnnagngtgcnnnnnnnnnnnnnnnnnnnnnnnnnnnnnnnnnnnnnnnnnnnnnnnnnnnnnnnnnnacaaaaaatggccggagtcctttaagaatagtgcaacaccagtgggaaccgcaaaaacagtcatgtgcggtacgtatccagtaatccacgccgttggaccgaacttctctaattatttggaatctgaaggggaccgggaattggcagctgcctatcgagaagtcgcaaaggaagtaactagactgggagtaaatagtgtagctatacctctcctctccacaggtgnnnnnnnnnnnnnnnnnnnnnnnnnnnnnnnnnnnnnnnnnnnnnnnnnnnnnnnnnnnnnnnnnnnnnnnnnnnnnnnnnnnnnnnnnnnnnnnnnnnnnnnnnnnnnnnnnnnnnnnnnnnnnnnnnnnnnnnnnnnnnnnnnnnnnnnnnnnnnnnnnnnnnnnnnnnnnnnnnnnnnnnnnnnnnnnnnnnnnnnnnnnnnnnnnnnnnnnnnnnnnnnnnnnnnnnnnnnnnnnnnnnnnnnnnnnnnnnnnnnnnnnnnnnnnnnnnnnnnnnnnnnnnnnnnnnnnnnnnnnnnnnnnnnnnnnnnnnnnnnnnnnnnnnnnnnnnnnnnnnnnnnnnnnnnnnnnnnnnnnnnnnnnnnnnnnnnnnnnnnnnnnnnnnnnnnnnnnnnnnnnnnnnnnnnnnnnnnnnnnnnnnnnnnnnnnnnnnnnnnnnnnnnnnnnnnnnnnnnnnnnnnnnnnnnnnnnnnnnnnnnnnnnnnnnnnnnnnnnnnnnnnnnnncaagtataattgtgtgttcctcgtttncccttccaaagtacaaaatagaaggagtgcnaaaagtcaaatgctctaaggtaatgctatttgaccacaacgtgccatcacgcgtaagtncaagggaatatagatcttncccggaggctgcacaggaagcgagtacaaccacgtcactgacgcatagccaattcgacctaagcgttganggcgagatactacccgtnccgtcagatctngatgctgacgncccagccctagaaccagctcnnnnnnnnnnnnnnnnnnnnnnnnnnnnnnnnnnnnnnnnnnnnnnnnnnnnnnnnnnnnnnnnnnnnnnnnnnnnnnnnnnnnnnnnnnnnnnnnnnnnnnnnnnnnnnnnnnnnnnnnnnnnnnnnnnnnnnnnnnnnnnnnnnnnnnnnnnnnnnnnnnnnnnnnnnnnnnnnnnnnnnnnnnnnnnnnnnnnnnnnnnnnnnnnnnnnnnnnnnnnnnnnnnnnnnnnnnnnnnnnnncaggcaccgccgagtaccgccacggaactgaatcatccgccgatctccttcggagcaccaagcgagacgttccccattacatttggggacttcaacgaaggagaaatcgaaagcttgtcttctgagctgctaactttcggagacttcctacccggagaagtggatgacttgacagatagcgactggtccacgtgctcagacacggacgacgagttactagacagggcaggtgggtatatattctcgtcggacaccggtccaggtcatttacaacagaagtcagtacgccagtcagtgctgccggtgaacaccctggaggaagtccacgaggagaagtgttacccacctaagttggatgaagtaaaggagcaactattacttaagaaactccaggagagtgcatccatggccaacagaagcaggtaccagtcgcgcaaagtagaaaacatgaaagcaacaatcatccagagactaaagagaggctgtagactatacttaatgtcagagacctcaaaagtccctacttaccggaccacatatccggcgcctgtgtactcgcctccggtcaacgtccgactgtccaaccccgagtccgcagtggcagcatgcaatgagttcctagctagaaactatccaactgtctcatcataccaaattaccgacgagtatgatgcatatttagacatggtggacgggtcggagagttgcctggatcgagcgacattcaatccgtcaaaacttaggagctacccgaaacagcacgcttaccatgcgccctccatcagaagcgctgtaccgtccccattccagaacacactacagaatgtactggcagcagccacgaaaagaaactgcaacgtcacacagatgagggaattacccactttggactcagcagtattcaacgtggagtgttttaaaaaattcgcatgcaaccaagaatactgggaagaatttgctgccagccctattaggataacaactgagaatttaacaacctatgttactaaactaaaagggccaaaagcagcagcgctatttgcnnnnnnnnnnnnnnnnnnnnnactgcaggangtaccaatggataggttcacagtagatatgaaaagggacgtgaaggtgactcctggtacaaagcacacagaggaaagacctaaggtgcaggttatacaggcggctgaacccttggcgacagcatacctatgtgggattcacagagagctggttaggaggctgaacgccgtcctcctacccaatgtacatacactatttgatatgtctgccgaggatttcgatgccatcatagccgcacactttaagccaggagacactgttttagaaacggacatagcctcctttgacaagagccaagatgattcacttgcgcttactgccttgatgttgttagaagatttaggggtggatcactccctgttggacttgatagaggccgcttttggagagatttccagctgtcatctaccnnnnnnnnnnnnnnnnnnnnnnggcgccatgatgaaatctggtatgttcctaactctgttcgtcaacacattgctaaacatcaccatcgctagccgcgtgctggaagatcgtctgacaaaatccgcgtgcgcggccttcatcggcgacgacaacataatacatggagtcgtctccgatgaattgatggcagacagatgcgccacttggatgaacatggaagtgaagatcatagatgcagttgtatccttgaaagccccttacttttgtggagggtttatactgcacgatactgtgacgggaacagcctgcagagtggcagacccgctaaaaaggctattcaaactgggcaaaccgctagcggcaggtgacgaacaagatgaagatagaagacgagcgctggctgacgaagtgatcagatggcaacgaacagggctaattgatgagttggagaaagcggtatactctaggtatgaagtgcagggtatatcagttgtggtaatgtccatggccacctttgcaagctccagatctaacttcgagaagctcagaggaccccagttaataaactgacaatgcgcgcggtaccccaacagaagccacgcaggaatcggaagaataagaagcaaaagcaaaagcgacaggcgccacaaaacaacacaaancaaaanaagcagccgcctaaaaagaaaccagctcaaaagnaaaagaagccgggccgcnnagagaggatgtgcatgaaaatcgaaaatgattgtanttttgaagtcaagtatgaaggtaaggtaacaggttacgcgtgcttggtgggggacaaagtaatgaaaccagcacacgtaaaggggaccatcgataacgcggacctggccaaactggcctttaagcggtcatctaagtatgaccttgaatgcgcgcagatacccgtgcacatgaagtccgacgcttcgaagttcacccatgagaaaccagaggggtactacaactggcaccacggagcagtacagtattcaggaggccggttcaccatccccacaggtgctggcaaaccaggggacagcggcagaccgatcttcgacaacaagggacgcgtggtggccatagtcttaggaggagctaatgaaggagcccgtacagccctctcggtggtgacctggaataaagacattgtcactaaaatcacccccgagggggccgaagagtggagtcttgccatcccagtcatgtgcctgctggcaaataccacgttcccctgctcccagcccccttgcactccctgctgctatgaaagggaaccagaggaaaccctacgcatgcttgaggacaacgtcatgagacctgggtactatcagctgctacaagcattaaagacggatgatagccacgattggaccaagctgcgttacatggacaaccacacgccagcggacgcagagagggcggggctatttgtaagaacatcagcaccgtgcacgattactggaacaatgggacacttcatcctgacccgatgtccgaaaggggaaactctgacggtgggattcactgacagtaggaagatcagtcactcatgtacgcacccatttcaccacgaccctcctgtgataggccgggagaaattccattcccgaccgcagcacggtaaagagctgccttgcagcacgtacgtgcagagcaccgccgcaactaccgaggagatagaggtacacatgcccccagacacccctgatcgcacactgatgtcacaacagtccggcaacgtaaagatcacagttaatggccagacggtgcggtacaagtgtaattgcggtggctcaaatgaaggactaataactacagacaaagtgattaataactgcaaagttgatcaatgtcatgccgcggtcaccaatcacaaaaagtggcagtacaactcccctctggtcccgcgtaatgctgaacttggggaccgaaaaggaaaaatccacatcccgtttccgctggcaaatgtaacatgcagggtgcctaaagcaaggaaccccaccgtgacgtacgggaaaaaccaagtcatcatgctactgtatcccgaccacccaacactcctgtcctaccggaacatgggagaagaaccaaactaccaagaagagtgggtgacgcataagaaggaagtcgtgctaaccgtgccgactgaagggctcgaggtcacgtggggtaacaacgagccgtataagtattggccgcagttatctacaaacggtacagcccatggccacccgcatgagataattctgtattattatgagctgtaccctactatgactgtagtagttgtgtcagtggcctcgttcgtactcctgtcgatggtgggtgtggcagtggggatgtgcatgtgtgcacgacgcagatgcannnnnnnnnnnnnnnnnnnnnnnnnnnnnnnnnnnnnnnnnnnnnnnnnnnnnnnnnnnnnnnnnnnnnnnnnnnnnnnnnnnnnnnnnnnnnnnnnnnnnnnnnnnnnnnnnnnnnnnnnnnnnnnnnnnnnnnnnnnnnnnnnnnnnnnnnnnnnnnnnnnnnnnnnnnnnnnnnnnnnnnnnnnnnnnnnnnnnnnnnnnnnnnnnnnnnnnnnnnnnnnnnnnnnnnnnnnnnnnnnnngtgcccacactgtgagcgcgtacgaacacgtaacagtgatcccgaacacggtgagagtaccgtataagactctagtcaatagaccgggctacagtcccatggtattggagatggaactactgtcagtcactttggagccaacgctatcgcttgattacatcacgtgcgagtacaaaaccgttatcccgtctccgtacgtgaaatgctgcggtacagcagagtgcaaggacaaaaacctacctgactacagctgtaaggtcttcaccggcgtctacccatttatgtggggcggagcctgccaaattcattgtggggccaatgtcttcagcctggacacctttcgacaacaaaattgtggtgtacaaaggtgacgtctataacatggactacccgccctttggcgcaggaagaccaggacaatttggcgatatccaaagtcgcacacctgagagtacagacgtctatgctaatacacaactggtactgcagagaccggctgcgggtacggtacatgtgccatactctcaggcaccatctggctttaagtattggctaaaagaacgaggggcgtcgctgcagcacacagcaccatttggctgccaaatagcaacaaacccggtaagagcggtgaattgcgccgtagggaacatgcccatctccatcgacataccgga

>MK156054

cgtctgcccgatgcgcagcgcagaagatcccgagagactcgccaattatgcgagaaagctagcatctgctgcaggaaaagtcctggacagaaacatctctggaaagatcggggacttacaagcagtaatggccgtgccagacacggagacgccaacattctgcttgcacacagatgtctcatgtagacagagagcagacgtcgcgatataccaagacgtctatgctgtacacgcacccacgtcgctataccaccaggcgattaaaggggtccgagtggcgtactgggtagggttcgacacaaccccgttcatgtacaatgccatggcgggtgcctacccctcatattcgacaaactgggcagacgagcaggtactgaaggctaagaacataggattatgttcaacagacctgacggaaggtagacgaggtaaattgtctattatgagagggaagaagctaaaaccgtgtgaccgtgtgctgttctcagtagggtcaacgctctacccggaaagccgcaagctacttaagagctggcacttaccatcggtgttccatctaaagggcaaactcagcttcacatgccgctgtgatacagtggtttcgtgcgagggctacgtcgttaagagaataacgatgagcccaggcctttatggaaaaaccacagggtatgcggtaacccaccacgcagacggattcttgatgtgcaagactaccgacacggttgacggcgaaagagtgtcattctcggtgtgcacatacgtgccggcgaccatttgtgatcaaatgaccggcatccttgctacagaagtcacgccggaggatgcacagaagctgttggtggggctgaaccagagaatagtggttaacggcagaacgcaacggaatacgaacaccatgaaaaactatctgcttcccgtggtcgcccaagccttcagtaagtgggcaaaggagtgccggaaagacatggaagatgaaaaactcctgggggtcagagaaagaacactgacctgctgctgtctatgggcattcaagaagcagaaaacacacacggtctacaagaggcctgatacccagtcaattcagaaggttcaggccgagtttgacagctttgtggtaccgagcctgtggtcgtccgggttgtcaatccccttgaggactagaatcaaatggttgttaagcaaggtgccaaaaaccgacctgatcccatatagcggggacgcccaagaagcccgggacgcagaaaaagaagcagaggaagaacgagaagcagnnnnnnnnnnnnnnnnnnnnnnnnnnnnnnnnnnnnnnnnnnnnnnnnntcaggtcgaaatcgacgtggaacagcttgaggacagagcgggtgcaggaataatagagactcctagaggagctatcaaagttactgcccaaccaacagaccacgtcgtgggagagtacctggtactttccccgcagaccgtactacggagccagaagctcagcctaattcacgctttggcggagcaagtgaagacgtgcacgcacagcggacgagcagggaggtacgcggtcgaagcgtacgacggccgagtcctagtgccctcaggctatgcaatctcgcctgaagacttccagagtctaagcgaaagcgcaacgatggtgtataacgaaagagagttcgtaaacagaaagctacaccacattgcgatgcacggaccagccctgaacaccgacgaagagtcgtatgagctggtgagggcagagaggacagaacacgagtacgtctacgatgtggaccagagaagatgctgtaagaaggaagaagctgcaggactggtattggtgggcgacttgactaatccgccctaccacgaattcgcatatgaagggctaaaaatccgccctgcctgcccatacaaaattgcagtcataggagtcttcggagtaccaggatctggcaagtcagctattatcaagaacctagttaccaggcaagacctggtgactagcggaaagaaagaaaactgccaagaaatcaccaccgacgtgatgagacagagaggtctagagatatctgcacgtacggttgactcgctgctcttgaatggatgcaacaggccagtcgacgtgttgtacgtagacgaggcgtttgcgtgccactctggaacgttacttgctttgatcgccttggttagaccaaggcagaaagttgtactctgtggtgacccgaagcagtgcggcttcttcaatatgatgcagatgaaagtcaactataatcacaacatctgcacccaagtgtaccacaaaagtatctccaggcggtgtacactgcctgtgaccgccattgtgtcatcgttacattacgaaggcaaaatgcgcactacgaatgagtacaacaagccgattgtagtggacactacaggctcaacaaaacctgacgctggagatctcgtgttaacgtgcttcagagggtgggttaaacaactgcaaattgactatcgtggatacgaggtcatgacagcagccgcatcccaagggttaaccagaaaaggagtttatgcagttaggcaaaaagttaatgaaaacccgctctatgcatcaacgtcagagcacgtcaacgtactnctaacncgtacggaaggtaaactggtatggaagacactttccggtgacccgtggataaagacgctgcagaacccaccgaaaggaaacttcaaagcaactatcaaggagtgggaggtggagcatgcatcaataatggcgggcatctgcagtcaccaaatgacctttgatacattccaaaacaaagccaatgtttgttgggctaagagcttggtccctatcctcgaaacagcggggataaaactaaatgataggcagtggtcccagataattcaagccttcaaagaagataaagcatactcacctgaagtagccctgaatgaaatatgcacgcgcatgtatggggtggatttagacagtgggctattttctaaaccattggtgtctgtgtactactcggataaccactgggataataggcctggagggaagatgttcggattcaaccccgaggcagcatccattctagaaagaaagtatccattcacaaaagggaagtggaacatcaacaagcagatctgngtgaccaccaggaggatagaagactttaaccctaccaccaatattataccggccaataggagactaccacactcattagtggccgaacaccgcccagtaaaaggggaaagaatggaatggctggttaataagataaacggccaccacgtgctcctggtcagtggctataatcttgtactgcctactaagagagtcacttgggtagcgccactaggtgtccgcggagcggactacacatacaacctagagttgggtctgccagcaacgcttggcaggtatgacctagtggtcataaacatccacacaccttttcgcatacaccattaccaacagtgcgtagaccacgcaatgaaactgcaaatgctcgggggtgactcattgagactgctcaaaccgggtggctctctattgatcagagcatatggttacgcagatagaaccagtgaacgagtcatctgcgtgttgggacgcaagttcagatcgtctagagcgttgaaaccaccatgtgtcaccagcaacactgagatgtttttcctattcagcaactttgacaatggcagaaggaatttcacaactcatgtcatgaataatcaactgaatgcagcctttgtaggacaggccacccgagcaggatgtgcaccgtcgtaccgggtaaaacgcatggacatcgcgaagaacgatgaagagtgcgtagttaacgccgctaaccctcgcgggttaccgggtgacggtgtttgcaaggcagtatacaaaaaatggccggagtcctttaagaatagtgcaacaccagtgggaaccgcaaaaacagtcatgtgcggtacgtatccagtaatccacgccgttggaccgaatttctctaattatttggaatctgaaggggaccgggaattggcagctgcctatcgagaagtcgcaaaggaagtaactagactgggagtaaatagtgtagctatacctctcctctccacaggtgtnnnnnnnnnnnnnnnnnnnnnnnnnnnnnnnnnnnnnnnnnnnnnnntcacagccatggactcgacggatgcagacgtggtcatctactgtcgcgacaaagaatgggagaagaaaatatccgaggccatacagatgcggactcaagtggagttgctggatgagcacatctccatagactgcgatattgttcgcgtgcaccctgacagcagcttggcaggcagaaaaggatacagcaccacggaaggcgcactgtactcatatctagaagggactcgttttcaccagacggcagtggacatggcagagatatatactatgtggccaaagcaaacagaggccaacgagcaagtctgcctatatgccctgggggaaagtattgaatcgatcaggcagaaatgcccggtggatgatgcagacgcatcgtctcctccaaaaactgtcccgtgcctttgccgttacgccatgactccggaacgcgtcaccngacttcgcatgaaccacgtcacaagtataattgtgtgttcctcgtttccccttccaaagtacaaaatagaaggagtgcaaaaagtcaaatgctctaaggtaatgctatttgaccacaacgtgccatcacgcgtaagtccaagggaatatagatcttccccggaggctgcacaggaagcgagtacaaccacgtcactgacgcatagccaattcgacctaagcgttgatggcgagatactacccgtcccgtcagatctggatgctgacgccccagccctagaaccagctcnnnnnnnnnnnnnnnnnnnnnnnnnnnnnnnnnnnnnnnnnnnnnnnnnnnnnnnnnnnnnnnnnnnnnnnnnnnnnnnnnnnnnnnnnnnnnnnnnnnnnnnnnnnnnnnnnnnnnnnnnnnnnnnnnnnnnnnnnnnnnnnnnnnnnnnnnnnnnnnnnnnnnnnnnnnnnnnnnnnnnnnnnnnnnnnnnnnnnnnnnnnnnnnnnnnnnnnnnnnnnnnnnnnnnnnnnnnnnnnnnncaggcaccgccgagtaccgccacggaactgaatcatccgccgatctccttnggngcaccaagnnnnnnnnnnnnnnnnnnnnnnnnnnnnnnnnnnnnnnnnnnnnnnnnnnnnnnnnnnnnnnnnnnnnnnnnnnnnnnnnnnnnnnnnnnnnnnnnnnnnnnnnnnnnnnnnnnnnnnnnnnnnnnnnnnnnnnnnnnnnnnnnnnnnnnnnn-nnagacagggcaggtgggtatatattctcgtcggacaccggtccaggtcatttacaacagaagtcagtacgccagtcagtgctgccggtgaacaccctggaggaagtccacgaggagaagtgttacccacctaagttggatgaagtaaaggagcaactattacttaagaaactccaggagagtgcatccatggccaacagaagcaggtaccagtcgcgcaaagtagaaaacatgaaagcaacaatcatccagagactaaagagaggctgtagactatacttaatgtcagagacctcaaaagtccctacttaccggaccacatatccggcgcctgtgtactcgcctccggtcaacgtccgactgtccaaccccgagtccgcagtggcagcatgcaatgagttcctagctagaaactatccaactgtctcatcataccaaattaccgacgagtatgatgcatatttagacatggtggacgggtcggagagttgcctggatcgagcgacattcaatccgtcaaaacttaggagctacccgaaacagcacgcttaccatgcgccctccatcagaagcgctgtaccgtccccattccagaacacactacagaatgtactggcagcagccacgaaaagaaactgcaacgtcacacagatgagggaattacccactttggactcagcagtattcaacgtggagtgttttaaaaaattcgcatgcaaccaagaatactgggaagaatttgctgccagccctattaggataacaactgagaatttaacaacctatgttactaaactaaaagggccaaaagcagcagcgctatttgcnnnnnnnnnnnnnctgctgccactgcaggaagtaccaatggataggttcacagtagatatgaaaagggacgtgaaggtgactcctggtacaaagcacacagaggaaagacctaaggtgcaggttatacaggcggctgaacccttggcgacagcatacctatgtgggattcacagagagctggttaggaggctgaacgccgtcctcctacccaatgtacatacactatttgatatgtctgccgaggatttcgatgccatcatagccgcacactttaagccaggagacactgttttagaaacggacatagcctcctttgacaagagccaagatgattcacttgcgcttactgccttgatgttgttagaagatttaggggtggatcactccctgttggacttgatagaggccgcttttggagagatttccagctgtcatctaccgacaggtacgcgcttcaagttcggcgccatgatgaaatctggtatgttcctaactctgttcgtcaacacattgctaaacatcaccatcgctagccgcgtgctggaagatcgtctgacaaaatccgcgtgcgcggccttcatcggcgacgacaacataatacatggagtcgtctccgatgaattgatggcagacagatgcgccacttggatgaacatggaagtgaagatcatagatgcagttgtatccttgaaagccccttacttttgtggagggtttatactgcacgatactgtgacgggaacagcctgcagagtggcagacccgctaaaaaggctattcaaactgggcaaaccgctagcggcaggtgacgaacaagatgaagatagaagacgagcgctggctgacgaagtgatcagatggcaacgaacagggctaattgatgagttggagaaagcggtatactctaggtatgaagtgcagggtatatcagttgtggtaatgtccatggccacctttgcaagctccagatctaacttcgagaagctcagaggacccnnnnnnnnnnnnnnnnnnnnnnnnnnnnnnnnnnnnnnnnnnnnnnnnnnnnnnnnnnnnnnnnnnnnnnnnnnnnnnnnnnnnnnnnnnnnnnnnnnnnnnnnnnnnnnnnnnnnnnnnnnnnnnnnnnnnnnnnnnnnnnnnnnnnnnnnnnnnnnnnnnnnnnnnnnnnnnnnnnnnnnnnnnnnnnnnnnnnnnnnnnnnnnnnnnnnnnnnnnnnnnnnnnnnnnnnnnnnnnnnnnnnnttggtgggggacaaagtaatgaaaccagcacacgtaaaggggaccatcgataacgcggacctggccaaactggcctttaagcggtcatctaagtatgaccttgaatgcgcgcagatacccgtgcacatgaagtccgacgcttcgaagttcacccatgagaaaccagaggggtactacaactggcaccacggagcagtacagtactcaggaggccggttcaccatccccacaggtgctggcaaaccaggggacagcggcagaccgatcttcgacaacaagggacgcgtggtggccatagtcttaggaggagctaatgaaggagcccgtacagccctctcggtggtgacctggaataaagacattgtcactaaaatcacccccgagggggccgaagagtggagtcttgccatcccagtcatgtgcctgctggcaaataccacgttcccctgctcccagcccccttgcactccctgctgctatgaaagggaaccagaggaaaccctacgcatgcttgaggacaacgtcatgagacctgggtactatcagctgctacaagcattaaagacggatgatagccacgattggaccaagctgcgttacatggacaaccacacgccagcggacgcagagagggcggggctatttgtaagaacatcagcaccgtgcacgattactggaacaatgggacacttcatcctgacccgatgtccgaaaggggaaactctgacggtgggattcactgacagtaggaagatcagtcactcatgtacgcacccatttcaccacgaccctcctgtgataggccgggagaaattccattcccgaccgcagcacggtaaagagctgccttgcagcacgtacgtgcagagcaccgccgcaactaccgaggagatagaggtacacatgcccccagacacccctgatcgcacactgatgtcacaacagtccggcaacgtaaagatcacagttaatggccagacggtgcggtacaagtgtaattgcggtggctcaaatgaaggactaataactacagacaaagtgattaataactgcaaagttgatcaatgtcatgccgcggtcaccaatcacaaaaagtggcagtacaactcccctctggtcccgcgtaatgctgaacttggggaccgaaaaggaaaaatccacatcccgtttccgctggcaaatgtaacatgcagggtgcctaaagcaaggaaccccaccgtgacgtacgggaaaaaccaagtcatcatgctactgtatcccgaccacccaacactcctgtcctaccggaacatgggagaagaaccaaactaccaagaagagtgggtgacgcataagaaggaagtcgtgctaaccgtgccgactgaagggctcgaggtcacgtggggtaacaacgagccgtataagtattggccgcagttatctacaaacggtacagcccatggccacccgcatgagataattctgtattattatgagctgtaccctactatgactgtagtagttgtgtcagtggcctcgttcgtactcctgtcgatggtgggtgtggcagtggggatgtgcatgtgtgcacgacgcagatgcannnnnnnnnncgaactgacaccaggagctaccgtccctttcctgcttagcctaatatgctgcatcagaacagctaaagcggccacataccaagaggctgcggtatacctgtggaatgaacagcaacctttgttttggctacaagcccttattccgctggcagccctgattgtcctgtgcaactgtctgagactcttaccatgctgctgtaaannnnnnnnnnnnnnnnnnnnnnnnnnnnnnnnnnnncacactgtgagcgcgtacgaacacgtaacagtgatcccgaacacggtgagagtaccgtataagactctagtcaatagaccgggctacagtcccatggtattggagatggaactactgtcagtcactttggagccaacgctatcgcttgattacatcacgtgcgagtacaaaaccgttatcccgtctccgtacgtgaaatgctgcggtacagcagagtgcaaggacaaaaacctacctgactacagctgtaaggtcttcaccggcgtctacccatttatgtggggcggagcctgccaaattcattgtggggccaatgtcttcagcctggacacctttcgacaacaaaattgtggtgtacaaaggtgacgtctataacatggactacccgccctttggcgcaggaagaccaggacaatttggcgatatccaaagtcgcacacctgagagtacagacgtctatgctaatacacaactggtactgcagagaccggctgcgggtacggtacatgtgccatactctcaggcaccatctggctttaagtattggctaaaagaacgaggggcgtcgctgcagcacacagcaccatttggctgccaaatagcaacaaacccggtaagagcggtgaattgcgccgtagggaacatgcccatctccatcgacataccgga

>MK156055

cgtctgcccgatgcgcagcgcagaagatcccgagagactcgccaattatgcgagaaagctagcatctgctgcaggaaaagtcctggacagaaacatctctggaaagatcggggacttacaagcagtaatggccgtgccagacacggagacgccaacattctgcttgcacacagatgtctcatgtagacagagagcagacgtcgcgatataccaagacgtctatgctgtacacgcacccacgtcgctataccaccaggcgattaaaggggtccgagtggcgtactgggtagggttcgacacaaccccgttcatgtacaatgccatggcgggtgcctacccctcatattcgacaaactgggcagacgagcaggtactgaaggctaagaacataggattatgttcaacagacctgacggaaggtagacgaggtaaattgtctattatgagagggaagaagctaaaaccgtgtgaccgtgtgctgttctcagtagggtcaacgctctacccggaaagccgcaagctacttaagagctggcacttaccatcggtgttccatctaaagggcaaactcagcttcacatgccgctgtgatacagtggtttcgtgcgagggctacgtcgttaagagaataacgatgagcccaggcctttatggaaaaaccacagggtatgcggtaacccaccacgcagacggattcttgatgtgcaagactaccgacacggttgacggcgaaagagtgtcattctcggtgtgcacatacgtgccggcgaccatttgtgatcaaatgaccggcatccttgctacagaagtcacgccggaggatgcacagaagctgttggtggggctgaaccagagaatagtggttaacggcagaacgcaacggaatacgaacaccatgaaaaactatctgcttcccgtggtcgcccaagccttcagtaagtgggcaaaggagtgccggaaagacatggaagatgaaaaactcctgggggtcagagaaagaacactgacctgctgctgtctatgggcattcaagaagcagaaaacacacacggtctacaagaggcctgatacccagtcaattcagaaggttcaggccgagtttgacagctttgtggtaccgagcctgtggtcgtccgggttgtcaatccccttgaggactagaatcaaatggttgttaagcaaggtgccaaaaaccgacctgatcccatatagcggggacgcccaagaagcccgggacgcagaaaaagaagcagaggaagaacgagaagcannnnnnnnnnnnnnnnnnnnnnnnnnnnnnnnnnnnnnnnnnnnnnnnnntcaggtcgaaatcgacgtggaacagcttgaggacagagcgggtgcaggaataatagagactcctagaggagctatcaaagttactgcccaaccaacagaccacgtcgtgggagagtacctggtactttccccgcagaccgtactacggagccagaagctcagcctaattcacgctttggcggagcaagtgaagacgtgcanncacagcggacgagcagggaggtacgcggtcgaagcgtacgacggccgagtcctagtgccctcaggctatgcaatctcgcctgaagacttccagagtctaagcgaaagcgcaacgatggtgtataacgaaagagagttcgtaaacagaaagctacaccacattgcgatgcacggaccagccctgaacaccgacgaagagtcgtatgagctggtgagggcagagaggacagaacacgagtacgtctacgatgtggaccagagaagatgctgtaagaaggaagaagctgcaggactggtattggtgggcgacttgactaatccgccctaccacgaattcgcatatgaagggctaaaaatccgccctgcctgcccatacaaaattgcagtcataggagtcttcggagtaccaggatctggcaagtcagctattatcaagaacctagttaccaggcaagacctggtgactagcggaaagaaagaaaactgccaagaaatcaccaccgacgtgatgagacagagaggtctagagatatctgcacgtacggttgactcgctgctcttgaatggatgcaacaggccagtcgacgtgttgtacgtagacgaggcgtttgcgtgccactctggaannnnnnnnnnnnnnnnnnnnnnnnnnnnnnnnnnnnnnnnnnnnnnnnnnnnnnnnnnnnnnnnnnnnnnnnnnnnnnnnnnnnnnnnnnnnnnnnnnnnnnnnnnnnnnnnnnnnnnnnnnnnnnnnnnnnnnnnnnnnnnnnnnnnnnnnnnnnnnnnnnnnnnnnnnnnnnnnnnnnnnnnnnnnnnnnnnnnnnnnnnnnnnnnnnnnnnnnnnnnnnnnnnnnnnnnnnnnnnnnnnnnnnnnnnnnnnnnnnnnnnnnnnnnnnnnnnnnnnnnnnnnnnnnnnnnnnnnnnnnnnnnnnnnnnnnnnnnnnnnnnnnnnnnnnnnnnnnnnnnnnnnnnnnnnnnnnnnnnnnnnnnnnnnnnnnnnnnnnnnnnnnnnnnnnnnnnnnnnnnnnnnnnnnnnnnnnnnnnnnnnnnnnnnnnnnnnnnnnnnnnnnnnnnnnnnnnnctcctaacncgtacggaaggtaaactggtatggaagacactttccggtgacccgtggataaagacgctgcagaacccaccgaaaggaaacttcaaagcaactatcaaggagtgggaggtggagcatgcatcaataatggcgggcatctgcagtcaccaaatgacctttgatacattccaaaacaaagccaatgtttgttgggctaagagcttggtccctatcctcgaaacagcggggataaaactaaatgataggcagtggtcccagataattcaagccttcaaagaagataaagcatactcacctgaagtagccctgaatgaaatatgcacgcgcatgtatggggtggatttagacagtgggctattttctaaaccattggtgtctgtgtactactcggataaccactgggataataggcctggagggaagatgttcggattcaaccccgaggcagcatccattctagaaagaaagtatccattcacaaaagggaagtggaacatcaacaagcagatctgngtgaccaccaggaggatagaagactttaaccctaccaccaatattataccggccaataggagactaccacactcattagtggccgaacaccgcccagtaaaaggggaaagaatggaatggctggttaataagataaacggccaccacgtgctcctggtcagtggctataatcttgtactgcctactaagagagtcacttgggtagcgccactaggtgtccgcggagcggactacacatacaacctagagttgggtctgccagcaacgcttggcaggtatgacctagtggtcataaacatccacacaccttttcgcatacaccattaccaacagtgcgtagaccacgcaatgaaactgcaaatgctcgggggtgactcattgagactgctcaaaccgggtggctctctattgatcagagcatatggttacgcagatagaaccagtgaacgagtcatctgcgtgttgggacgcaagttcagatcgtctagagcgttgaaaccaccatgtgtcaccagcaacactgagatgtttttcctattcagcaactttgacaatggcagaaggaatttcacaactcatgtcatgaataatcaactgaatgcagcctttgtaggacaggccacccgagcaggatgtgcaccgtcgtaccgggtaaaacgcatggacatcgcgaagaacgatnnagngtgcnnnnnnnnnnnnnnnnnnnnnnnnnnnnnnnnnnnnnnnnnnnnnnnnnnnnnnnnnnnnnnnnnnnnnnnnnnnnnnnnnnnnnnnnnnnnnnnnnnnnnnnnnnnnnnnnnnnnnnnnnnnnnnnnnnnnnnnnnnnnnnnnnnnnnnnnnnnnnnnnnnnnnnnnnnnnnnnnnnnnnnnnnnnnnnnnnnnnnnnnnnnnnnnnnnnnnnnnnnnnnnnnnnnnnnnnnnnnnnnnnnnnnnnnnnnnnnnnnnnnnnnnnnnnnnnnnnnnnnnnnnnnnnnnnnnnnnnnnnnnnnnnnnnnnnnnnnnnnnnnnnnnnnnnnnnnnnnnnnnnnnnnnncgacggatgcagacgtggtcatctactgtcgcgacaaagaatgggagaagaaaatatccgaggccatacagatgcggactcaagtggagttgctggatgagcacatctccatagactgcgatattgttcgcgtgcaccctgacagcagcttggcaggcagaaaaggatacagcaccacggaaggnnnnnnnnnnnnnnnnnnnnnnnnnnnnnnnnnnnnnnnnnnggcagtggacatggcagagatatatactatgtggccaaagcaaacagaggccaacgagcaagtctgcctatatgccctgggggaaagtattgaatcgatcaggcagaaatgcccggtggatgatgcagacgcatcgtctcctccaaaaactgtcccgtgcctttgccgttacgccatgactccggaacgcgtcaccagacttcgcatgaaccacgtcacaagtataattgtgtgttcctcgtttccccttccaaagtacaaaatagaaggagtgcnaaaagtcaaatgctctaaggtaatgctatttgaccacaacgtgccatcacgcgtaagtccaagggaatatagatcttccccggaggctgcacaggaagcgagtacaaccacgtcactgacgcatagccaattcgacctaagcgttgatggcgagatactacccgtcccgtcagatctggatgctgacgccccagccctagaaccagctctagacgacagggcgacacacacgttgccatccacaaccggaaaccttgcggccgtgtctgactgggtaatgagcaccgtacctgtcgcgccgcccagaagaaggcgagggagaaacctgactgtgacatgcgacgagagagaagggaacataacacccatggctagcgtccgattctttagggcagagctgcgtccggtcgtacaagaaacagcggagacgcgtgacacagcaatgtctcttcaggcaccgccgagtaccgccacggaactgaatcatccgccgatctccttcggagcaccaagnnnnnnnnnnnnnnnnnnnnnnnnnnnnnnnnnnnnnnnnnnnnnnnnnnnnnnnnnnnnnnnnnnnnnnnnnnnnnnnnnnnnnnnnnnnnnnnnnnnnnnnnnnnnnnnnnnnnnnnnnnnnnnnnnnnnnnnnnnnnnnnnnnnnnnnnnnnnagacagggcaggtgggtatatattctcgtcggacaccggtccaggtcatttacaacagaagtcagtacgccagtcagtgctgccggtgaacaccctggaggaagtccacgaggagaagtgttacccacctaagttggatgaagtaaaggagcaactattacttaagaaactccaggagagtgcatccatggccaacagaagcaggtaccagtcgcgcaaagtagaaaacatgaaagcaacaatcatccagagactaaagagaggctgtagactatacttaatgtcagagacctcaaaagtccctacttaccggaccacatatccggcgcctgtgtactcgcctccggtcaacgtccgactgtccaaccccgagtccgcagtggcagcatgcaatgagttcctagctagaaactatccaactgtctcatcataccaaattaccgacgagtatgatgcatatttagacatggtggacgggtcggagagttgcctggatcgagcgacattcaatccgtcaaaacttaggagctacccgaaacagcacgcttaccatgcgccctccatcagaagcgctgtaccgtccccattccagaacacactacagaatgtactggcagcagccacgaaaagaaactgcaacgtcacacagatgagggaattacccactttggactcagcagtattcaacgtggagtgttttaaaaaattcgcatgcaaccaagaatactgggaagaatttgctgccagccctattaggataacaactgagaatttaacaacctatgttactaaactaaaagggccaaaagcagcagcgctatttgcnnnnnnnnnnnnnnnnnnnnnnntgcaggaagtaccaatggataggttcacagtagatatgaaaagggacgtgaaggtgactcctggtacaaagcacacagaggaaagacctaaggtgcaggttatacaggcggctgaacccttggcgacagcatacctatgtgggattcacagagngctggttangaggctgaacgccgtcctccnacccaatgtacatacactatttgatatgtctgccgaggatttcgatgccatcatagccgcacactttaagccaggagacactgttttagaaacggacatagcctcctttgacaagagccaagatgattcacttgcgcttactgccttgatgttgttagaagatttaggggtggatcactccctgttggacttgatagaggccgcttttggagagatttccagctgtcannnnnnnnnnnnnnnnnnnnnnnnnnnnggcgccatgatgaaatctggtatgttcctaactctgttcgtcaacacattgctaaacatcaccatcgctagccgcgtgctggaagatcgtctgacaaaatccgcgtgcgcggccttcatcggcgacgacaacataatacatggagtcgtctccgatgaattgatggcagacagatgcgccacttggatgaacatggaagtgaagatcatagatgcagttgtatccttgaaagccccttacttttgtggagggtttatactgcacgatactgtgacgggaacagcctgcagagtggcagacccgctaaaaaggctattcaaactgggcaaaccgctagcggcaggtgacgaacaagatgaagatagaagacgagcgctggctgacgaagtgatcagatggcaacgaacagggctaattgatgagttggagaaagcggtatactctaggtatgaagtgcagggtatatcagttgtggtaatgtccatggccacctttgcaagctccagatctaacttcgagaagctcagaggacccnnnnnnnnnnnnnnnnnnnnnnnnnnnnnnnnnnnnnnnnnnnnnnnnnnnnnnnnnnnnnnnnnnnnnnnnnnnnnnnnnnnnnnnnnnnnnnnnnnnnnnnnnnnnnnnnnnnnnnnnnnnnnnnnnnnnnnnnnnnnnnnnnnnnnnnnnnnnnnnnnnnnnnnnnnnnnnnnnnnnnnnnnnnnnnnnnnnnnnnnnnnnnnnnnnnnnnnnnnnnnnnnnnnnnnnnnnnnnnnnnnnnnttggtgggggacaaagtaatgaaaccagcacacgtaaaggggaccatcgataacgcggacctggccaaactggcctttaagcggtcatctaagtatgaccttgaatgcgcgcagatacccgtgcacatgaagtccgacgcttcgaagttcacccatgagaaaccagaggggtactacaactggcaccacggagcagtacagtactcaggaggccggttcaccatccccacaggtgctggcaaaccaggggacagcggcagaccgatcttcgacaacaagggacgcgtggtggccatagtcttaggaggagctaatgaaggagcccgtacagccctctcggtggtgacctggaataaagacattgtcactaaaatcacccccgagggggccgaagagtggagtcttgccatcccagtcatgtgcctgctggcaaataccacgttcccctgctcccagcccccttgcactccctgctgctatgaaagggaaccagaggaaaccctacgcatgcttgaggacaacgtcatgagacctgggtactatcagctgctacaagcattaaagacggatgatagccacgattggaccaagctgcgttacatggacaaccacacgccagcggacgcagagagggcggggctatttgtaagaacatcagcaccgtgcacgattactggaacaatgggacacttcatcctgacccgatgtccgaaaggggaaactctgacggtgggattcactgacagtaggaagatcagtcactcatgtacgcacccatttcaccacgaccctcctgtgataggccgggagaaattccattcccgaccgcagcacnnnnnngagctgccttgcagcacgtacgtgcagagcaccgccgcaactaccgaggagatagaggtacacatgcccccagacacccctgatcgcacactgatgtcacaacagtccggcaacgtaaagatcacagttaatggccagacggtgcggtacaagtgtaattgcggtggctcaaatgaaggactaataactacagacaaagtgattaataactgcaaagttgatcaatgtcatgccgcggtcaccaatcacaaaaagtggcagtacaactcccctctggtcccgcgtaatgctgaacttggggaccgaaaaggaaaaatccacatcccgtttccgctggcaaatgtaacatgcagggtgcctaaagcaaggaaccccaccgtgacgtacgggaaaaaccaagtcatcatgctactgtatcccgaccacccaacactcctgtcctaccggaacatgggagaagaaccaaactaccaagaagagtgggtgacgcataagaaggaagtcgtgctaaccgtgccgactgaagggctcgaggtcacgtggggtaacaacgagccgtataagtattggccgcagttatctacaaacggtacagcccatggccacccgcatgagataattctgtattattatgagctgtaccctactatgactgtagtagttgtgtcagtggcctcgttcgtactcctgtcgatggtgggtgtggcagtggggatgtgcatgtgtgcacgacgcagatgcannnnnnnnnnngaactgacaccaggagctaccgtccctttcctgcttagcctaatatgctgcatcagaacagctaaagcggccacataccaagaggctgcggtatacctgtggaatgancagcaacctttgttttggctacaagcccttattccgctggcagccctgattgtccnatgcaacnnnnnnnnnnnnnnnnnnnnnnnnnnnnnnnnnnnnnnnnnnnnnnnnnnnnnnnnnnnnnnnnnnnnnnnnnnnagcgcgtacgaacacgtaacagtgatcccgaacacggtgagagtaccgtataagactctagtcaatagaccgggctacagtcccatggtattggagatggaactactgtcagtcactttggagccaacgctatcgcttgattacatcacgtgcgagtacaaaaccgttatcccgtctccgtacgtgaaatgctgcggtacagcagagtgcaaggacaaaaacctacctgactacagctgtaaggtcttcaccggcgtctacccatttatgtggggcggagcctgccaaattcattgtggggccaatgtcttcagcctggacacctttcgacaacaaaattgtggtgtacaaaggtgacgtctataacatggactacccgccctttggcgcaggaagaccaggacaatttggcgatatccaaagtcgcacacctgagagtacagacgtctatgctaatacacaactggtactgcagagaccggctgcgggtacggtacatgtgccatactctcaggcaccatctggctttaagtattggctaaaagaacgaggggcgtcgctgcagcacacagcaccatttggctgccaaatagcaacaaacccggtaagagcggtgaattgcgccgtagggaacatgcccatctccatcgacataccgga

>MK156056

cgtctgcccgatgcgcagcgcagaagatcccgagagactcgccaattatgcgagaaagctagcatctgctgcaggaaaagtcctggacagaaacatctctggaaagatcggggacttacaagcagtaatggccgtgccagacacggagacgccaacattctgcttgcacacagatgtctcatgtagacagagagcagacgtcgcgatataccaagacgtctatgctgtacacgcacccacgtcgctataccaccaggcgattaaaggggtccgagtggcgtactgggtagggttcgacacaaccccgttcatgtacaatgccatggcgggtgcctacccctcatattcgacaaactgggcagacgagcaggtactgaaggctaagaacataggattatgttcaacagacctgacggaaggtagacgaggtaaattgtctattatgagagggaagaagctaaaaccgtgtgaccgtgtgctgttctcagtagggtcaacgctctacccggaaagccgcaagctacttaagagctggcacttaccatcggtgttccatctaaagggcaaactcagcttcacatgccgctgtgatacagtggtttcgtgcgagggctacgtcgttaagagaataacgatgagcccaggcctttatggaaaaaccacagggtatgcggtaacccaccacgcagacggattcttgatgtgcaagactaccgacacggttgacggcgaaagagtgtcattctcggtgtgcacatacgtgccggcgaccatttgtgatcaaatgaccggcatccttgctacagaagtcacgccggaggatgcacagaagctgttggtggggctgaaccagagaatagtggttaacggcagaacgcaacggaatacgaacaccatgaaaaactatctgcttcccgtggtcgcccaagccttcagtaagtgggcaaaggagtgccggaaagacatggaagatgaaaaactcctgggggtcagagaaagaacactgacctgctgctgtctatgggcattcaagaagcagaaaacacacacggtctacaagaggcctgatacccagtcaattcagaaggttcaggccgagtttgacagctttgtggtaccgagcctgtggtcgtccgggttgtcaatccccttgaggactagaatcaaatggttgttaagcaaggtgccaaaaaccgacctgatcccatatagcggggacgcccaagaagcccgggacgcagaaaaagaagcagaggaagaacgagaagcannnnnnnnnnnnnnnnnnnnnnnnnnnnnnnnnnnnnnnnnnnnnnnnnnnnnnnnnnnnnnnnnnnnnnnnnnnnnnnnnnnnnnnnnnnnnnnnnnnnnnnnnnnnnnnnnnnnnnnnnnnnnnnnnnnnnnnnnnnnnnnnnnnnnnnnnnnnnnnnnnnnnnnnnnnnnnnnnnnnnnnnnnnnnnnnnnnnnnnnnnnnnnnnnnnnnnnnnnnnnnnnnnnnnnnnnnnnnnnnnnnnnnnnnnnncacagcggacgagcagggaggtacgcggtcgaagcgtacgacggccgagtcctagtgccctcaggctatgcaatctcgcctgaagacttccagagtctaagcgaaagcgcaacgatggtgtataacgaaagagagttcgtaaacagaaagctacaccacattgcgatgcacggaccagccctgaacaccgacgaagagtcgtatgagctggtgagggcagagaggacagaacacgagtacgtctacgatgtggaccagagaagatgctgtaagaaggaagaagctgcaggactggtattggtgggcgacttgactaatccgccctaccacgaattcgcatatgaagggctaaaaatccgccctgcctgcccatacaaaattgcagtcataggagtcttcggagtaccaggatctggcaagtcagctattatcaagaacctagttaccaggcaagacctggtgactagcggaaagaaagaaaactgccaagaaatcaccaccgacgtgatgagacagagaggtctagagatatctgcacgtacggttgactcgctgctcttgaatggatgcaacaggccagtcgacgtgttgtacgtagacgaggcgtttgngnnnnnnnnnnnnnnnnnnnnnnnnnnnnnnnnnnnnnnnnnnnnnnnnnnnnnnnnnnnnnnnnnnnnnnnnnnnnnnnnnnnnnnnnnnnnnnnnnnnnnnnnnnnnnnnnnnnnnnnnnnnnnnnnnnnnnnnnnnnnnnnnnnnnnnnnnnnnnnnnnnnnnnnnnnnnnnnnnnnnnnnnnnnnnnnnnnnnnnnnnnnnnnnnnnnnnnnnnnnnnnnnnnnnnnnnnnnnnnnnnnnnnnnnnnnnnnnnnnnnnnnnnnnnnnnnnnnnnnnnnnnnnnnnnnnnnnnnnnnnnnnnnnnnnnnnnnnnnnnnnnnnnnnnnnnnnnnnnnnnnnnnnnnnnnnnnnnnnnnnnnnnnnnnnnnnnnnnnnnnnnnnnnnnnnnnnnnnnnnnnnnnnnnnnnnnnnnnnnnnnnnnnnnnnnnnnnnnnnnnnnnnnnnnnnnnnnnnnnnnnnnnnnnnnnnnnnnnnnnnnnnnnnnnnnnnnnnnnnnnnnnnnnnnnnnnnnnnnnnnnnnnnnnnnnnnnnnnnnnnnnnnnnnnnnnnnnnnnnnnnnnnnnnnnnnnnnnnnnnnnnnnnnnnnnnnnnnnnnnnnnnnnnnnnnnnnnnnnnnnnnnnnnnnnnnnnnnnnnnnnnnnnnnnnnnnnnnnnnnnnnnnnnnnnnnnnnnnnnnnnnnnnnnnnnnnnnnnnnnnnnnggataaaactaaatgataggcagtggtcccagataattcaagccttcaaagaagataaagcatactcacctgaagtagccctgaatgaaatatgcacgcgcatgtatggggtggatttagacagtgggctattttctaaaccattggtgtctgtgtactactcggataaccactgggataataggcctggagggaagatgttcggattcaaccccgaggcagcatccattctagaaagaaagtatccattcacaaaagggaagtggaacatcaacaagnnnnnnnnnnnnnnnnnnnnnnnnnnnnnnnnnnnnnnnnnnnnnnnnnnnnnnnnnnnnnnnnnnnnnnnnnnnnnnnnnnnnnnnnnnnnnnnnnnnnnnnnnnnnnnnnnnnnnnnnnnnnnnnnnnnnnnnnnnnnnnnnnnnnnnnnnnnnnnnnnnnnnnnnnnnnnnnnnnnnnnnnnnnnnnnnnnnnnnnnnnnnnnnnnnnnnnnnnnnnnnnnnnnnnnnnnnnnnnnnnnnnnnnnnnnnnnnnnnnnnnnnnnnnnnnnnnnnnnnngnaggtatgacctagtggtcataaacatccacacaccttttcgcatacaccattaccaacagtgcgtagaccacgcaatgaaactgcaaatgctcgggggtgactcattgagactgctcaaaccgggtggctctctattgatcagagcatatggttacgcagatagaaccagtgaacgagtcatctgcgtgttgggacgcaagttcagatcgtctagagcgttgaaaccaccatgtgtcaccagcaacactgagatgtttttcctattcagcaactttgacaatggcagaaggaatttcacaactcatgtcatgaataatcaactgaatgcagcctttgtaggacaggccacccgagcaggatgtgcaccgtcgtaccgggtaaaacgcatggacatcgcgaagaacgatgnagngtgcnnnnnnnnnnnnnnnnnnnnnnnnnnnnnnnnnnnnnnnnnnnnnnnnnnnnnnnnnnnnnnnnnnnnnnnnnnnnnnnnnnnnnnnnnnnnnnnnnnnnnnnnnnnnnnnnnnnnnnnnnnnnnnnnnnnnnnnnnnnnnnnnnnnnnnnnnnnnnnnnnnnnnnnnnnnnnnnnnnnnnnnnnnnnnnnnnnnnnnnnnnnnnnnnnnnnnnnnnnnnnnnnnnnnnnnnnnnnnnnnnnnnnnnnnnnnnnnnnnnnnnnnnnnnnnnnnnnnnnnnnnnnnnnnnnnnnnnnnnnnnnnnnnnnnnnnnnnnnnnnnnnnnnnnnnnnnnnnnnnnnnnnnnnnnnnnnnnnnnnnnnnnnnnnnnnnnnnnnnnnnnnnnnnnnnnnnnnnnnnnnnnnnnnnnnnnnnnnnnnnnnnnnnnnnnnnnnnnnnnnnnnnnnnnnnnnnnnnnnnnnnnnnnnnnnnnnnnnnnnnnnnnnnnnnnnnnnnnnnnnnnnnnnnnnnnnnnnnnnnnnnnnnnnnnnnnnnnnnnnnnnnnnnnnnnnnnnnnnnnnnnnnnnnnnnnnnnnnnnnnnnnnnnnnnnnnnnnnnnnnnnnnnnnnnnnnnnnnnnnnnnnnnnnnnnnnnnnnnnnnnnnnnnnnnnnnnnnnnnnnnnnnnnnnnnnnnnnnnnnnnnnnnnnnnnnnnnnnnnnnnnnnnnnnnnnnnnnnnnnnnnnnnnnnnnnnnnnnnnnnnnnnnnnnnnnnnnnnnnnnnnnnnnnnnnnnnnnnnnnnnnnnnnnnnnnnnnnnnnnnnnnnnnnnnnnnnnnnnnnnnnnnnnnnnnnnnnnnnnnnnnnnnnnnnnnnnnnnnnnnnnnnnnnnnnnnnnnnnnnnnnnnnnnnnnnnnnnnnnnnnnnnnnnnnnnnnnnnnnnnnnnnnnnnnnnnnnnnnnnnnnnnnnnnnnnnnnnnnnnnnnnnnnnnnnnnnnnnnnnnnnnnnnnnnnnnnnnnnnnnnnnnnnnnnnnnnnnnnnnnnnnnnnnnnnnnnnnnnnnnnnnnnnnnnnnnnnnnnnnnnnnnnnnnnnnnnnnnnnnnnnnnnnnnnnnnnnnnnnnnnnnnnnnnnnnnnnnnnnnnnnnnnnnnnnnnnnnnnnnnnnnnnnnnnnnnnnnnnnnnnnnnnnnnnnnnnnnnnnnnnnnnnnnnnnnnnnnnnnnnnnnnnnnnnnnnnnnnnnnnnnnnnnnnnnnnnnnnnnnnnnnnnnnnnnnnnnnnnnnnnnnnnnnnnnnnnnnnnnnnnnnnnnnnnnnnnnnnnnnnnnnnnnnnnnnnnnnnnnnnnnnnnnnnnnnnnnnnnnnnnnnnnnnnnnnnnnnnnnnnnnnnnnnnnnnnnnnnnnnnnnnnnnnnnnnnnnnnnnnnnnnnnnnnnnnnnnnnnnnnnnnnnnnnnnnnnnnnnnnnnnnnnnnnnnnnnnnnnnnnnnnnnnnnnnnnnnnnnnnnnnnnnnnnnnnnnnnnnnnnnnnnnnnnnnnnnn-nnagacagggcaggtgggtatatattctcgtcggacaccggtccaggtcatttacaacagaagtcagtacgccagtcagtgctgccggtgaacaccctggaggaagtccacgaggagaagtgttacccacctaagttggatgaagtaaaggagcaactattacttaagaaactccaggagagtgcatccatggccaacagaagcaggtaccagtcgcgcaaagtagaaaacatgaaagcaacaatcatccagagactaaagagaggctgtagactatacttaatgtcagagacctcaaaagtccctacttaccggaccacatatccggcgcctgtgtactcgcctccggtcaacgtccgactgtccaaccccgagtccgcagtggcagcatgcaatgagttcctagctagaaactatccaactgtctcatcataccaaattaccgacgagtatgatgcatatttagacatggtggacgggtcggagagttgcctggatcgagcgacattcaatccgtcaaaacttaggagctacccgaaacagcacgcttaccatgcgccctccatcagaagcgctgtaccgtccccattccagaacacactacagaatgtactggcagcagccacgaaaagaaactgcaacgtcacacagatgagggaattacccactttggactcagcagtattcaacgtggagtgttttaaaaaattcgcatgcaaccaagaatactgggaagaatttgctgccagccctattaggataacaactgagaatttaacaacctatgttactaaactaaaagggccaaaagcagcagcgctatttgcnnnnnnnnnnnnnnnnnnnnnnntgcaggaagtaccaatggataggttcacagtagatatgaaaagggacgtgaaggtgactcctggtacaaagcacacagaggaaagacctaaggtgcaggttatacaggcggctgaacccttggcgacagcatacctatgtgggattcacagagagctggttaggaggctgaacgccgtcctcctacccaatgtacatacactatttgatatgtctgccgaggatttcgatgccatcatagccgcacactttaagccaggagacactgttttagaaacggacatagcctcctttgacaagagccaagatgattcacttgcgcttactgccttgatgttgttagaagatttaggggtggatcactccctgttggacttgatagaggccgcttttggagagatttccagctgtcannnnnnnnnnnnnnnnnnnnnnnnnnnnggcgccatgatgaaatctggtatgttcctaactctgttcgtcaacacattgctaaacatcaccatcgctagccgcgtgctggaagatcgtctgacaaaatccgcgtgcgcggccttcatcggcgacgacaacataatacatggagtcgtctccgatgaattgatggcagacagatgcgccacttggatgaacatggaagtgaagatcatagatgcagttgtatccttgaaagccccttacttttgtggagggtttatactgcacgatactgtgacgggaacagcctgcagagtggcagacccgctaaaaaggctattcaaactgggcaaaccgctagcggcaggtgacgaacaagatgaagatagaagacgagcgctggctgacgaagtgatcagatggcaacgaacagggctaattgatgagttggagaaagcggtatactctaggtatgaagtgcagggtatatcagttgtggtaatgtccatggccacctttgcaagctccagatctaacttcgagaagctcagaggacccnnnnnnnnnnnnnnnnnnnnnnnnnnnnnnnnnnnnnnnnnnnnnnnnnnnnnnnnnnnnnnnnnnnnnnnnnnnnnnnnnnnnnnnnnnnnnnnnnnnnnnnnnnnnnnnnnnnnnnnnnnnnnnnnnnnnnnnnnnnnnnnnnnnnnnnnnnnnnnnnnnnnnnnnnnnnnnnnnnnnnnnnnnnnnnnnnnnnnnnnnnnnnnnnnnnnnnnnnnnnnnnnnnnnnnnnnnnnnnnnnnnnnttggtgggggacaaagtaatgaaaccagcnnnnnnnnnnnnnnnnnnnnnnnnnnnnnnnnnnnnnnnnnnnnnnnnnnnnnnnnnnnnnnnnnnnnnnnnnnnnnnnnnnnnnnnnnnnnnnnnnnnnnnnnnnnnnnnnnnnnnnnnnnnnnnnnnnnnnnnnnnnnnnnnnnnnnnnnnnnnnnnnnnnnnnnnnnnnnnnnnnnnnnnnnnnnnnnnnnnnnnnnnnnnnnnnnnnnnnnnnnnnnnnnnnnnnnnnnnnnnnnnnnnnnnnnnnnnacgcgtggtggccatagtcttaggaggagctaatgaaggagcccgtacagccctctcggtggtgacctggaataaagacattgtcactaaaatcacccccgagggggccgaagagtggagtcttgccatcccagtcatgtgcctgctggcaaataccacgttcccctgctcccagcccccttgcactccctgctgctatgaaagggaaccagaggaaaccctacgcatgcttgaggacaacgtcatgagacctgggtactatcagctgctacaagcattaaagacggatgatagccacgattggaccaagctgcgttacatggacaaccacacgccagcggacgcagagagggcggggctatttgtaagaacatcagcaccgtgcacgattactggaacaatgggacacttcatcctgacccgatgtccgaaaggggaaactctgacggtgggattcactgacagtaggaagatcagtcactcatgtacgcacccatttcaccacgaccctcctgtgataggccgggagaaattccattcccgaccgcagcacggtaaagagctgccttgcagcacgtacgtgcagagcaccgccgcaactaccgaggagatagaggtacacatgcccccagacacccctgatcgcacactgatgtcacaacagtccggcaacgtaaagatcacagttaatggccagacggtgcggtacaagtgtaattgcggtggctcgaatgaaggactaataactacagacaaagtgattaataactgcaaagttgatcaatgtcatgccgcggtcaccaatcacaaaaagtggcagtacaactcccctctggtcccgcgtaatgctgaacttggggaccgaaaaggaaaaatccacatcccgtttccgctggcaaatgtaacatgcagggtgcctaaagcaaggaaccccaccgtgacgtacgggaaaaaccaagtcatcatgctactgtatcccgaccacccaacactcctgtcctaccggaacatgggagaagaaccaaactaccaagaagagtgggtgacgcataagaaggaagtcgtgctaaccgtgccgactgaagggctcgnnnnnnnnnnnnnnnnnnnnnnnnnnnnnnnnnnnnnnnnnnnnnnnnnnnnnnnnnnnnnnnnnnnnnnnnnnnnnnnnnnnnnnnnnnnnnnnnnnnnnnnnnnnnnnnnnnnnnnnnnnnnnnnnnnnnnnnnnnnnnnnnnnnnnnnnnnnnnnnnnnnnnnnnnnnnnnnnnnnnnnnnnnnnnnnnnnnnnnnnnnnnnnnnnnnnnnnnnnnnngaactgacaccaggagctaccgtccctttcctgcttagcctaatatgctgcatcagaacagctaaagcggccacataccaagaggctgcggtatacctgtggaatgancagcaacctttgttttggctacaagcccttattccgctggcngncctgattgtnnnnnnnnnnnnnnnnnnnnnnnnnnnnnnnnnnnnnnnnnnnnnnnnnnnnnnnnnnnnnnnnnnnnnnnnnnnnnanactgtgagcgcgtacgaacacgtaacagtgatcccgaacacggtgagagtaccgtataagactctagtcaatagaccgggctacagtcccatggtattggagatggaactactgtcagtcactttggagccaacgctatcgcttgattacatcacgtgcgagtacaaaaccgttatcccgtctccgtacgtgaaatgctgcggtacagcagagtgcaaggacaaaaacctacctgactacagctgtaaggtcttcaccggcgtctacccatttatgtggggcggagcctgccaaattcattgtggggccaatgtcttcagcctggacacctttcgacaacaaaattgtggtgtacaaaggtgacgtctataacatggactacccgccctttggcgcaggaagaccaggacaatttggcgatatccaaagtcgcacacctgagagtacagacgtctatgctaatacacaactggtactgcagagaccggctgcgggtacggtacatgtgccatactctcaggcaccatctggctttaagtattggctaaaagaacgaggggcgtcgctgcagcacacagcaccatttggctgccaaatagcaacaaacccggtaagagcggtgaattgcgccgtagggaacatgcccatctccatcgacataccgga

>MK156061

cgtctgcccgatgcgcagcgcagaagatcccgagagactcgccaattatgcgagaaagctagcatctgctgcaggaaaagtcctggacagaaacatctctggaaagatcggggacttacaagcagtaatggccgtgccagacacggagacgccaacattctgcttgcacacagatgtctcatgtagacagagagcagacgtcgcgatataccaagacgtctatgctgtacacgcacccacgtcgctataccaccaggcgattaaaggggtccgagtggcgtactgggtagggttcgacacaaccccgttcatgtacaatgccatggcgggtgcctacccctcatattcgacaaactgggcagacgagcaggtactgaaggctaagaacataggattatgttcaacagacctgacggaaggtagacgaggtaaattgtctattatgagagggaagaagctaaaaccgtgtgaccgtgtgctgttctcagtagggtcaacgctctacccggaaagccgcaagctacttaagagctggcacttaccatcggtgttccatctaaagggcaaactcagcttcacatgccgctgtgatacagtggtttcgtgcgagggctacgtcgttaagagaataacgatgagcccaggcctttatggaaaaaccacagggtatgcggtaacccaccacgcagacggattcttgatgtgcaagactaccgacacggttgacggcgaaagagtgtcattctcggtgtgcacatacgtgccggcgaccatttgtgatcaaatgaccggcatccttgctacagaagtcacgccggaggatgcacagaagctgttggtggggctgaaccagagaatagtggttaacggcagaacgcaacggaatacgaacaccatgaaaaactatctgcttcccgtggtcgcccaagccttcagtaagtgggcaaaggagtgccggaaagacatggaagatgaaaaactcctgggggtcagagaaagaacactgacctgctgctgtctatgggcattcaagaagcagaaaacacacacggtctacaagaggcctgatacccagtcaattcagaaggttcaggccgagtttgacagctttgtggtaccgagcctgtggtcgtccgggttgtcaatccccttgaggactagaatcaaatggttgttaagcaaggtgccaaaaaccgacctgatcccatatagcggggacgcccaagaagcccgggacgcagaaaaagaagcagaggaagaacgagaagcagaactgactcgtgaagccctaccacctctacaggcagcacaggaagatgttcaggtcgaaatcgacgtggaacagcttgaggacagagcgggtgcaggaataatagagactcctagaggagctatcaaagttactgcccaaccaacagaccacgtcgtgggagagtacctggtactttccccgcagaccgtactacggagccagaagctcagcctaattcacgctttggcggagcaagtgaagacgtgcacgcacagcggacgagcagggaggtacgcggtcgaagcgtacgacggccgagtcctagtgccctcaggctatgcaatctcgcctgaagacttccagagtctaagcgaaagcgcaacgatggtgtataacgaaagagagttcgtaaacagaaagctacaccacattgcgatgcacggaccagccctgaacaccgacgaagagtcgtatgagctggtgagggcagagaggacagaacacgagtacgtctacgatgtggaccagagaagatgctgtaagaaggaagaagctgcaggactggtattggtgggcgacttgactaatccgccctaccacgaattcgcatatgaagggctaaaaatccgccctgcctgcccatacaaaattgcagtcataggagtcttcggagtaccaggatctggcaagtcagctattatcaagaacctagttaccaggcaagacctggtgactagcggaaagaaagaaaactgccaagaaatcaccaccgacgtgatgagacagagaggtctagagatatctgcacgtacggttgactcgctgctcttgaatggatgcaacaggccagtcgacgtgttgtacgtagacgaggcgtttgcgtgccactctggaacgttacttgctttgatcgccttggttagaccaaggcagaaagttgtactctgtggtgacccgaagcagtgcggcttcttcaatatgatgcagatgaaagtcaactataatcacaacatctgcacccaagtgtaccacaaaagtatctccaggcggtgtacactgcctgtgaccgccattgtgtcatcgttacattacgaaggcaaaatgcgcactacgaatgagtacaacaagccgattgtagtggacactacaggctcaacaaaacctgacgctggagatctcgtgttaacgtgcttcagagggtgggttaaacaactgcaaattgactatcgtggatacgaggtcatgacagcagccgcatcccaagggttaaccagaaaaggagtttatgcagttaggcaaaaagttaatgaaaacccgctctatgcatcaacgtcagagcacgtcaacgtactcctaacgcgtacggaaggtaaactggtatggaagacactttccggtgacccgtggataaagacgctgcagaacccaccgaaaggaaacttcaaagcaactatcaaggagtgggaggtggagcatgcatcaataatggcgggcatctgcagtcaccaaatgacctttgatacattccaaaacaaagccaatgtttgttgggctaagagcttggtccctatcctcgaaacagcggggataaaactaaatgataggcagtggtcccagataattcaagccttcaaagaagataaagcatactcacctgaagtagccctgaatgaaatatgcacgcgcatgtatggggtggatctagacagtgggctattttctaaaccattggtgtctgtgtactactcggataaccactgggataataggcctggagggaagatgttcggattcaaccccgaggcagcatccattctagaaagaaagtatccattcacaaaagggaagtggaacatcaacaagcagatctgtgtgaccaccaggaggatagaagacttcaaccctaccaccaatattataccggccaataggagactaccacactcattagtggccgaacaccgcccagtaaaaggggaaagaatggaatggctggttaataagataaacggccaccacgtgctcctggtcagtggctataatcttgtactgcctactaagagagtcacttgggtagcgccactaggtgtccgcggagcggactacacatacaacctagagttgggtctgccagcaacgcttggcaggtatgacctagtggtcataaacatccacacaccttttcgcatacaccattaccaacagtgcgtagaccacgcaatgaaactgcaaatgctcgggggtgactcattgagactgctcaaaccgggtggctctctattgannnnnnnnnnnnnnnnnnnnnntagaaccagtgaacgagtcatctgcgtgttgggacgcaagttcagatcgtctagagcgttgaaaccaccatgtgtcaccagcaacactgagatgtttttcctattcagcaactttgacaatggcagaaggaatttcacaactcatgtcatgaataatcaactgaatgcagcctttgtaggacaggccacccgagcaggatgtgcaccgtcgtaccgggtaaaacgcatggacatcgcgaagaacgatgaagagtgcgtagttaacgccgctaaccctcgcgggttaccgggtgacggtgtttgcaaggcagtatacaaaaaatggccggagtcctttaagaatagtgcaacaccagtgggaaccgcaaaaacagtcatgtgcggtacgtatccagtaatccacgccgttggaccgaatttctctaattatttggaatctgaaggggaccgggaattggcagctgcctatcgagaagtcgcaaaggaagtaactagactgggagtaaatagtgtagctatacctctcctctccacaggtgtnnnnnnnnnnnnnnnnnnnnnnnnnnnnnnnnnnnnnnnnnnnnnnnnnnnnnnnnnnnnnnnnnnnnnnnnnnnnnnnnnnnnnnnnnnnnnnnnnnnnnnnnnnnnnnnnnnnnnnnnnnnnnnnnnnnnnnnnnnnnnnnnnnnnnnnnnnnnnnnnnnnnnnnnnnccnnngnctnngatattgttnnnnnnnaccctgacagcngcnnngcaggcagnaaangatnnnnnnnnnnnnnnnnnnnnnnnnnnnnnnnnnnnnnnnnnnnnnnnnnnnnnnnnnnggcagtggacatggcagagatatatactatgtggccaaagcaaacagaggccaacgagcaagtctgcctatatgccctgggggaaagtattgaatcgatcaggcagaaatgcccggtggatgatgcagacgcatcgtctcctccaaaaactgtcccgtgcctttgccgttacgccatgactccggaacgcgtcaccagacttcgcatgaaccacgtcacaagtataattgtgtgttcctcgtttccccttccaaagtacaaaatagaaggagtgcaaaaagtcaaatgctctaaggtaatgctatttgaccacaacgtgccatcacgcgtaagtccaagggaatatagatcttccccggaggctgcacaggaagcgagtacaaccacgtcactgacgcatagccaattcgacctaagcgttgatggcgagatactacccgtcccgtcagatctggatgctgacgccccagccctagaaccagctctagacgacagggcgacacacacgttgccatccacaaccggaaaccttgcggccgtgtctgactgggtaatgagcaccgtacctgtcgcgccgcccagaagaaggcgagggagaaacctgactgtgacatgcgacgagagagaagggaacataacacccatggctagcgtccgattctttagggcagagctgcgtccggtcgtacaagaaacagcggagacgcgtgacacagcaatgtctcttcaggcaccgccgagtaccgccacggaactgaatcatccgccgatctccttcggagcaccaagcgagacgttccccattacatttggggacttcaacgaaggagaaatcgaaagcttgtcttctgagctgctaactttcggagacttcctacccggagaagtggatgacttgacagatagcgactggtccacgtgctcagacacggacgacgagttactagacagggcaggtgggtatatattctcgtcggacaccggtccaggtcatttacaacagaagtcagtacgccagtcagtgctgccggtgaacaccctggaggaagtccacgaggagaagtgttacccacctaagttggatgaagtaaaggagcaactattacttaagaaactccaggagagtgcatccatggccaacagaagcaggtaccagtcgcgcaaagtagaaaacatgaaagcaacaatcatccagagactaaagagaggctgtagactatacttaatgtcagagacctcaaaagtccctacttaccggaccacatatccggcgcctgtgtactcgcctccggtcaacgtccgactgtccaaccccgagtccgcagtggcagcatgcaatgagttcctagctagaaactatccaactgtctcatcataccaaattaccgacgagtatgatgcatatttagacatggtggacgggtcggagagttgcctggatcgagcgacattcaatccgtcaaaacttaggagctacccgaaacagcacgcttaccatgcgccctccatcagaagcgctgtaccgtccccattccagaacacactacagaatgtactggcagcagccacgaaaagaaactgcaacgtcacacagatgagggaattacccactttggactcagcagtattcaacgtggagtgttttaaaaaattcgcatgcaaccaagaatactgggaagaatttgctgccagccctattaggataacaactgagaatttaacaacctatgttactaaactaaaagggccaaaagcagcagcgctatttgcnnnnnnnnnnnnnnnnctgccactgcaggaagtaccaatggataggttcacagtagatatgaaaagggacgtgaaggtgactcctggtacaaagcacacagaggaaagacctaaggtgcaggttatacaggcggctgaacccttggcgacagcatacctatgtgggattcacagagagctggttaggaggctgaacgccgtcctcctacccaatgtacatacactatttgatatgtctgccgaggatttcgatgccatcatagccgcacactttaagccaggagacactgttttagaaacggacatagcctcctttgacaagagccaagatgattcacttgcgcttactgccttgatgttgttagaagatttaggggtggatcactccctgttggacttgatagaggccgcttttggagagatttccagctgtcannnnnnnnnnnnnnnnnnnnnnnnnnnnggcgccatgatgaaatctggtatgttcctaactctgttcgtcaacacattgctaaacatcaccatcgctagccgcgtgctggaagatcgtctgacaaaatccgcgtgcgcggccttcatcggcgacgacaacataatacatggagtcgtctccgatgaattgatggcagacagatgcgccacttggatgaacatggaagtgaagatcatagatgcagttgtatccttgaaagccccttacttttgtggagggtttatactgcacgatactgtgacgggaacagcctgcagagtggcagacccgctaaaaaggctattcaaactgggcaaaccgctagcggcaggtgacgaacaagatgaagatagaagacgagcgctggctgacgaagtgatcagatggcaacgaacagggctaattgatgagttggagaaagcggtatactctaggtatgaagtgcagggtatatcagttgtggtaatgtccatggccacctttgcaagctccagatctaacttcgagaagctcagaggaccccagttaataaactgacaatgcgcgcggtaccccaacagaagccacgcaggaatcggaagaataagaagcaaaagcaaaagcgacaggcgccacaaaacaacacaaatcaaaagaagcagccgcctaaaaagaaaccagctcaaaagaaaaagaagccgggccgcagagagaggatgtgcatgaaaatcgaaaatgattgtatttttgaagtcaagtatgaaggtaaggtaacaggttacgcgtgcttggtgggggacaaagtaatgaaaccagcacacgtaaaggggaccatcgataacgcggacctggccaaactggcctttaagcggtcatctaagtatgaccttgaatgcgcgcagatacccgtgcacatgaagtccgacgcttcgaagttcacccatgagaaaccagaggggtactacaactggcaccacggagcagtacagtactcaggaggccggttcaccatccccacaggtgctggcaaaccaggggacagcggcagaccgatcttcgacaacaagggacgcgtggtggccatagtcttaggaggagctaatgaaggagcccgtacagccctctcggtggtgacctggaataaagacattgtcactaaaatcacccccgagggggccgaagagtggagtcttgccatcccagtcatgtgcctgctggcaaataccacgttcccctgctcccagcccccttgcactccctgctgctatgaaagggaaccagaggaaaccctacgcatgcttgaggacaacgtcatgagacctgggtactatcagctgctacaagcattaaagacggatgatagccacgattggaccaagctgcgttacatggacaaccacacgccagcggacgcagagagggcggggctatttgtaagaacatcagcaccgtgcacgattactggaacaatgggacacttcatcctgacccgatgtccgaaaggggaaactctgacggtgggattcactgacagtaggaagatcagtcactcatgtacgcacccatttcaccacgaccctcctgtgataggccgggagaaattccattcccgaccgcagcacnnnnnagagctgccttgcagcacgtacgtgcagagcaccgccgcaactaccgaggagatagaggtacacatgcccccagacacccctgatcgcacactgatgtcacaacagtccggcaacgtaaagatcacagttaatggccagacggtgcggtacaagtgtaattgcggtggctcaaatgaaggactaataactacagacaaagtgattaataactgcaaagttgatcaatgtcatgccgcggtcaccaatcacaaaaagtggcagtacaactcccctctggtcccgcgtaatgctgaacttggggaccgaaaaggaaaaatccacatcccgtttccgctggcaaatgtaacatgtagggtgcctaaagcaaggaaccccaccgtgacgtacgggaaaaaccaagtcatcatgctactgtatcccgaccacccaacactcctgtcctaccggaacatgggagaagaaccaaactaccaagaagagtgggtgacgcataagaaggaagtcgtgctaaccgtgccgactgaagggctcgaggtcacgtggggtaacaacgagccgtataagtattggccgcagttatctacaaacggtacagcccatggccacccgcatgagataattctgtattattatgagctgtaccctactatgactgtagtagttgtgtcagtggcctcgttcgtactcctgtcgatggtgggtgtggcagtggggatgtgcatgtgtgcacgacgcagatgcannnnnnnnnnnnnnnnnnnnnnnnnnnnnnnnnnnnnnnnnnnnnnnnnnnnnnnnnnnnnnnnnnnnnnnnnnnnnnnnnnnnnnnnnnnnnnnnnnnnnnnnnnnnnnnnnnnnnnnnnnnnnnnnnnnnnnnnnnnnnnnnnnnnnnnnnnnnnnnnnnnnnnnnnnnnnnnnnnnnnnnnnnnnnnnnnnnnnnnnnnnnnnnnnnnnnnnnnnnnnnnnnnnnnnnnnnnnnnnnnnnnnnnnnnnnnnnnnagcgcgtacgaacacgtaacagtgatcccgaacacggtgggagtaccgtataagactctagtcaatagaccgggctacagtcccatggtattggagatggaactactgtcagtcactttggagccaacgctatcgcttgattacatcacgtgcgagtacaaaaccgttatcccgtctccgtacgtgaaatgctgcggtacagcagagtgcaaggacaaaaacctacctgactacagctgtaaggtcttcaccggcgtctacccatttatgtggggcggagcctgccaaattcattgtggggccaatgtcttcagcctggacacctttcgacaacaaaattgtggtgtacaaaggtgacgtctataacatggactacccgccctttggcgcaggaagaccaggacaatttggcgatatccaaagtcgcacacctgagagtacagacgtctatgctaatacacaactggtactgcagagaccggctgcgggtacggtacatgtgccatactctcaggcaccatctggctttaagtattggctaaaagaacgaggggcgtcgctgcagcacacagcaccatttggctgccaaatagcaacaaacccggtaagagcggtgaattgcgccgtagggaacatgcccatctccatcgacataccgga

>MK156062

cgtctgcccgatgcgcagcgcagaagatcccgagagactcgccaattatgcgagaaagctagcatctgctgcaggaaaagtcctggacagaaacatctctggaaagatcggggacttacaagcagtaatggccgtgccagacacggagacgccaacattctgcttgcacacagatgtctcatgtagacagagagcagacgtcgcgatataccaagacgtctatgctgtacacgcacccacgtcgctataccaccaggcgattaaaggggtccgagtggcgtactgggtagggttcgacacaaccccgttcatgtacaatgccatggcgggtgcctacccctcatattcgacaaactgggcagacgagcaggtactgaaggctaagaacataggattatgttcaacagacctgacggaaggtagacgaggtaaattgtctattatgagagggaagaagctaaaaccgtgtgaccgtgtgctgttctcagtagggtcaacgctctacccggaaagccgcaagctacttaagagctggcacttaccatcggtgttccatctaaagggcaaactcagcttcacatgccgctgtgatacagtggtttcgtgcgagggctacgtcgttaagagaataacgatgagcccaggcctttatggaaaaaccacagggtatgcggtaacccaccacgcagacggattcttgatgtgcaagactaccgacacggttgacggcgaaagagtgtcattctcggtgtgcacatacgtgccggcgaccatttgtgatcaaatgaccggcatccttgctacagaagtcacgccggaggatgcacagaagctgttggtggggctgaaccagagaatagtggttaacggcagaacgcaacggaatacgaacaccatgaaaaactatctgcttcccgtggtcgcccaagccttcagtaagtgggcaaaggagtgccggaaagacatggaagatgaaaaactcctgggggtcagagaaagaacactgacctgctgctgtctatgggcattcaagaagcagaaaacacacacggtctacaagaggcctgatacccagtcaattcagaaggttcaggccgagtttgacagctttgtggtaccgagcctgtggtcgtccgggttgtcaatccccttgaggactagaatcaaatggttgttaagcaaggtgccaaaaaccgacctgatcccatatagcggggacgcccaagaagcccgggacgcagaaaaagaagcagaggaagaacgagaagcagaactgactcgtgaagccctaccacctctacaggcagcacaggaagatgttcaggtcgaaatcgacgtggaacagcttgaggacagagcgggtgcaggaataatagagactcctagaggagctatcaaagttactgcccaaccaacagaccacgtcgtgggagagtacctggtactttccccgcagaccgtactacggagccagaagctcagcctaattcacgctttggcggagcaagtgaagacgtgcacgcacagcggacgagcagggaggtacgcggtcgaagcgtacgacggccgagtcctagtgccctcaggctatgcaatctcgcctgaagacttccagagtctaagcgaaagcgcaacgatggtgtataacgaaagagagttcgtaaacagaaagctacaccacattgcgatgcacggaccagccctgaacaccgacgaagagtcgtatgagctggtgagggcagagaggacagaacacgagtacgtctacgatgtggaccagagaagatgctgtaagaaggaagaagctgcaggactggtattggtgggcgacttgactaatccgccctaccacgaattcgcatatgaagggctaaaaatccgccctgcctgcccatacaaaattgcagtcataggagtcttcggagtaccaggatctggcaagtcagctattatcaagaacctagttaccaggcaagacctggtgactagcggaaagaaagaaaactgccaagaaatcaccaccgacgtgatgagacagagaggtctagagatatctgcacgtacggttgactcgctgctcttgaatggatgcaacaggccagtcgacgtgttgtacgtagacgaggcgtttgcgtgccactctggaacgttacttgctttgatcgccttggttagaccaaggcagaaagttgtactctgtggtgacccgaagcagtgcggcttcttcaatatgatgcagatgaaagtcaactataatcacaacatctgcacccaagtgtaccacaaaagtatctccaggcggtgtacactgcctgtgaccgccattgtgtcatcgttacattacgaaggcaaaatgcgcactacgaatgagtacaacaagccgattgtagtggacactacaggctcaacaaaacctgacgctggagatctcgtgttaacgtgcttcagagggtgggttaaacaactgcaaattgactatcgtggatacgaggtcatgacagcagccgcatcccaagggttaaccagaaaaggagtttatgcagttaggcaaaaagttaatgaaaacccgctctatgcatcaacgtcagagcacgtcaacgtactcctaacgcgtacggaaggtaaactggtatggaagacactttccggtgacccgtggataaagacgctgcagaacccaccgaaaggaaacttcaaagcaactatcaaggagtgggaggtggagcatgcatcaataatggcgggcatctgcagtcaccaaatgacctttgatacattccaaaacaaagccaatgtttgttgggctaagagcttggtccctatcctcgaaacagcggggataaaactaaatgataggcagtggtcccagataattcaagccttcaaagaagataaagcatactcacctgaagtagccctgaatgaaatatgcacgcgcatgtatggggtggatctagacagtgggctattttctaaaccattggtgtctgtgtactactcggataaccactgggataataggcctggagggaagatgttcggattcaaccccgaggcagcatccattctagaaagaaagtatccattcacaaaagggaagtggaacatcaacaagcagatctgtgtgaccaccaggaggatagaagacttcaaccctaccaccaatattataccggccaataggagactaccacactcattagtggccgaacaccgcccagtaaaaggggaaagaatggaatggctggttaataagataaacggccaccacgtgctcctggtcagtggctataatcttgtactgcctactaagagagtcacttgggtagcgccactaggtgtccgcggagcggactacacatacaacctagagttgggtctgccagcaacgcttggcaggtatgacctagtggtcataaacatccacacaccttttcgcatacaccattaccaacagtgcgtagaccacgcaatgaaactgcaaatgctcgggggtgactcattgagactgctcaaaccgggtggctctctattgannnnnnnnnnnnnnnnnnnnnntagaaccagtgaacgagtcatctgcgtgttgggacgcaagttcagatcgtctagagcgttgaaaccaccatgtgtcaccagcaacactgagatgtttttcctattcagcaactttgacaatggcagaaggaatttcacaactcatgtcatgaataatcaactgaatgcagcctttgtaggacaggccacccgagcaggatgtgcaccgtcgtaccgggtaaaacgcatggacatcgcgaagaacgatgaagagtgcgtagttaacgccgctaaccctcgcgggttaccgggtgacggtgtttgcaaggcagtatacaaaaaatggccggagtcctttaagaatagtgcaacaccagtgggaaccgcaaaaacagtcatgtgcggtacgtatccagtaatccacgccgttggaccgaatttctctaattatttggaatctgaaggggaccgggaattggcagctgcctatcgagaagtcgcaaaggaagtaactagactgggagtaaatagtgtagctatacctctcctctccacaggtgtannnnnnnnnnnnnnnnnnnnnnnnnnnnnnnnnnnnnnnnnnnnnnnnnnnnnnnnnnnnnnnnnnnnnnnnnnnnnnnnnnnnnnnnnnnnnnnnnnnnnnnnnnnnnnnnnnnnntccnaggccatacagatgcggactcaagtggagttgctggatgagcacatctccatagactgcgatattgttcgcgtgcaccctgacagcagcttggcaggcngnaaaggatacagcaccacggaannnnnnnnnnnnnnnnnnnnnnnnnnnnnnnnnnnnnnnnnnnnggcagtggacatggcagagatatatactatgtggccaaagcaaacagaggccaacgagcaagtctgcctatatgccctgggggaaagtattgaatcgatcaggcagaaatgcccggtggatgatgcagacgcatcgtctcctccaaaaactgtcccgtgcctttgccgttacgccatgactccggaacgcgtcaccagacttcgcatgaaccacgtcacaagtataattgtgtgttcctcgtttccccttccaaagtacaaaatagaaggagtgcaaaaagtcaaatgctctaaggtaatgctatttgaccacaacgtgccatcacgcgtaagtccaagggaatatagatcttccccggaggctgcacaggaagcgagtacaaccacgtcactgacgcatagccaattcgacctaagcgttgatggcgagatactacccgtcccgtcagatctggatgctgacgccccagccctagaaccagctctagacgacagggcgacacacacgttgccatccacaaccggaaaccttgcggccgtgtctgactgggtaatgagcaccgtacctgtcgcgccgcccagaagaaggcgagggagaaacctgactgtgacatgcgacgagagagaagggaacataacacccatggctagcgtccgattctttagggcagagctgcgtccggtcgtacaagaaacagcggagacgcgtgacacagcaatgtctcttcaggcaccgccgagtaccgccacggaactgaatcatccgccgatctccttcggagcaccaagcgagacgttccccattacatttggggacttcaacgaaggagaaatcgaaagcttgtcttctgagctgctaactttcggagacttcctacccggagaagtggatgacttgacagatagcgactggtccacgtgctcagacacggacgacgagttactagacagggcaggtgggtatatattctcgtcggacaccggtccaggtcatttacaacagaagtcagtacgccagtcagtgctgccggtgaacaccctggaggaagtccacgaggagaagtgttacccacctaagttggatgaagtaaaggagcaactattacttaagaaactccaggagagtgcatccatggccaacagaagcaggtaccagtcgcgcaaagtagaaaacatgaaagcaacaatcatccagagactaaagagaggctgtagactatacttaatgtcagagacctcaaaagtccctacttaccggaccacatatccggcgcctgtgtactcgcctccggtcaacgtccgactgtccaaccccgagtccgcagtggcagcatgcaatgagttcctagctagaaactatccaactgtctcatcataccaaattaccgacgagtatgatgcatatttagacatggtggacgggtcggagagttgcctggatcgagcgacattcaatccgtcaaaacttaggagctacccgaaacagcacgcttaccatgcgccctccatcagaagcgctgtaccgtccccattccagaacacactacagaatgtactggcagcagccacgaaaagaaactgcaacgtcacacagatgagggaattacccactttggactcagcagtattcaacgtggagtgttttaaaaaattcgcatgcaaccaagaatactgggaagaatttgctgccagccctattaggataacaactgagaatttaacaacctatgttactaaactaaaagggccaaaagcagcagcgctatttgcaaaaanccacaatctgctgccactgcaggaagtaccaatggataggttcacagtagatatgaaaagggacgtgaaggtgactcctggtacaaagcacacagaggaaagacctaaggtgcaggttatacaggcggctgaacccttggcgacagcatacctatgtgggattcacagagagctggttaggaggctgaacgccgtcctcctacccaatgtacatacactatttgatatgtctgccgaggatttcgatgccatcatagccgcacactttaagccaggagacactgttttagaaacggacatagcctcctttgacaagagccaagatgattcacttgcgcttactgccttgatgttgttagaagatttaggggtggatcactccctgttggacttgatagaggccgcttttggagagatttccagctgtcatcnnnnnnnnnnnnnnnnnnnnnnnnnnggcgccatgatgaaatctggtatgttcctaactctgttcgtcaacacattgctaaacatcaccatcgctagccgcgtgctggaagatcgtctgacaaaatccgcgtgcgcggccttcatcggcgacgacaacataatacatggagtcgtctccgatgaattgatggcagacagatgcgccacttggatgaacatggaagtgaagatcatagatgcagttgtatccttgaaagccccttacttttgtggagggtttatactgcacgatactgtgacgggaacagcctgcagagtggcagacccgctaaaaaggctattcaaactgggcaaaccgctagcggcaggtgacgaacaagatgaagatagaagacgagcgctggctgacgaagtgatcagatggcaacgaacagggctaattgatgagttggagaaagcggtatactctaggtatgaagtgcagggtatatcagttgtggtaatgtccatggccacctttgcaagctccagatctaacttcgagaagctcagaggaccccagttaataaactgacaatgcgcgcggtaccccaacagaagccacgcaggaatcggaagaataagaagcaaaagcaaaagcgacaggcgccacaaaacaacacaaatcaaaagaagcagccgcctaaaaagaaaccagctcaaaagaaaaagaagccgggccgcagagagaggatgtgcatgaaaatcgaaaatgattgtatttttgaagtcaagtatgaaggtaaggtaacaggttacgcgtgcttggtgggggacaaagtaatgaaaccagcacacgtaaaggggaccatcgataacgcggacctggccaaactggcctttaagcggtcatctaagtatgaccttgaatgcgcgcagatacccgtgcacatgaagtccgacgcttcgaagttcacccatgagaaaccagaggggtactacaactggcaccacggagcagtacagtactcaggaggccggttcaccatccccacaggtgctggcaaaccaggggacagcggcagaccgatcttcgacaacaagggacgcgtggtggccatagtcttaggaggagctaatgaaggagcccgtacagccctctcggtggtgacctggaataaagacattgtcactaaaatcacccccgagggggccgaagagtggagtcttgccatcccagtcatgtgcctgctggcaaataccacgttcccctgctcccagcccccttgcactccctgctgctatgaaagggaaccagaggaaaccctacgcatgcttgaggacaacgtcatgagacctgggtactatcagctgctacaagcattaaagacggatgatagccacgattggaccaagctgcgttacatggacaaccacacgccagcggacgcagagagggcggggctatttgtaagaacatcagcaccgtgcacgattactggaacaatgggacacttcatcctgacccgatgtccgaaaggggaaactctgacggtgggattcactgacagtaggaagatcagtcactcatgtacgcacccatttcaccacgaccctcctgtgataggccgggagaaattccattcccgaccgcagcacngnaaagagctgccttgcagcacgtacgtgcagagcaccgccgcaactaccgaggagatagaggtacacatgcccccagacacccctgatcgcacactgatgtcacaacagtccggcaacgtaaagatcacagttaatggccagacggtgcggtacaagtgtaattgcggtggctcaaatgaaggactaataactacagacaaagtgattaataactgcaaagttgatcaatgtcatgccgcggtcaccaatcacaaaaagtggcagtacaactcccctctggtcccgcgtaatgctgaacttggggaccgaaaaggaaaaatccacatcccgtttccgctggcaaatgtaacatgcagggtgcctaaagcaaggaaccccaccgtgacgtacgggaaaaaccaagtcatcatgctactgtatcccgaccacccaacactcctgtcctaccggaacatgggagaagaaccaaactaccaagaagagtgggtgacgcataagaaggaagtcgtgctaaccgtgccgactgaagggctcgaggtcacgtggggtaacaacgagccgtataagtattggccgcagttatctacaaacggtacagcccatggccacccgcatgagataattctgtattattatgagctgtaccctactatgactgtagtagttgtgtcagtggcctcgttcgtactcctgtcgatggtgggtgtggcagtggggatgtgcatgtgtgcacgacgcagatgcannnnnnnnnacgaactgacaccaggagctaccgtccctttcctgcttagcctaatatgctgcatcagaacagctaaagcggccacataccaagaggctgcggtatacctgtggaatgaacagcaacctttgttttggctacaagcccttattccgctggcagccctgattgtcctgtgcaactgtctgagactcttaccatgctgctgtaaaacgttggcntttttagccgtaatgagcgtcggtgcccacactgtgagcgcgtacgaacacgtaacagtgatcccgaacacggtgggagtaccgtataagactctagtcaatagaccgggctacagtcccatggtattggagatggaactactgtcagtcactttggagccaacgctatcgcttgattacatcacgtgcgagtacaaaaccgttatcccgtctccgtacgtgaaatgctgcggtacagcagagtgcaaggacaaaaacctacctgactacagctgtaaggtcttcaccggcgtctacccatttatgtggggcggagcctgccaaattcattgtggggccaatgtcttcagcctggacacctttcgacaacaaaattgtggtgtacaaaggtgacgtctataacatggactacccgccctttggcgcaggaagaccaggacaatttggcgatatccaaagtcgcacacctgagagtacagacgtctatgctaatacacaactggtactgcagagaccggctgcgggtacggtacatgtgccatactctcaggcaccatctggctttaagtattggctaaaagaacgaggggcgtcgctgcagcacacagcaccatttggctgccaaatagcaacaaacccggtaagagcggtgaattgcgccgtagggaacatgcccatctccatcgacataccgga

>MK156058

cgtctgcccgatgcgcagcgcagaagatcccgagagactcgccaattatgcgagaaagctagcatctgctgcaggaaaagtcctggacagaaacatctctggaaagatcggggacttacaagcagtaatggccgtgccagacacggagacgccaacattctgcttgcacacagatgtctcatgtagacagagagcagacgtcgcgatataccaagacgtctatgctgtacacgcacccacgtcgctataccaccaggcgattaaaggggtccgagtggcgtactgggtagggttcgacacaaccccgttcatgtacaatgccatggcgggtgcctacccctcatattcgacaaactgggcagacgagcaggtactgaaggctaagaacataggattatgttcaacagacctgacggaaggtagacgaggtaaattgtctattatgagagggaagaagctaaaaccgtgtgaccgtgtgctgttctcagtagggtcaacgctctacccggaaagccgcaagctacttaagagctggcacttaccatcggtgttccatctaaagggcaaactcagcttcacatgccgctgtgatacagtggtttcgtgcgagggctacgtcgttaagagaataacgatgagcccaggcctttatggaaaaaccacagggtatgcggtaacccaccacgcagacggattcttgatgtgcaagactaccgacacggttgacggcgaaagagtgtcattctcggtgtgcacatacgtgccggcgaccatttgtgatcaaatgaccggcatccttgctacagaagtcacgccggaggatgcacagaagctgttggtggggctgaaccagagaatagtggttaacggcagaacgcaacggaatacgaacaccatgaaaaactatctgcttcccgtggtcgcccaagccttcagtaagtgggcaaaggagtgccggaaagacatggaagatgaaaaactcctgggggtcagagaaagaacactgacctgctgctgtctatgggcattcaagaagcagaaaacacacacggtctacaagaggcctgatacccagtcaattcagaaggttcaggccgagtttgacagctttgtggtaccgagcctgtggtcgtccgggttgtcaatccccttgaggactagaatcaaatggttgttaagcaaggtgccaaaaaccgacctgatcccatatagcggggacgcccaagaagcccgggacgcagaaaaagaagcagaggaagaacgagaagcagaactgactcgtgaagccctaccacctctacaggcagcacaggaagatgttcaggtcgaaatcgacgtggaacagcttgaggacagagcgggtgcaggaataatagagactcctagaggagctatcaaagttactgcccaaccaacagaccacgtcgtgggagagtacctggtactttccccgcagaccgtactacggagccagaagctcagcctaattcacgctttggcggagcaagtgaagacgtgcacgcacagcggacgagcagggaggtacgcggtcgaagcgtacgacggccgagtcctagtgccctcaggctatgcaatctcgcctgaagacttccagagtctaagcgaaagcgcaacgatggtgtataacgaaagagagttcgtaaacagaaagctacaccacattgcgatgcacggaccagccctgaacaccgacgaagagtcgtatgagctggtgagggcagagaggacagaacacgagtacgtctacgatgtggaccagagaagatgctgtaagaaggaagaagctgcaggactggtattggtgggcgacttgactaatccgccctaccacgaattcgcatatgaagggctaaaaatccgccctgcctgcccatacaaaattgcagtcataggagtcttcggagtaccaggatctggcaagtcagctattatcaagaacctagttaccaggcaagacctggtgactagcggaaagaaagaaaactgccaagaaatcaccaccgacgtgatgagacagagaggtctagagatatctgcacgtacggttgactcgctgctcttgaatggatgcaacaggccagtcgacgtgttgtacgtagacgaggcgtttgcgtgccactctggaacgttacttgctttgatcgccttggttagaccaaggcagaaagttgtactctgtggtgacccgaagcagtgcggcttcttcaatatgatgcagatgaaagtcaactataatcacaacatctgcacccaagtgtaccacaaaagtatctccaggcggtgtacactgcctgtgaccgccattgtgtcatcgttacattacgaaggcaaaatgcgcactacgaatgagtacaacaagccgattgtagtggacactacaggctcaacaaaacctgacgctggagatctcgtgttaacgtgcttcagagggtgggttaaacaactgcaaattgactatcgtggatacgaggtcatgacagcagccgcatcccaagggttaaccagaaaaggagtttatgcagttaggcaaaaagttaatgaaaacccgctctatgcatcaacgtcagagcacgtcaacgtactcctaacncgtacggaaggtaaactggtatggaagacactttccggtgacccgtggataaagacgctgcagaacccaccgaaaggaaacttcaaagcaactatcaaggagtgggaggtggagcatgcatcaataatggcgggcatctgcagtcaccaaatgacctttgatacattccaaaacaaagccaatgtttgttgggctaagagcttggtccctatcctcgaaacagcggggataaaactaaatgataggcagtggtcccagataattcaagccttcaaagaagataaagcatactcacctgaagtagccctgaatgaaatatgcacgcgcatgtatggggtggatttagacagtgggctattttctaaaccattggtgtctgtgtactactcggataaccactgggataataggcctggagggaagatgttcggattcaaccccgaggcagcatccattctagaaagaaagtatccattcacaaaagggaagtggaacatcaacaagcagatctgtgtgaccaccaggaggatagaagactttaaccctaccaccaatattataccggccaataggagactaccacactcattagtggccgaacaccgcccagtaaaaggggaaagaatggaatggctggttaataagataaacggccaccacgtgctcctggtcagtggctataatcttgtactgcctactaagagagtcacttgggtagcgccactaggtgtccgcggagcggactacacatacaacctagagttgggtctgccagcaacgcttggcaggtatgacctagtggtcataaacatccacacaccttttcgcatacaccattaccaacagtgcgtagaccacgcaatgaaactgcaaatgctcgggggtgactcattgagactgctcaaaccgggtggctctctattgatcagagcatatggttacgcagatagaaccagtgaacgagtcatctgcgtgttgggacgcaagttcagatcgtctagagcgttgaaaccaccatgtgtcaccagcaacactgagatgtttttcctattcagcaactttgacaatggcagaaggaatttcacaactcatgtcatgaataatcaactgaatgcagcctttgtaggacaggccacccgagcaggatgtgcaccgtcgtaccgggtaaaacgcatggacatcgcgaagaacgatnnagngtgcnnnnnnnnnnnnnnnnnnnnnnnnnnnnnnnnnnnnnnnnnnnnnnnnnnnnnnnnnnnnnnnnnnnnnnnnnnnnnnnnnnnnnnnnnnnnnnnnnnnnnnnnnnnnnnnnnnnnnnnnnnnnnnnnnnnnnnnnnnnnnnnnnnnnnnnnnnnnnnnnnnnnnnnnnnnnnnnnnnnnnnnnnnnnnnnnnnnnnnnnnnnnnnnnnnnnnnnnnnnnnnnnnnnnnnnnnnnnnnnnnnnnnnnnnnnnnnnnnnnnnnnnnnnnnnnnnnnnnnnnnnnnnnnnnnnnnnnnnnnnnnnnnnnnnnnnnnnnnnnnnnnncancncttcacagccatggactcgacggatgcagacgtggtcatctactgtcgcgacaaagaatgggagaagaaaatatccgaggccatacagatgcggactcaagtggagttgctggatgagcacatctccatagactgcgatattgttcgcgtgcaccctgacagcagcttggcaggcagaaaaggatacagcaccacggaaggcgcactgtactcatatctagaagggacncgntttcaccagncggcagtggacatggcagagatatatactatgtggccaaagcaaacagaggccaacgagcaagtctgcctatatgccctgggggaaagtattgaatcgatcaggcagaaatgcccggtggatgatgcagacgcatcgtctcctccaaaaactgtcccgtgcctttgccgttacgccatgactccggaacgcgtcaccagacttcgcatgaaccacgtcacaagtataattgtgtgttcctcgtttccccttccaaagtacaaaatagaaggagtgcaaaaagtcaaatgctctaaggtaatgctatttgaccacaacgtgccatcacgcgtaagtccaagggaatatagatcttccccggaggctgcacaggaagcgagtacaaccacgtcactgacgcatagccaattcgacctaagcgttgatggcgagatactacccgtcccgtcagatctggatgctgacgccccagccctagaaccagctctagacgacagggcgacacacacgttgccatccacaaccggaaaccttgcggccgtgtctgactgggtaatgagcaccgtacctgtcgcgccgcccagaagaaggcgagggagaaacctgactgtgacatgcgacgagagagaagggaacataacacccatggctagcgtccgattctttagggcagagctgcgtccggtcgtacaagaaacagcggagacgcgtgacacagcaatgtctcttcaggcaccgccgagtaccgccacggaactgaatcatccgccgatctccttcggagcaccaagcgagacgttccccattacatttggggacttcaacgaaggagaaatcgaaagcttgtcttctgagctgctaactttcggagacttcctacccggagaagtggatgacttgacagatagcgactggtccacgtgctcagacacggacgacgagttactagacagggcaggtgggtatatattctcgtcggacaccggtccaggtcatttacaacagaagtcagtacgccagtcagtgctgccggtgaacaccctggaggaagtccacgaggagaagtgttacccacctaagttggatgaagtaaaggagcaactattacttaagaaactccaggagagtgcatccatggccaacagaagcaggtaccagtcgcgcaaagtagaaaacatgaaagcaacaatcatccagagactaaagagaggctgtagactatacttaatgtcagagacctcaaaagtccctacttaccggaccacatatccggcgcctgtgtactcgcctccggtcaacgtccgactgtccaaccccgagtccgcagtggcagcatgcaatgagttcctagctagaaactatccaactgtctcatcataccaaattaccgacgagtatgatgcatatttagacatggtggacgggtcggagagttgcctggatcgagcgacattcaatccgtcaaaacttaggagctacccgaaacagcacgcttaccatgcgccctccatcagaagcgctgtaccgtccccattccagaacacactacagaatgtactggcagcagccacgaaaagaaactgcaacgtcacacagatgagggaattacccactttggactcagcagtattcaacgtggagtgttttaaaaaattcgcatgcaaccaagaatactgggaagaatttgctgccagccctattaggataacaactgagaatttaacaacctatgttactaaactaaaagggccaaaagcagcagcgctatttgcaaaaanccacaatctgctgccactgcaggaagtaccaatggataggttcacagtagatatgaaaagggacgtgaaggtgactcctggtacaaagcacacagaggaaagacctaaggtgcaggttatacaggcggctgaacccttggcgacagcatacctatgtgggattcacagagagctggttaggaggctgaacgccgtcctcctacccaatgtacatacactatttgatatgtctgccgaggatttcgatgccatcatagccgcacactttaagccaggagacactgttttagaaacggacatagcctcctttgacaagagccaagatgattcacttgcgcttactgccttgatgttgttagaagatttaggggtggatcactccctgttggacttgatagaggccgcttttggagagatttccagctgtcatctaccgnnnnnnnnnnnnnnnnnnnnnggcgccatgatgaaatctggtatgttcctaactctgttcgtcaacacattgctaaacatcaccatcgctagccgcgtgctggaagatcgtctgacaaaatccgcgtgcgcggccttcatcggcgacgacaacataatacatggagtcgtctccgatgaattgatggcagacagatgcgccacttggatgaacatggaagtgaagatcatagatgcagttgtatccttgaaagccccttacttttgtggagggtttatactgcacgatactgtgacgggaacagcctgcagagtggcagacccgctaaaaaggctattcaaactgggcaaaccgctagcggcaggtgacgaacaagatgaagatagaagacgagcgctggctgacgaagtgatcagatggcaacgaacagggctaattgatgagttggagaaagcggtatactctaggtatgaagtgcagggtatatcagttgtggtaatgtccatggccacctttgcaagctccagatctaacttcgagaagctcagaggaccccagttaataaactgacaatgcgcgcggtaccccaacagaagccacgcaggaatcggaagaataagaagcaaaagcaaaagcgacaggcgccacaaaacaacacgaatcaaaagaagcagccgcctaaaaagaaaccagctcaaaagaaaaagaagccgggccgcagagagaggatgtgcatgaaaatcgaaaatgattgtatttttgaagtcaagtatgaaggtaaggtaacaggttacgcgtgcttggtgggggacaaagtaatgaaaccagcacacgtaaaggggaccatcgataacgcggacctggccaaactggcctttaagcggtcatctaagtatgaccttgaatgcgcgcagatacccgtgcacatgaagtccgacgcttcgaagttcacccatgagaaaccagaggggtactacaactggcaccacggagcagtacagtactcaggaggccggttcaccatccccacaggtgctggcaaaccaggggacagcggcagaccgatcttcgacaacaagggacgcgtggtggccatagtcttaggaggagctaatgaaggagcccgtacagccctctcggtggtgacctggaataaagacattgtcactaaaatcacccccgagggggccgaagagtggagtcttgccatcccagtcatgtgcctgctggcaaataccacgttcccctgctcccagcccccttgcactccctgctgctatgaaagggaaccagaggaaaccctacgcatgcttgaggacaacgtcatgagacctgggtactatcagctgctacaagcattaaagacggatgatagccacgattggaccaagctgcgttacatggacaaccacacgccagcggacgcagagagggcggggctatttgtaagaacatcagcaccgtgcacgattactggaacaatgggacacttcatcctgacccgatgtccgaaaggggaaactctgacggtgggattcactgacagtaggaagatcagtcactcatgtacgcacccatttcaccacgaccctcctgtgataggccgggagaaattccattcccgaccgcagcacggtaaaganctnccttgcagcacgtacgtgcagagcaccgccgcaactaccgaggagatagaggtacacatgcccccagacacccctgatcgcacactgatgtcacaacagtccggcaacgtaaagatcacagttaatggccagacggtgcggtacaagtgtaattgcggtggctcaaatgaaggactaataactacagacaaagtgattaataactgcaaagttgatcaatgtcatgccgcggtcaccaatcacaaaaagtggcagtacaactcccctctggtcccgcgtaatgctgaacttggggaccgaaaaggaaaaatccacatcccgtttccgctggcaaatgtaacatgcagggtgcctaaagcaaggaaccccaccgtgacgtacgggaaaaaccaagtcatcatgctactgtatcccgaccacccaacactcctgtcctaccggaacatgggagaagaaccaaactaccaagaagagtgggtgacgcataagaaggaagtcgtgctaaccgtgccgactgaagggctcgaggtcacgtggggtaacaacgagccgtataagtattggccgcagttatctacaaacggtacagcccatggccacccgcatgagataattctgtattattatgagctgtaccctactatgactgtagtagttgtgtcagtggcctcgttcgtactcctgtcgatggtgggtgtggcagtggggatgtgcatgtgtgcacgacgcagatgcannncaccntacgaactgacaccaggagctaccgtccctttcctgcttagcctaatatgctgcatcagaacagctaaagcggccacataccaagaggctgcggtatacctgtggaatgaacagcaacctttgttttggctacaagcccttattccgctggcagccctgattgtcctntgcaactgtctgagactcttaccatgctgctnnnaaacgtnnnnnnnnnnnnnnnnnnnnnnnnnnngtgcccacactgtgagcgcgtacgaacacgtaacagtgatcccgaacacggtgggagtaccgtataagactctagtcaatagaccgggctatagtcccatggtattggagatggaactactgtcagtcactttggagccaacgctatcgcttgattacatcacgtgcgagtacaaaaccgttatcccgtctccgtacgtgaaatgctgcggtacagcagagtgcaaggacaaaaacctacctgactacagctgtaaggtcttcaccggcgtctacccatttatgtggggcggagcctgccaaattcattgtggggccaatgtcttcagcctggacacctttcgacaacaaaattgtggtgtacaaaggtgacgtctataacatggactacccgccctttggcgcaggaagaccaggacaatttggcgatatccaaagtcgcacacctgagagtacagacgtctatgctaatacacaactggtactgcagagaccggctgcgggtacggtacatgtgccatactctcaggcaccatctggctttaagtattggctaaaagaacgaggggcgtcgctgcagcacacagcaccatttggctgccaaatagcaacaaacccggtaagagcggtgaattgcgccgtagggaacatgcccatctccatcgacataccgga

>MK156059

cgtctgcccgatgcgcagcgcagaagatcccgagagactcgccaattatgcgagaaagctagcatctgctgcaggaaaagtcctggacagaaacatctctggaaagatcggggacttacaagcagtaatggccgtgccagacacggagacgccaacattctgcttgcacacagatgtctcatgtagacagagagcagacgtcgcgatataccaagacgtctatgctgtacacgcacccacgtcgctataccaccaggcgattaaaggggtccgagtggcgtactgggtagggttcgacacaaccccgttcatgtacaatgccatggcgggtgcctacccctcatattcgacaaactgggcagacgagcaggtactgaaggctaagaacataggattatgttcaacagacctgacggaaggtagacgaggtaaattgtctattatgagagggaagaagctaaaaccgtgtgaccgtgtgctgttctcagtagggtcaacgctctacccggaaagccgcaagctacttaagagctggcacttaccatcggtgttccatctaaagggcaaactcagcttcacatgccgctgtgatacagtggtttcgtgcgagggctacgtcgttaagagaataacgatgagcccaggcctttatggaaaaaccacagggtatgcggtaacccaccacgcagacggattcttgatgtgcaagactaccgacacggttgacggcgaaagagtgtcattctcggtgtgcacatacgtgccggcgaccatttgtgatcaaatgaccggcatccttgctacagaagtcacgccggaggatgcacagaagctgttggtggggctgaaccagagaatagtggttaacggcagaacgcaacggaatacgaacaccatgaaaaactatctgcttcccgtggtcgcccaagccttcagtaagtgggcaaaggagtgccggaaagacatggaagatgaaaaactcctgggggtcagagaaagaacactgacctgctgctgtctatgggcattcaagaagcagaaaacacacacggtctacaagaggcctgatacccagtcaattcagaaggttcaggccgagtttgacagctttgtggtaccgagcctgtggtcgtccgggttgtcaatccccttgaggactagaatcaaatggttgttaagcaaggtgccaaaaaccgacctgatcccatatagcggggacgcccaagaagcccgggacgcagaaaaagaagcagaggaagaacgagaagcagaactgactcgtgaagccctaccacctctacaggcagcacaggaagatgttcaggtcgaaatcgacgtggaacagcttgaggacagagcgggtgcaggaataatagagactcctagaggagctatcaaagttactgcccaaccaacagaccacgtcgtgggagagtacctggtactttccccgcagaccgtactacggagccagaagctcagcctaattcacgctttggcggagcaagtgaagacgtgcacgcacagcggacgagcagggaggtacgcggtcgaagcgtacgacggccgagtcctagtgccctcaggctatgcaatctcgcctgaagacttccagagtctaagcgaaagcgcaacgatggtgtataacgaaagagagttcgtaaacagaaagctacaccacattgcgatgcacggaccagccctgaacaccgacgaagagtcgtatgagctggtgagggcagagaggacagaacacgagtacgtctacgatgtggaccagagaagatgctgtaagaaggaagaagctgcaggactggtattggtgggcgacttgactaatccgccctaccacgaattcgcatatgaagggctaaaaatccgccctgcctgcccatacaaaattgcagtcataggagtcttcggagtaccaggatctggcaagtcagctattatcaagaacctagttaccaggcaagacctggtgactagcggaaagaaagaaaactgccaagaaatcaccaccgacgtgatgagacagagaggtctagagatatctgcacgtacggttgactcgctgctcttgaatggatgcaacaggccagtcgacgtgttgtacgtagacgaggcgtttgcgtgcnnnnnnnnnnnnnnnnnnnnnnnnnnnnnnnnnnnnnnnnnnnnnnnnnnnnnnnnnnnnnnnnnnnnnnnnnnnnnnnnnnnnnnnnnnnnnnnnnnnnnnnnnnnnnnnnnnnnnnnnnnnnnnnnnnnnnnnnnnnnnnnnnnnnnnnnnnnnnnnnnnnnnnnnnnnnnnnnnnnnnnnnnnnnnnnnnnnnnnnnnnnnnnnnnnnnnnnnnnnnnnnnnnnnnnnnagtacaacaagccgattgtagtggacactacaggctcaacaaaacctgacgctggagatctcgtgttaacgtgcttcagagggtgggttaaacaactgcaaattgactatcgtggatacgaggtcatgacagcagccgcatcccaagggttaaccagaaaaggagtttatgcagttaggcaaaaagttaatgaaaacccgctctatgcatcaacgtcagagcacgtcaacgnnntcctaacacgtacggaaggtaaactggtatggaagacactttccggtgacccgtggataaagacgctgcagaacccaccgaaaggaaacttcaaagcaactatcaaggagtgggaggtggagcatgcatcaataatggcgggcatctgcagtcaccaaatgacctttgatacattccaaaacaaagccaatgtttgttgggctaagagcttggtccctatcctcgaaacagcggggataaaactaaatgataggcagtggtcccagataattcaagccttcaaagaagataaagcatactcacctgaagtagccctgaatgaaatatgcacgcgcatgtatggggtggatttagacagtgggctattttctaaaccattggtgtctgtgtactactcggataaccactgggataataggcctggagggaagatgttcggattcaaccccgaggcagcatccattctagaaagaaagtatccattcacaaaagggaagtggaacatcaacaagcagatctgtgtgaccaccaggaggatagaagactttaaccctaccaccaatattataccggccaataggagactaccacactcattagtggccgaacaccgcccagtaaaaggggaaagaatggaatggctggttaataagataaacggccaccacgtgctcctggtcagtggctataatcttgtactgcctactaagagagtcacttgggtagcgccactaggtgtccgcggagcggactacacatacaacctagagttgggtctgccagcaacgcttggcaggtatgacctagtggtcataaacatccacacaccttttcgcatacaccattaccaacagtgcgtagaccacgcaatgaaactgcaaatgctcgggggtgactcattgagactgctcaaaccgggtggctctctattgatcagagcatatggttacgcagatagaaccagtgaacgagtcatctgcgtgttgggacgcaagttcagatcgtctagagcgttgaaaccaccatgtgtcaccagcaacactgagatgtttttcctattcagcaactttgacaatggcagaaggaatttcacaactcatgtcatgaataatcaactgaatgcagcctttgtaggacaggccacccgagcaggatgtgcaccgtcgtaccgggtaaaacgcatggacatcgcgaagaacgatgnagngtgcnnnnnnnnnnnnnnnnnnnnnnnnnnnnnnnnnnnnnnnnnnnnnnnnnnnnnnnnnnnnnnnnnnnnnnnnnnnnnnnnnnnnnnnnnnnnnnnnnnnnnnnnnnnnnnnnnnnnnnnnnnnnnnnnnnnnnnnnnnnnnnnnnnnnnnnnnnnnnnnnnnnnnnnnnnnnnnnnnnnnnnnnnnnnnnnnnnnnnnnnnnnnnnnnnnnnnnnnnnnnnnnnnnnnnnnnnnnnnnnnnnnnnnnnnnnnnnnnnnnnnnnnnnnnnnnnnnnnnnnnnnnnnnnnnnnnnnnnnnnnnnnnnnnnnnnnnnnnnnnnnnnnnnnnnnnnnnnnnnnnnnnnnnnnnnnnnnnnnnnnnnnnnnnnnnnnnnnnnnnnnnnnnnnnnnnnnnnnnnnnnnnnnnnnnnnnnnnnnnnnnnnnnnnnnnnnnnnnnnnnnnnnnnnnnnnnnnnnnnnnnnnnnnnnnnnnnnnnnnnnnnnnnnnnnnnnnnnnnnnnnnnnnnnnnnnnnnnnnnnnnnnnnnnnnnnnnnnnnnnnnnnnnnnnnnnnnnnnnnnnnnnnnnnnnnnnggcagtggacatggcagagatatatactatgtggccaaagcaaacagaggccaacgagcaagtctgcctatatgccctnggggaaagtattgaatcgatcaggcagaaatgcccggtggatgatgcagangcatcgtctcctccnaaaactgtcccgtgcctttgccgttacgccatgactccggaacgcgtcaccngacttcgcatgaaccnnnnnncaagtataattgtgtgttcctcgtttccccttccaaagtacaaaatagaaggagtgcaaaaagtcaaatgctctaaggtaatgctatttgaccacaacgtgccatcacgcgtaagtccaagggaatatagatcttccccggaggctgcacaggaagcgagtacaaccacgtcactgacgcatagccaattcgacctaagcgttgatggcgagatactacccgtcccgtcagatctggatgctgacgccccagccctagaaccagctcnnnnnnnnnnnnnnnnnnnnnnnnnnnnnnnnnnnnnnnnnnnnnnnnnnnnnnnnnnnnnnnnnnnnnnnnnnnnnnnnnnnnnnnnnnnnnnnnnnnnnnnnnnnnnnnnnnnnnnnnnnnnnnnnnnnnnnnnnnnnnnnnnnnnnnnnnnnnnnnnnnnnnnnnnnnnnnnnnnnnnnnnnnnnnnnnnnnnnnnnnnnnnnnnnnnnnnnnnnnnnnnnnnnnnnnnnnnnnnnnnncaggcaccgccgagtaccgccacggaactgaatcatccgccgatctccttcggagcaccaagnnnnnnnnnnnnnnnnnnnnnnnnnnnnnnnnnnnnnnnnnnnnnnnnnnnnnnnnnnnnnnnnnnnnnnnnnnnnnnnnnnnnnnnnnnnnnnnnnnnnnnnnnnnnnnnnnnnnnnnnnnnnnnnnnnnnnnnnnnnnnnnnnnnnnnnnnnnnagacagggcaggtgggtatatattctcgtcggacaccggtccaggtcatttacaacagaagtcagtacgccagtcagtgctgccggtgaacaccctggaggaagtccacgtggagaagtgttacccacctaagttggatgaagtaaaggagcaactattacttaagaaactccaggagagtgcatccatggccaacagaagcaggtaccagtcgcgcaaagtagaaaacatgaaagcaacaatcatccagagactaaagagaggctgtagactatacttaatgtcagagacctcaaaagtccctacttaccggaccacatatccggcgcctgtgtactcgcctccggtcaacgtccgactgtccaaccccgagtccgcagtggcagcatgcaatgagttcctagctagaaactatccaactgtctcatcataccaaattaccgacgagtatgatgcatatttagacatggtggacgggtcggagagttgcctggatcgagcgacattcaatccgtcaaaacttaggagctacccgaaacagcacgcttaccatgcgccctccatcagaagcgctgtaccgtccccattccagaacacactacagaatgtactggcagcagccacgaaaagaaactgcaacgtcacacagatgagggaattacccactttggactcagcagtattcaacgtggagtgttttaaaaaattcgcatgcaaccaagaatactgggaagaatttgctgccagccctattaggataacaactgagaatttaacaacctatgttactaaactaaaagggccaaaagcagcagcgctatttgcnnnnnnccacaatctgctgccactgcaggaagtaccaatggataggttcacagtagatatgaaaagggacgtgaaggtgactcctggtacaaagcacacagaggaaagacctaaggtgcaggttatacaggcggctgaacccttggcgacagcatacctatgtgggattcacagagagctggttaggaggctgaacgccgtcctcctacccaatgtacatacactatttgatatgtctgccgaggatttcgatgccatcatagccgcacactttaagccaggagacactgttttagaaacggacatagcctcctttgacaagagccaagatgattcacttgcgcttactgccttgatgttgttagaagatttaggggtggatcactccctgttggacttgatagaggccgcttttggagagatttccagctgtcannnnnnnnnnnnnnnnnnnnnnnnnnnnggcgccatgatgaaatctggtatgttcctaactctgttcgtcaacacattgctaaacatcaccatcgctagccgcgtgctggaagatcgtctgacaaaatccgcgtgcgcggccttcatcggcgacgacaacataatacatggagtcgtctccgatgaattgatggcagacagatgcgccacttggatgaacatggaagtgaagatcatagatgcagttgtatccttgaaagccccttacttttgtggagggtttatactgcacgatactgtgacgggaacagcctgcagagtggcagacccgctaaaaaggctattcaaactgggcaaaccgctagcggcaggtgacgaacaagatgaagatagaagacgagcgctggctgacgaagtgatcagatggcaacgaacagggctaattgatgagttggagaaagcggtatactctaggtatgaagtgcagggtatatcagttgtggtaatgtccatggccacctttgcaagctccagatctaacttcgagaagctcagaggaccccagttaataaactgacaatgcgcgcggtaccccaacagaagccacgcaggaatcggaagaataagaagcaaaagcaaaagcgacaggcgccacaaaacaacacaaatcaaaagaagcagccgcctaaaaagaaaccagctcaaaagaaaaagaagccgggccgcagagagaggatgtgcatgaaaatcgaaaatgattgtatttttgaagtcaagtatgaaggtaaggtaacaggttacgcgtgcttggtgggggacaaagtaatgaaaccagcnnnnnnnnnnnnnnnnnnnnnnnnnnnnnnnnnnnnnnnnnnnnnnnnnnnnnnnnnnnnnnnnnnnnnnnnnnnnnnnnnnnnnnnnnnnnnnnnnnnnnnnnnnnnnnnnnnnnnnnnnnnnnnnnnnnnnnnnnnnnnnnnnnnnnnnnnnnnnnnnnnnnnnnnnnnnnnnnnnnnnnnnnnnnnnnnnnnnnnnnnnnnnnnnnnnnnnnnnnnnnnnnnnnnnnnnnnnnnnnnnnnnnnnnnnnnacgcgtggtggccatagtcttaggaggagctaatgaaggagcccgtacagccctctcggtggtgacctggaataaagacattgtcactaaaatcacccccgagggggccgaagagtggagtcttgccatcccagtcatgtgcctgctggcaaataccacgttcccctgctcccagcccccttgcactccctgctgctatgaaagggaaccagaggaaaccctacgcatgcttgaggacaacgtcatgagacctgggtactatcagctgctacaagcatnnnnnncggatgatagccacgattggaccaagctgcgttacatggacaaccacacgccagcggacgcagagagggcggggctatttgtaagaacatcagcaccgtgcacgattactggaacaatgggacacttcatcctgacccgatgtccgaaaggggaaactctgacggtgggattcactgacagtaggaagatcagtcactcatgtacgcacccatttcaccacgaccctcctgtgataggccgggagaaattccattcccgaccgcagcacggtaaaganctnccttgcagcacgtacgtgcagagcaccgccgcaactaccgaggagatagaggtacacatgcccccagacacccctgatcgcacactgatgtcacaacagtccggcaacgtaaagatcacagttaatggccagacggtgcggtacaagtgtaattgcggtggctcaaatgaaggactaataactacagacaaagtgattaataactgcaaagttgatcaatgtcatgccgcggtcaccaatcacaaaaagtggcagtacaactcccctctggtcccgcgtaatgctgaacttggggaccgaaaaggaaaaatccacatcccgtttccgctggcaaatgtaacatgcagggtgcctaaagcaaggaaccccaccgtgacgtacgggaaaaaccaagtcatcatgctactgtatcccgaccacccaacactcctgtcctaccggaacatgggagaagaaccaaactaccaagaagagtgggtgacgcataagaaggaagtcgtgctaaccgtgccgactgaagggctcgnggtcacgtngggtaacaacgagccgtataagtattggccgcagttatctacaaacggtacagcccatggccacccgcatgagataattctgtattattatgagctgtaccctactatgactgtagtagttgtgtcagtggccnngtnnnnnnnnnnnnnnnnnnnnnnnnnnnnnnnnnnnnnnnnnnnnnnnnnnnnnnnnnnnnnnnnnnnnnnnnnngaactgacaccaggagctaccgtccctttcctgcttagcctaatatgctgcatcagaacagctaaagcggccacataccaagaggctgcggtatacctgtggaatgaacagcaacctttgttttggctacaagcccttattccgctggcagccctgattgtcctntgcaactgtcnnnnnnnnnnnnnnnnnnnnnnnnnnnnnnnnnnnnnnnnnnnnnnnnnnnnnnnnngtgcccacactgtgagcgcgtacgaacacgtaacagtgatcccgaacacggtgggagtaccgtataagactctagtcaatagaccgggctacagtcccatggtattggagatggaactactgtcagtcactttggagccaacgctatcgcttgattacatcacgtgcgagtacaaaaccgttatcccgtctccgtacgtgaaatgctgcggtacagcagagtgcaaggacaaaaacctacctgactacagctgtaaggtcttcaccggcgtctacccatttatgtggggcggagcctgccaaattcattgtggggccaatgtcttcagcctggacacctttcgacaacaaaattgtggtgtacaaaggtgacgtctataacatggactacccgccctttggcgcaggaagaccaggacaatttggcgatatccaaagtcgcacacctgagagtacagacgtctatgctaatacacaactggtactgcagagaccggctgcgggtacggtacatgtgccatactctcaggcaccatctggctttaagtattggctaaaagaacgaggggcgtcgctgcagcacacagcaccatttggctgccaaatagcaacaaacccggtaagagcggtgaattgcgccgtagggaacatgcccatctccatcgacataccgga

>MK156063

cgtctgcccgatgcgcagcgcagaagatcccgagagactcgccaattatgcgagaaagctagcatctgctgcaggaaaagtcctggacagaaacatctctggaaagatcggggacttacaagcagtaatggccgtgccagacacggagacgccaacattctgcttgcacacagatgtctcatgtagacagagagcagacgtcgcgatataccaagacgtctatgctgtacacgcacccacgtcgctataccaccaggcgattaaaggggtccgagtggcgtactgggtagggttcgacacaaccccgttcatgtacaatgccatggcgggtgcctacccctcatattcgacaaactgggcagacgagcaggtactgaaggctaagaacataggattatgttcaacagacctgacggaaggtagacgaggtaaattgtctattatgagagggaagaagctaaaaccgtgtgaccgtgtgctgttctcagtagggtcaacgctctacccggaaagccgcaagctacttaagagctggcacttaccatcggtgttccatctaaagggcaaactcagcttcacatgccgctgtgatacagtggtttcgtgcgagggctacgtcgttaagagaataacgatgagcccaggcctttatggaaaaaccacagggtatgcggtaacccaccacgcagacggattcttgatgtgcaagactaccgacacggttgacggcgaaagagtgtcattctcggtgtgcacatacgtgccggcgaccatttgtgatcaaatgaccggcatccttgctacagaagtcacgccggaggatgcacagaagctgttggtggggctgaaccagagaatagtggttaacggcagaacgcaacggaatacgaacaccatgaaaaactatctgcttcccgtggtcgcccaagccttcagtaagtgggcaaaggagtgccggaaagacatggaagatgaaaaactcctgggggtcagagaaagaacactgacctgctgctgtctatgggcattcaagaagcagaaaacacacacggtctacaagaggcctgatacccagtcaattcagaaggttcaggccgagtttgacagctttgtggtaccgagcctgtggtcgtccgggttgtcaatccccttgaggactagaatcaaatggttgttaagcaaggtgccaaaaaccgacctgatcccatatagcggggacgcccaagaagcccgggacgcagaaaaagaagcagaggaagaacgagaagcagaactgactcgtgaagccctaccacctctacaggcagcacaggaagatgttcaggtcgaaatcgacgtggaacagcttgaggacagagcgggtgcaggaataatagagactcctagaggagctatcaaagttactgcccaaccaacagaccacgtcgtgggagagtacctggtactttccccgcagaccgtactacggagccagaagctcagcctaattcacgctttggcggagcaagtgaagacgtgcacgcacagcggacgagcagggaggtacgcggtcgaagcgtacgacggccgagtcctagtgccctcaggctatgcaatctcgcctgaagacttccagagtctaagcgaaagcgcaacgatggtgtataacgaaagagagttcgtaaacagaaagctacaccacattgcgatgcacggaccagccctgaacaccgacgaagagtcgtatgagctggtgagggcagagaggacagaacacgagtacgtctacgatgtggaccagagaagatgctgtaagaaggaagaagctgcaggactggtattggtgggcgacttgactaatccgccctaccacgaattcgcatatgaagggctaaaaatccgccctgcctgcccatacaaaattgcagtcataggagtcttcggagtaccaggatctggcaagtcagctattatcaagaacctagttaccaggcaagacctggtgactagcggaaagaaagaaaactgccaagaaatcaccaccgacgtgatgagacagagaggtctagagatatctgcacgtacggttgactcgctgctcttgaatggatgcaacaggccagtcgacgtgttgtacgtagacgaggcgtttgcgtgccactctggaacgttacttgctttgatcgccttggttagaccaaggcagaaagttgtactctgtggtgacccgaagcagtgcggcttcttcaatatgatgcagatgaaagtcaactataatcacaacatctgcacccaagtgtaccacaaaagtatctccaggcggtgtacactgcctgtgaccgccattgtgtcatcgttacattacgaaggcaaaatgcgcactacgaatgagtacaacaagccgattgtagtggacactacaggctcaacaaaacctgacgctggagatctcgtgttaacgtgcttcagagggtgggttaaacaactgcaaattgactatcgtggatacgaggtcatgacagcagccgcatcccaagggttaaccagaaaaggagtttatgcagttaggcaaaaagttaatgaaaacccgctctatgcatcaacgtcagagcacgtcaacgtactcctaacgcgtacggaaggtaaactggtatggaagacactttccggtgacccgtggataaagacgctgcagaacccaccgaaaggaaacttcaaagcaactatcaaggagtgggaggtggagcatgcatcaataatggcgggcatctgcagtcaccaaatgacctttgatacattccaaaacaaagccaatgtttgttgggctaagagcttggtccctatcctcgaaacagcggggataaaactaaatgataggcagtggtcccagataattcaagccttcaaagaagataaagcatactcacctgaagtagccctgaatgaaatatgcacgcgcatgtatggggtggatctagacagtgggctattttctaaaccattggtgtctgtgtactactcggataaccactgggataataggcctggagggaagatgttcggattcaaccccgaggcagcatccattctagaaagaaagtatccattcacaaaagggaagtggaacatcaacaagcagatctgtgtgaccaccaggaggatagaagactttaaccctaccaccaatattataccggccaataggagactaccacactcattagtggccgaacaccgcccagtaaaaggggaaagaatggaatggctggttaataagataaacggccaccacgtgctcctggtcagtggctataatcttgtactgcctactaagagagtcacttgggtagcgccactaggtgtccgcggagcggactacacatacaacctagagttgggtctgccagcaacgcttggcaggtatgacctagtggtcataaacatccacacaccttttcgcatacaccattaccaacagtgcgtagaccacgcaatgaaactgcaaatgctcgggggtgactcattgagactgctcaaaccgggtggctctctattgatcagagnnnnnnnnnnnnnnnntagaaccagtgaacgagtcatctgcgtgttgggacgcaagttcagatcgtctagagcgttgaaaccaccatgtgtcaccagcaacactgagatgtttttcctattcagcaactttgacaatggcagaaggaatttcacaactcatgtcatgaataatcaactgaatgcagcctttgtaggacaggccacccgagcaggatgtgcaccgtcgtaccgggtaaaacgcatggacatcgcgaagaacgatgaagagtgcgtagttaacgccgctaaccctcgcgggttaccgggtgacggtgtttgcaaggcagtatacaaaaaatggccggagtcctttaagaatagtgcaacaccagtgggaaccgcaaaaacagtcatgtgcggtacgtatccagtaatccacgccgttggaccgaatttctctaattatttggaatctgaaggggaccgggaattggcagctgcctatcgagaagtcgcaaaggaagtaactagactgggagtaaatagtgtagctatacctctcctctccacaggtgtannnnnnnnnnnnnnnnnnnnnnnnnnnnnnnnnnnnnnnnnnnnnnnnnnnnnnnnnnnnnnnnnnnnnnnnnnnnnnnnnnnnnnnnnnnnnnnnnnnnnnnnnnnnnagaaaatatccgaggccatacagatgcggacncaagtggagttgctggatgagcacatctccatagactgcgatattgttcgcgtgcaccctgacagcagcttggcaggcagaaaaggatacagcaccacggaaggcgcactgtactcatatctagaagggactcgttttcaccagacggcagtggacatggcagagatatatactatgtggccaaagcaaacagaggccaacgagcaagtctgcctatatgccctgggggaaagtattgaatcgatcaggcagaaatgcccggtggatgatgcagacgcatcgtctcctccaaaaactgtcccgtgcctttgccgttacgccatgactccggaacgcgtcaccagacttcgcatgaaccacgtcacaagtataattgtgtgttcctcgtttccccttccaaagtacaaaatagaaggagtgcaaaaagtcaaatgctctaaggtaatgctatttgaccacaacgtgccatcacgcgtaagtccaagggaatatagatcttccccggaggctgcacaggaagcgagtacaaccacgtcactgacgcatagccaattcgacctaagcgttgatggcgagatactacccgtcccgtcagatctggatgctgacgccccagccctagaaccagctctagacgacagggcgacacacacgttgccatccacaaccggaaaccttgcggccgtgtctgactgggtaatgagcaccgtacctgtcgcgccgcccagaagaaggcgagggagaaacctgactgtgacatgcgacgagagagaagggaacataacacccatggctagcgtccgattctttagggcagagctgcgtccggtcgtacaagaaacagcggagacgcgtgacacagcaatgtctcttcaggcaccgccgagtaccgccacggaactgaatcatccgccgatctccttcggagcaccaagcgagacgttccccattacatttggggacttcaacgaaggagaaatcgaaagcttgtcttctgagctgctaactttcggagacttcctacccggagaagtggatgacttgacagatagcgactggtccacgtgctcagacacggacgacgagttactagacagggcaggtgggtatatattctcgtcggacaccggtccaggtcatttacaacagaagtcagtacgccagtcagtgctgccggtgaacaccctggaggaagtccacgaggagaagtgttacccacctaagttggatgaagtaaaggagcaactattacttaagaaactccaggagagtgcatccatggccaacagaagcaggtaccagtcgcgcaaagtagaaaacatgaaagcaacaatcatccagagactaaagagaggctgtagactatacttaatgtcagagacctcaaaagtccctacttaccggaccacatatccggcgcctgtgtactcgcctccggtcaacgtccgactgtccaaccccgagtccgcagtggcagcatgcaatgagttcctagctagaaactatccaactgtctcatcataccaaattaccgacgagtatgatgcatatttagacatggtggacgggtcggagagttgcctggatcgagcgacattcaatccgtcaaaacttaggagctacccgaaacagcacgcttaccatgcgccctccatcagaagcgctgtaccgtccccattccagaacacactacagaatgtactggcagcagccacgaaaagaaactgcaacgtcacacagatgagggaattacccactttggactcagcagtattcaacgtggagtgttttaaaaaattcgcatgcaaccaagaatactgggaagaatttgctgccagccctattaggataacaactgagaatttaacaacctatgttactaaactaaaagggccaaaagcagcagcgctatttgcnaaaacccacaatctgctgccactgcaggangtaccaatggataggttcacagtagatatgaaaagggacgtgaaggtgactcctggtacaaagcacacagaggaaagacctaaggtgcaggttatacaggcggctgaacccttggcgacagcatacctatgtgggattcacagagagctggttaggaggctgaacgccgtcctcctacccaatgtacatacactatttgatatgtctgccgaggatttcgatgccatcatagccgcacactttaagccaggagacactgttttagaaacggacatagcctcctttgacaagagccaagatgattcacttgcgcttactgccttgatgttgttagaagatttaggggtggatcactccctgttggacttgatagaggccgcttttggagagatttccagctgtcatctaccgacaggtacgcgcttcaagttcggcgccatgatgaaatctggtatgttcctaactctgttcgtcaacacattgctaaacatcaccatcgctagccgcgtgctggaagatcgtctgacaaaatccgcgtgcgcggccttcatcggcgacgacaacataatacatggagtcgtctccgatgaattgatggcagacagatgcgccacttggatgaacatggaagtgaagatcatagatgcagttgtatccttgaaagccccttacttttgtggagggtttatactgcacgatactgtgacgggaacagcctgcagagtggcagacccgctaaaaaggctattcaaactgggcaaaccgctagcggcaggtgacgaacaagatgaagatagaagacgagcgctggctgacgaagtgatcagatggcaacgaacagggctaattgatgagttggagaaagcggtatactctaggtatgaagtgcagggtatatcagttgtggtaatgtccatggccacctttgcaagctccagatctaacttcgagaagctcagaggaccccagttaataaactgacaatgcgcgcggtaccccaacagaagccacgcaggaatcggaagaataagaagcaaaagcaaaagcgacaggcgccacaaaacaacacaaatcaaaagaagcagccgcctaaaaagaaaccagctcaaaagaaaaagaagccgggccgcagagagaggatgtgcatgaaaatcgaaaatgattgtatttttgaagtcaagtatgaaggtaaggtaacaggttacgcgtgcttggtgggggacaaagtaatgaaaccagcacacgtaaaggggaccatcgataacgcggacctggccaaactggcctttaagcggtcatctaagtatgaccttgaatgcgcgcagatacccgtgcacatgaagtccgacgcttcgaagttcacccatgagaaaccagaggggtactacaactggcaccacggagcagtacagtactcaggaggccggttcaccatccccacaggtgctggcaaaccaggggacagcggcagaccgatcttcgacaacaagggacgcgtggtggccatagtcttaggaggagctaatgaaggagcccgtacagccctctcggtggtgacctggaataaagacattgtcactaaaatcacccccgagggggccgaagagtggagtcttgccatcccagtcatgtgcctgctggcaaataccacgttcccctgctcccagcccccttgcactccctgctgctatgaaagggaaccagaggaaaccctacgcatgcttgaggacaacgtcatgagacctgggtactatcagctgctacaagcattaaagacggatgatagccacgattggaccaagctgcgttacatggacaaccacacgccagcggacgcagagagggcggggctatttgtaagaacatcagcaccgtgcacgattactggaacaatgggacacttcatcctgacccgatgtccgaaaggggaaactctgacggtgggattcactgacagtaggaagatcagtcactcatgtacgcacccatttcaccacgaccctcctgtgataggccgggagaaattccattcccgaccgcagcacggtaaaganctnccttgcagcacgtacgtgcagagcaccgccgcaactaccgaggagatagaggtacacatgcccccagacacccctgatcgcacactgatgtcacaacagtccggcaacgtaaagatcacagttaatggccagacggtgcggtacaagtgtaattgcggtggctcaaatgaaggactaataactacagacaaagtgattaataactgcaaagttgatcaatgtcatgccgcggtcaccaatcacaaaaagtggcagtacaactcccctctggtcccgcgtaatgctgaacttggggaccgaaaaggaaaaatccacatcccgtttccgctggcaaatgtaacatgcagggtgcctaaagcaaggaaccccaccgtgacgtacgggaaaaaccaagtcatcatgctactgtatcccgaccacccaacactcctgtcctaccggaacatgggagaagaaccaaactaccaagaagagtgggtgacgcataagaaggaagtcgtgctaaccgtgccgactgaagggctcgaggtcacgtggggtaacaacgagccgtataagtattggccgcagttatctacaaacggtacagcccatggccacccgcatgagataattctgtattattatgagctgtaccctactatgactgtagtagttgtgtcagtggcctcgttcgtactcctgtcgatggtgggtgtggcagtggggatgtgcatgtgtgcacgacgcagatgcannncaccntacgaactgacaccaggagctaccgtccctttcctgcttagcctaatatgctgcatcagaacagctaaagcggccacataccaagaggctgcggtatacctgtggaatgaacagcaacctttgttttggctacaagcccttattccgctggcagccctgattgtcctgtgcaactgtctgagactcttaccatgctgctgtaaaacgttggcttttttagccgtaatgagcgtcggtgcccacactgtgagcgcgtacgaacacgtaacagtgatcccgaacacggtgggagtaccgtataagactctagtcaatagaccgggctacagtcccatggtattggagatggaactactgtcagtcactttggagccaacgctatcgcttgattacatcacgtgcgagtacaaaaccgttatcccgtctccgtacgtgaaatgctgcggtacagcagagtgcaaggacaaaaacctacctgactacagctgtaaggtcttcaccggcgtctacccatttatgtggggcggagcctgccaaattcattgtggggccaatgtcttcagcctggacacctttcgacaacaaaattgtggtgtacaaaggtgacgtctataacatggactacccgccctttggcgcaggaagaccaggacaatttggcgatatccaaagtcgcacacctgagagtacagacgtctatgctaatacacaactggtactgcagagaccggctgcgggtacggtacatgtgccatactctcaggcaccatctggctttaagtattggctaaaagaacgaggggcgtcgctgcagcacacagcaccatttggctgccaaatagcaacaaacccggtaagagcggtgaattgcgccgtagggaacatgcccatctccatcgacataccgga

>MK156064

cgtctgcccgatgcgcagcgcagaagatcccgagagactcgccaattatgcgagaaagctagcatctgctgcaggaaaagtcctggacagaaacatctctggaaagatcggggacttacaagcagtaatggccgtgccagacacggagacgccaacattctgcttgcacacagatgtctcatgtagacagagagcagacgtcgcgatataccaagacgtctatgctgtacacgcacccacgtcgctataccaccaggcgattaaaggggtccgagtggcgtactgggtagggttcgacacaaccccgttcatgtacaatgccatggcgggtgcctacccctcatattcgacaaactgggcagacgagcaggtactgaaggctaagaacataggattatgttcaacagacctgacggaaggtagacgaggtaaattgtctattatgagagggaagaagctaaaaccgtgtgaccgtgtgctgttctcagtagggtcaacgctctacccggaaagccgcaagctacttaagagctggcacttaccatcggtgttccatctaaagggcaaactcagcttcacatgccgctgtgatacagtggtttcgtgcgagggctacgtcgttaagagaataacgatgagcccaggcctttatggaaaaaccacagggtatgcggtaacccaccacgcagacggattcttgatgtgcaagactaccgacacggttgacggcgaaagagtgtcattctcggtgtgcacatacgtgccggcgaccatttgtgatcaaatgaccggcatccttgctacagaagtcacgccggaggatgcacagaagctgttggtggggctgaaccagagaatagtggttaacggcagaacgcaacggaatacgaacaccatgaaaaactatctgcttcccgtggtcgcccaagccttcagtaagtgggcaaaggagtgccggaaagacatggaagatgaaaaactcctgggggtcagagaaagaacactgacctgctgctgtctatgggcattcaagaagcagaaaacacacacggtctacaagaggcctgatacccagtcaattcagaaggttcaggccgagtttgacagctttgtggtaccgagcctgtggtcgtccgggttgtcaatccccttgaggactagaatcaaatggttgttaagcaaggtgccaaaaaccgacctgatcccatatagcggggacgcccaagaagcccgggacgcagaaaaagaagcagaggaagaacgagaagcannnnnnnnnnnnnnnnnnnnnnnnnnnnnnnnnnnnnnnnnnnnnnnnnnnnnnnnnnnnnnnnnnnnggaacagcttgaggacagagcgggtgcaggaataatagagactcctagaggagctatcaaagttactgcccaaccaacagaccacgtcgtgggagagtacctggtactttccccgcagaccgtactacggagccagaagctcagcctaattcacgctttggcggagcaagtgaagacgtgcacncacagcggacgagcagggaggtacgcggtcgaagcgtacgacggccgagtcctagtgccctcaggctatgcaatctcgcctgaagacttccagagtctaagcgaaagcgcaacgatggtgtataacgaaagagagttcgtaaacagaaagctacaccacattgcgatgcacggaccagccctgaacaccgacgaagagtcgtatgagctggtgagggcagagaggacagaacacgagtacgtctacgatgtggaccagagaagatgctgtaagaaggaagaagctgcaggactggtattggtgggcgacttgactaatccgccctaccacgaattcgcatatgaagggctaaaaatccgccctgcctgcccatacaaaattgcagtcataggagtcttcggagtaccaggatctggcaagtcagctattatcaagaacctagttaccaggcaagacctggtgactagcggaaagaaagaaaactgccaagaaatcaccaccgacgtgatgagacagagaggtctagagatatctgcacgtacggttgactcgctgctcttgaatggatgcaacaggccagtcgacgtgttgtacgtagacgaggcgtttgcgtgccactctggaacgttacttgctttgatcgccttggttagaccaaggcagaaagttgtactctgtggtgacccgaagcagtgcggcttcttcaatatgatgcagatgaaagtcaactataatcacaacatctgcacccaagtgtaccacaaaagtatctccaggcggtgtacactgcctgtgaccgccattgtgtcatcgttacattacgaaggcaaaatgcgcactacgaatgagtacaacaagccgattgtagtggacactacaggctcaacaaaacctgacgctggagatctcgtgttaacgtgcttcagagggtgggttaaacaactgcaaattgactatcgtggatacgaggtcatgacagcagccgcatcccaagggttaaccagaaaaggagtttatgcagttaggcaaaaagttaatgaaaacccgctctatgcatcaacgtcagagcacgtcaacgtactcctaacncgtacggaaggtaaactggtatggaagacactttccggtgacccgtggataaagacgctgcagaacccaccgaaaggaaacttcaaagcaactatcaaggagtgggaggtggagcatgcatcaataatggcgggcatctgcagtcaccaaatgacctttgatacattccaaaacaaagccaatgtttgttgggctaagagcttggtccctatcctcgaaacagcggggataaaactaaatgataggcagtggtcccagataattcaagccttcaaagaagataaagcatactcacctgaagtagccctgaatgaaatatgcacgcgcatgtatggggtggatttagacagtgggctattttctaaaccattggtgtctgtgtactactcggataaccactgggataataggcctggagggaagatgttcggattcaaccccgaggcagcatccattctagaaagaaagtatccattcacaaaagggaagtggaacatcaacaagcagatctgtgtgaccaccaggaggatagaagactttaaccctaccaccaatattataccggccaataggagactaccacactcattagtggccgaacaccgcccagtaaaaggggaaagaatggaatggctggttaataagataaacggccaccacgtgctcctggtcagtggctataatcttgtactgcctactaagagagtcacttgggtagcgccactaggtgtccgcggagcggactacacatacaacctagagttgggtctgccagcaacgcttggcaggtatgacctagtggtcataaacatccacacaccttttcgcatacaccattaccaacagtgcgtagaccacgcaatgaaactgcaaatgctcgggggtgactcattgagactgctcaaaccgggtggctctctattgannnnnnnnnnnnnnnnnnnnnntagaaccagtgaacgagtcatctgcgtgttgggacgcaagttcagatcgtctagagcgttgaaaccaccatgtgtcaccagcaacactgagatgtttttcctattcagcaactttgacaatggcagaaggaatttcacaactcatgtcatgaataatcaactgaatgcagcctttgtaggacaggccacccgagcaggatgtgcaccgtcgtaccgggtaaaacgcatggacatcgcgaagaacgatnnngngnnnnnnnnnnnnnnnnnnnnnnnnnnnnnnnnnnnnnnnnnnnnnnnnnnnnnnnnnnnnnacaaaaaatggccggagtcctttaagaatagtgcaacaccagtgggaaccgcaaaaacagtcatgtgcggtacgtatccagtaatccacgccgttggaccgaatttctctaattatttggaatctgaaggggaccgggaattggcagctgcctatcgagaagtcgcaaaggaagtaactagactgggagtaaatagtgtagctatacctctcctctccacaggtgnnnnnnnnnnnnnnnnnnnnnnnnnnnnnnnnnnnnnnnnnnnnnnnnnnnnnnnnnnnnnnnnnnnnnnnnnnnnnnnnnnnnnnnnnnnnnnnnnnnnnnnnnnnnnnnnnnnnnnnnnnnnnnnnatacagatgcggactcaagtggagttgctggatgagcacatctccatagactgcgatattgttcgcgtgcaccctgacagcagcttggcaggcagaaaaggatacagcaccacggaaggcgcactgtactcatatctagannnnnnnnnnnnnnnnnnnnnggcagtggacatggcagagatatatactatgtggccaaagcaaacagaggccaacgagcaagtctgcctatatgccctgggggaaagtattgaatcgatcaggcagaaatgcccggtggatgatgcagacgcatcgtctcctccaaaaactgtcccgtgcctttgccgttacgccatgactccggaacgcgtcaccngacttcgcatgaaccangtcacaagtataattgtgtgttcctcgtttccccttccaaagtacaaaatagaaggagtgcaaaaagtcaaatgctctaaggtaatgctatttgaccacaacgtgccatcacgcgtaagtccaagggaatatagatcttccccggaggctgcacaggaagcgagtacaaccacgtcactgacgcatagccaattcgacctaagcgttganggcgagatactacccgtcccgtcagatctngatgctgacgccccagccctagaaccagctcnnnnnnnnnnnnnnnnnnnnnnnnnnnnnnnnnnnnnnnnnnnnnnnnnnnnnnnnnnnnnnnnnnnnnnnnnnnnnnnnnnnnnnnnnnnnnnnnnnnnnnnnnnnnnnnnnnnnnnnnnnnnnnnnnnnnnnnnnnnnnnnnnnnnnnnnnnnnnnnnnnnnnnnnnnnnnnnnnnnnnnnnnnnnnnnnnnnnnnnnnnnnnnnnnnnnnnnnnnnnnnnnnnnnnnnnnnnnnnnnnncaggcaccgccgagtaccgccacggaactgaatcatccgccgatctccttcggagcaccaagcgagacgttccccattacatttggggacttcaacgaaggagaaatcgaaagcttgtcttctgagctgctaactttcggagacttcctacccggagaagtggatgacttgacagatagcgactggtccacgtgctcagacacggacgacgagttactagacagggcaggtgggtatatattctcgtcggacaccggtccaggtcatttacaacagaagtcagtacgccagtcagtgctgccggtgaacaccctggaggaagtccacgaggagaagtgttacccacctaagttggatgaagtaaaggagcaactattacttaagaaactccaggagagtgcatccatggccaacagaagcaggtaccagtcgcgcaaagtagaaaacatgaaagcaacaatcatccagagactaaagagaggctgtagactatacttaatgtcagagacctcaaaagtccctacttaccggaccacatatccggcgcctgtgtactcgcctccggtcaacgtccgactgtccaaccccgagtccgcagtggcagcatgcaatgagttcctagctagaaactatccaactgtctcatcataccaaattaccgacgagtatgatgcatatttagacatggtggacgggtcggagagttgcctggatcgagcgacattcaatccgtcaaaacttaggagctacccgaaacagcacgcttaccatgcgccctccatcagaagcgctgtaccgtccccattccagaacacactacagaatgtactggcagcagccacgaaaagaaactgcaacgtcacacagatgagggaattacccactttggactcagcagtattcaacgtggagtgttttaaaaaattcgcatgcaaccaagaatactgggaagaatttgctgccagccctattaggataacaactgagaatttaacaacctatgttactaaactaaaagggccaaaagcagcagcgctatttgcnnnnnnnnnnnnnnnnnnnccactgcaggaagtaccaatggataggttcacagtagatatgaaaagggacgtgaaggtgactcctggtacaaagcacacagaggaaagacctaaggtgcaggttatacaggcggctgaacccttggcgacagcatacctatgtgggattcacagagagctggttaggaggctgaacgccgtcctcctacccaatgtacatacactatttgatatgtctgccgaggatttcgatgccatcatagccgcacactttaagccaggagacactgttttagaaacggacatagcctcctttgacaagagccaagatgattcacttgcgcttactgccttgatgttgttagaagatttaggggtggatcactccctgttggacttgatagaggccgcttttggagagatttccagctgtcannnnnnnnnnnnnnnnnnnnnnnnnnnnggcgccatgatgaaatctggtatgttcctaactctgttcgtcaacacattgctaaacatcaccatcgctagccgcgtgctggaagatcgtctgacaaaatccgcgtgcgcggccttcatcggcgacgacaacataatacatggagtcgtctccgatgaattgatggcagacagatgcgccacttggatgaacatggaagtgaagatcatagatgcagttgtatccttgaaagccccttacttttgtggagggtttatactgcacgatactgtgacgggaacagcctgcagagtggcagacccgctaaaaaggctattcaaactgggcaaaccgctagcggcaggtgacgaacaagatgaagatagaagacgagcgctggctgacgaagtgatcagatggcaacgaacagggctaattgatgagttggagaaagcggtatactctaggtatgaagtgcagggtatatcagttgtggtaatgtccatggccacctttgcaagctccagatctaacttcgagaagctcagaggacccnnnnnnnnnnnnnnnnnnnnnnnnnnnnnnnnnnnnnnnnnnnnnnnnnnnnnnnnnnnnnnnnnnnnnnnnnnnnnnnnnnnnnnnnnnnnnnnnnnnnnnnnnnnnnnnnnnnnnnnnnnnnnnnnnnnnnnnnnnnnnnnnnnnnnnnnnnnnnnnnnnnnnnnnnnnnnnnnnnnnnnnnnnnnnnnnnnnnnnnnnnnnnnnnnnnnnnnnnnnnnnnnnnnnnnnnnnnnnnnnnnnnnttggtgggggacaaagtaatgaaaccagcacacgtaaaggggaccatcgataacgcggacctggccaaactggcctttaagcggtcatctaagtatgaccttgaatgcgcgcagatacccgtgcacatgaagtccgacgcttcgaagttcacccatgagaaaccagaggggtactacaactggcaccacggagcagtacagtactcaggaggccggttcaccatccccacaggtgctggcaaaccaggggacagcggcagaccgatcttcgacaacaagggacgcgtggtggccatagtcttaggaggagctaatgaaggagcccgtacagccctctcggtggtgacctggaataaagacattgtcactaaaatcacccccgagggggccgaagagtggagtcttgccatcccagtcatgtgcctgctggcaaataccacgttcccctgctcccagcccccttgcactccctgctgctatgaaagggaaccagaggaaaccctacgcatgcttgaggacaacgtcatgagacctgggtactatcagctgctacaagcattaaagacggatgatagccacgattggaccaagctgcgttacatggacaaccacacgccagcggacgcagagagggcggggctatttgtaagaacatcagcaccgtgcacgattactggaacaatgggacacttcatcctgacccgatgtccgaaaggggaaactctgacggtgggattcactgacagtaggaagatcagtcactcatgtacgcacccatttcaccacgaccctcctgtgataggccgggagaaattccattcccgaccgcagcacggtaaagagctgccttgcagcacgtacgtgcagagcaccgccgcaactaccgaggagatagaggtacacatgcccccagacacccctgatcgcacactgatgtcacaacagtccggcaacgtaaagatcacagttaatggccagacggtgcggtacaagtgtaattgcggtggctcaaatgaaggactaataactacagacaaagtgattaataactgcaaagttgatcaatgtcatgccgcggtcaccaatcacaaaaagtggcagtacaactcccctctggtcccgcgtaatgctgaacttggggaccgaaaaggaaaaatccacatcccgtttccgctggcaaatgtaacatgcagggtgcctaaagcaaggaaccccaccgtgacgtacgggaaaaaccaagtcatcatgctactgtatcccgaccacccaacactcctgtcctaccggaacatgggagaagaaccaaactaccaagaagagtgggtgacgcataagaaggaagtcgtgctaaccgtgccgactgaagggctcgaggtcacgtggggtaacaacgagccgtataagtattggccgcagttatctacaaacggtacagcccatggccacccgcatgagataattctgtattattatgagctgtaccctactatgactgtagtagttgtgtcagtggcctcgttcgtactcctgtcgatggtgggtgtggcagtggggatgtgcatgtgtgcacgacgcagatgcannncaccntacgaactgacaccaggagctaccgtccctttcctgcttagcctaatatgctgcatcagaacagctaaagcggccacataccaagaggctgcggtatacctgtggaatgaacagcaacctttgttttggctacaagcccttattccgctggcagccctgattgtcctgtgcaactgtctgagactcttaccatgctgctgtaaaacgttggcntttttagccgtaatgagcgtcggtgcccacactgtgagcncntacgaacacgtaacagtgatcccgaacacggtgagagtaccgtataagactctagtcaatagaccgggctacagtcccatggtattggagatggaactactgtcagtcactttggagccaacgctatcgcttgattacatcacgtgcgagtacaaaaccgttatcccgtctccgtacgtgaaatgctgcggtacagcagagtgcaaggacaaaaacctacctgactacagctgtaaggtcttcaccggcgtctacccatttatgtggggcggagcctgccaaattcattgtggggccaatgtcttcagcctggacacctttcgacaacaaaattgtggtgtacnaaggtnnnnnnnnnnnnnnnnnnnnnnnnnnnnnnnnnnnnnnnnnnnnnnnnnnnnnnnnnnnnnnnnnnnnnnnnnnnnnnnnnnnnnnnnnnnnnnnnnnnnnnnnnnnnnnnnnnnnnnnnnnnnnnnnnnnnnnnnnnnnnnnnnnnnnnnnnnnnnnnnnnnnnnnnnnnnnnnnnnnnnnnnnnnnnnnnnnnnnnnnnnnnnnnnnnnnnnnnnnnnnnnnnnnnnnnnnnnnnnnnnnnnnnnnnnnnnnnnnnnnnnnnnnnnnnnnnnnnnnnnnnnnnnnnnnnnnnnnnnnnnnnnn

>MK752950

cgtctgcccgatgcgcagcgcagaagatcccgagagactcgccaattatgcgagaaagctagcatctgctgcaggaaaagtcctggacagaaacatctctggaaagatcggggacttacaagcagtaatggccgtgccagacacggagacgccaacattctgcttgcacacagatgtctcatgtagacagagagcagacgtcgcgatataccaagacgtctatgctgtacacgcacccacgtcgctataccaccaggcgattaaaggggtccgagtggcgtactgggtagggttcgacacaaccccgttcatgtacaatgccatggcgggtgcctacccctcatattcgacaaactgggcagacgagcaggtactgaaggctaagaacataggattatgttcaacagacctgacggaaggtagacgaggtaaattgtctattatgagagggaagaagctaaaaccgtgtgaccgtgtgctgttctcagtagggtcaacgctctacccggaaagccgcaagctacttaagagctggcacttaccatcggtgttccatctaaagggcaaactcagcttcacatgccgctgtgatacagtggtttcgtgcgagggctacgtcgttaagagaataacgatgagcccaggcctttatggaaaaaccacagggtatgcggtaacccaccacgcagacggattcttgatgtgcaagactaccgacacggttgacggcgaaagagtgtcattctcggtgtgcacatacgtgccggcgaccatttgtgatcaaatgaccggcatccttgctacagaagtcacgccggaggatgcacagaagctgttggtggggctgaaccagagaatagtggttaacggcagaacgcaacggaatacgaacaccatgaaaaactatctgcttcccgtggtcgcccaagccttcagtaagtgggcaaaggagtgccggaaagacatggaagatgaaaaactcctgggggtcagagaaagaacactgacctgctgctgtctatgggcattcaagaagcagaaaacacacacggtctacaagaggcctgatacccagtcaattcagaaggttcaggccgagtttgacagctttgtggtaccgagcctgtggtcgtccgggttgtcaatccccttgaggactagaatcaaatggttgttaagcaaggtgccaaaaaccgacctgatcccatatagcggggacgcccaagaagcccgggacgcagaaaaagaagcagaggaagaacgagaagcagnnnnnnnnnnnnnnnnnnnnnnnnnnnnnnnnnnnnnnnnnnnnnnnnnnnnnnnnnnnnnnnnnnnnnnnnagcttgaggacagagcgggtgcaggaataatagagactcctagaggagctatcaaagttactgcccaaccaacagaccacgtcgtgggagagtacctggtactttccccgcagaccgtactacggagccagaagctcagcctaattcacgctttggcggagcaagtgaagacgtgcnnncacagcggacgagcagggaggtacgcggtcgaagcgtacgacggccgagtcctagtgccctcaggctatgcaatctcgcctgaagacttccagagtctaagcgaaagcgcaacgatggtgtataacgaaagagagttcgtaaacagaaagctacaccacattgcgatgcacggaccagccctgaacaccgacgaagagtcgtatgagctggtgagggcagagaggacagaacacgagtacgtctacgatgtggaccagagaagatgctgtaagaaggaagaagctgcaggactggtactggtgggcgacttgactaatccgccctaccacgaattcgcatatgaagggctaaaaatccgccctgcctgcccatacaaaattgcagtcataggagtcttcggagtaccaggatctggcaagtcagctattatcaagaacctagttaccaggcaagacctggtgactagcggaaagaaagaaaactgccaagaaatcaccaccgacgtgatgagacagagaggtctagagatatctgcacgtacggttgactcgctgctcttgaatggatgcaacaggccagtcgacgtgttgtacgtagacgaggcgtttgcgtgccactctggaannnnnnnnnnnnnnnnnnnnnnnnnnnnnnnnnnnnnnnnnnnnnnnnnnnnnnnnnnnnnnnnnnnnnnnnnnnnnnnnnnnnnnnnnnnnnnnnnnnnnnnnnnnnnnnnnnnnnnnnnnnnnnnnnnnnnnnnnnnnnnnnnnnnnnnnnnnnnnnnnnnnnnnnnnnnnnnnnnnnnnnnnnnnnnnnnnnnnnnnnnnnnnnnnnnnnnnnnnnnnnagtacaacaagccgattgtagtggacactacaggctcaacaaaacctgacgctggagatctcgtgttaacgtgcttcagagggtgggttaaacaactgcaaattgactatcgtggatacgaggtcatgacagcagccgcatcccaagggttaaccagaaaaggagtttatgcagttaggcaaaaagttaatgaaaacccgctctatgcatcaacgtcagagcacgtcaacgnnnnnnnnnnnnnnnnnnnnnnnnnnnnnnnnnnnnnnnnnnnnnnnnnnnnnnnnnnnnnnnnnnnnnnnnnnnnnnnnnnnnnnnnnnnnnnnnnnnnnnnnnnnnnnnnnnnnnnnnnnnnnnnnnnnnnnnnnnnnnnnnnnnnnnnnnnnnnnnnnnnnnnnnnnnnnnnnnnnnnnnnnnnnnnnnnnnnnnnnnnnnnnnnnnnnnnnnnnnnnnnnnnnnnnnnnnnnnnnnnnnnnnnnnnnnntaggcagtggtcccagataattcaagccttcaaagaagataaagcatactcacctgaagtagccctgaatgaaatatgcacgcgcatgtatggggtggatttagacagtgggctattttctaaaccattggtgtctgtgtattacgcggataaccactgggataataggcctggagggaagatgttcggattcaaccccgaggcagcatccattctagaaagaaagtatccattcacaaaagggaagtggaacatcaacaagnnnnnnnnnnnnnnnnnnnnnnnnnnnnnnnnnnnnnnnnnnnnnnnnnnnnnnnnnnnnnnnnnnnnnnnnnnnnnnnnnnnnnnnnnnnnnnnnnnnnnnnnnnnnnnnnnnnnnnnnnnnnnnnnnnnnnnnnnnnnnnnnnnnnnnnnnnnnnnnnnnnnnnnnnnnnnnnnnnnnnnnnnnnnnnnnnnnnnnnnnnnnnnnnnnnnnnnnnnnnnnnnnnnnnnnnnnnnnnnnnnnnnnnnnnnnnnnnnnnnnnnnnnnnnnnnnnnnnnnnnnnnnnnnnnnnnnnnnnnnnnnnnnnnnnnnnnnnnnnnnnnnnnnnnnnnnnnnnnnnnnnnnnnnnnnnnnnnnnnnnnnnnnnnnnnnnnnnnnnnnnnnnnnnnnnnnnnnnnnnnnnnnnnnnnnnnnnnnnnnnnnnnnnnnnnnnnnnnnnnnnntagaaccagtgaacgagtcatctgcgtgttgggacgcaagttcagatcgtctagagcgttgaaaccaccatgtgtcaccagcaacactgagatgtttttcctattcagcaactttgacaatggcagaaggaatttcacaactcatgtcatgaataatcaactgaatgcagcctttgtaggacaggccacccgagcaggatgtgcaccgtcgtaccgggtaaaacgcatggacatcgcgaagaacgatnnagngtgcnnnnnnnnnnnnnnnnnnnnnnnnnnnnnnnnnnnnnnnnnnnnnnnnnnnnnnnnnnnnnnnnnnnnnnnnnnnnnnnnnnnnnnnnnnnnnnnnnnnnnnnnnnnnnnnnnnnnnnnnnnnnnnnnnnnnnnnnnnnnnnnnnnnnnnnnnnnnnnnnnnnnnnnnnnnnnnnnnnnnnnnnnnnnnnnnnnnnnnnnnnnnnnnnnnnnnnnnnnnnnnnnnnnnnnnnnnnnnnnnnnnnnnnnnnnnnnnnnnnnnnnnnnnnnnnnnnnnnnnnnnnnnnnnnnnnnnnnnnnnnnnnnnnnnnnnnnnnnnnnnnnnnnnnnnnnnnnnnnnnnnnnnnnnnnnnnnnnnnnnnnnnnnnnnnnnnnnnnnnnnnnnnnnnnnnnnnnnnnnnnnnnnnnnnnnnnnnnnnnnnnnnnnnnnnnnnnnnnnnnnnnnnnnnnnnnnnnnnnnnnnnnnnnnnnnnnnnnnnnnnnnnnnnnnnnnnnnnnnnnnnnnnnnnnnnnnnnnnnnnnnnnnnnnnnnnnnnnnnnnnnnnnnnnnnnnnnnnnnnnnnnnnnnnnnnnggcagtggacatggcagagatatatactatgtggccaaagcaaacagaggccaacgagcaagtctgcctatatgccctgggggaaagtattgaatcgatcaggcagaaatgcccggtggatgatgcagatgcatcgtctcctccaaaaactgtcccgtgcctttgccgttacgccatgactccggaacgcgtcacccgacttcgcatgaaccacgtcnnaagnnnnnnnnnnnnnnnnnnnnnnnnnnnnnnnnnnnnnnnnnnnnnnnnnnnnnnnnnnnnnnnnnnnnnnnnnnnnnnnnnnnnnnnnnnnnnnnnnnnnnnnnnnnnnnnnnnnnnnnnnnnnnnnnnnnnnnnnnnnnnnnnnnnnnnnnnnnnnnnnnnnnnnnnnnnnnnnnnnnnnnnnnnnnnnnnnnnnnnnnnnnnnnnnnnnnnnnnnnnnnnnnnnnnnnnnnnnnnnnnnnnnnnnnnnnnnnnnnnnnnnnnnnnnnnnnnnnnnnnnnnnnnnnnnnnnnnnnnnnnnnnnnnnnnnnnnnnnnnnnnnnnnnnnnnnnnnnnnnnnnnnnnnnnnnnnnnnnnnnnnnnnnnnnnnnnnnnnnnnnnnnnnnnnnnnnnnnnnnnnnnnnnnnnnnnnnnnnnnnnnnnnnnnnnnnnnnnnnnnnnnnnnnnnnnnnnnnnnnnnnnnnnnnnnnnnnnnnnnnnnnnnnnnnnnnnnnnnnnnnnnnnncaggcaccgccgagtaccgccacggaactgaatcatccgccgatctccttcggagcaccaagcgagacgttccccattacatttggggacttcaacgaaggagaaatcgaaagcttgtcttctgagctgctaactttcggagacttcctacccggagaagtggatgacttgacagatagcgactggtccacgtgctcagacacggacgacgagttactagacagggcaggtgggtatatattctcgtcggacaccggtccaggtcatttacaacagaagtcagtacgccagtcagtgctgccggtgaacaccctggaggaagtccacgaggagaagtgttacccacctaagttggatgaagtaaaggagcaactattacttaagaaactccaggagagtgcatccatggccaacagaagcaggtaccagtcgcgcaaagtagaaaacatgaaagcaacaatcatccagagactaaagagaggctgtagactatacttaatgtcagagacctcaaaagtccctacttaccggaccacatatccggcgcctgtgtactcgcctccgatcaacgtccgactgtccaaccccgagtccgcagcggcggcatgcaatgagttcctagctagaaactatccaactgtctcatcataccaaattaccgacgagtatgatgcatatttagacatggtggacgggtcggagagttgcctggatcgagcgacattcaatccgtcaaaacttaggagctacccgaaacagcacgcttaccatgcgccctccatcagaagcgctgtnnnnnnnnnnnnnnnnnnnnnnnnnnnnnnnnnnnnnnnnnnnnnnnnnnnnnnnnnnnnnnnnnnnnnnnnnnnnnnnnnnnnnnnnnnnnnnnnnnnnnnnnnnnnnnnnnnnnnnnnnnnnnnnnnnnnnnnnnnnnnnnnnnnnnnnnnnnnnnnnnnnnnnnnnnnnnnnnnnnnnnnnnnnnnnnnnnnnnnnnnnnnnnnnnnnnnnnnnnnnnnnnnnnnnnnnnnnnnnnnnnnnnnnnnnnnnnnnnnnnnnnnnnnnccactgcaggaagtaccaatggataggttcacagtagatatgaaaagggacgtgaaggtgactcctggtacaaagcacacagaggaaagacctaaggtgcaggttatacaggcggctgaacccttggcgacagcatacctatgtgggattcacagagagctggttaggaggctgaacgccgtcctcctacccaatgtacatacactatttgatatgtctgccgaggatttcgatgccatcatagccgcacactttaagccaggagacactgttttagaaacggacatagcctcctttgacaagagccaagatgattcacttgcgcttactgccttgatgttgttagaagatttaggggtggatcactccctgttggacttgatagaggccgcttttggagagatttccagctgtcannnnnnnnnnnnnnnnnnnnnnnnnnnnnnnnnnnnnnnnnnnnnnnnnnnnnnnnnnnnnnnnnnnnnnnnnnnnnnnnnnnnnnnnnnnnnnnnnnnnnnnnnnnnnnnnnnnnnnnnnnnnnnnnnnnnnnnnnnnnnnnnnnnnnnnnnnnnnnnnnnnnnnnnnnnnnnnnnnnnnnnnnnnnnnnnnnnnnnnnnnnnnnnnnnnnnnnnnnnnnnnnnnnnnnnnnnnnnnnnnnnnnnnnnnnnnnnnnnnnnnnnnnnnnnnnnnnnnnnnnttatactgcacgatactgtgacgggaacagcctgcagagtggcagacccgctaaaaaggctattcaaactgggcaaaccgctagcggcaggtgacgaacaagatgaagatagaagacgagcgctggctgacgaagtgatcagatggcaacgaacagggctaattgatgagttggagaaagcggtatactctaggtatgaagtgcagggtatatcagttgtggtaatgtccatggccacctttgcaagctccagatctaacttcgagaagctcagaggaccccagttaataaactgacaatgcgcgcggtaccccaacagaagccacgcaggaatcggaagaataagaagcaaaagcaaaagcgacaggcgccacaaaacaacacaaaccaaaagaagcagccgcctaaaaagaaaccagctcaaaagaaaaagaagccgggccgcagagagaggatgtgcatgaaaatcgaaaatgattgtatttttgaagtcaagtatgaaggtaaggtaacaggttacgcgtgcttggtgggggacaaagtaatgaaaccagcnnnnnnnnnnnnnnnnnnnnnnnnnnnnnnnnnnnnnnnnnnnnnnnnnnnnnnnnnnnnnnnnnnnnnnnnnnnnnnnnnnnnnnnnnnnnnnnnnnnnnnnnnnnnnnnnnnnnnnnnnnnnnnnnnnnnnnnnnnnnnnnnnnnnnnnnnnnnnnnnnnnnnnnnnnnnnnnnnnnnnnnnnnnnnnnnnnnnnnnnnnnnnnnnnnnnnnnnnnnnnnnnnnnnnnnnnnnnnnnnnnnnnnnnnnnnacgcgtggtggccatagtcttaggaggagctaatgaaggagcccgtacagccctctcggtggtgacctggaataaagacattgtcactaaaatcacccccgagggggccgaagagtggagtcttgccatcccagtcatgtgcctgctggcaaataccacgttcccctgctcccagcccccttgcactccctgctgctatgaaagggaaccagaggaaaccctacgcatgcttgaggacaacgtcatgagacctgggtactatcagctgctacaagcatnnnnnnnnnnnnnnnnccacgattggaccaagctgcgttacatggacaaccacacgccagcggacgcagagagggcggggctatttgtaagaacatcagcaccgtgcacgattactggaacaatgggacacttcatcctgacccgatgtccgaaaggggaaactctgacggtgggattcactgacagtaggaagatcagtcactcatgtacgcacccatttcaccatgaccctcctgtgataggccgggagaaattccattcccgaccgcagcacnnnnnngagctgccttgcagcacgtacgtgcagagcaccgccgcaactaccgaggagatagaggtacacatgcccccagacacccctgatcgcacattgatgtcacaacagtccggcaacgtaaagatcacagttaatggccagacggtgcggtacaagtgtaattgcggtggctcaaatgaaggactaataactacagacaaagtgattaataactgcaaagttgatcaatgtcatgccgcggtcaccaatcacaaaaagtggcagtacaactcccctctggtcccgcgtaatgctgaacttggggaccgaaaaggaaaaatccacatcccgtttccgctggcaaatgtaacatgcagggtgcctaaagcaaggaaccccaccgtgacgtacgggaaaaaccaagtcatcatgctactgtatcccgaccacccaacactcctgtcctaccggaacatgggagaagaaccaaactaccaagaagagtgggtgacacataagaaggaagtcgtgctaaccgtgccgactgaagggctcgaggtcacgtggggtaacaacgagccgtataagtattggccgcagttatctacaaacggtacagcccatggccacccgcatgagataattctgtattattatgagctgtaccctactatgactgtagtagttgtgtcagtggcctcgttcgtactcctgtcgatggtgggtgtggcagtggggatgtgcatgtgcgcacgacgcagatgcannnnnnnnnnnnnnnnnnnnnnnnnnnnnnnnnnnnnnnnnnnnnnnnnnnnnnnnnnnnnnnnnnnnnnnnnnnnnnnnnnnnnnnnnnnnnnnnnnnnnnnnnnnnnnnnnnnnnnnnnnnnnnnnnnnnnnnnnnnnnnnnnnnnnnnnnnnnnnnnnnnnnnnnnnnnnnnnnnnnnnnnnnnnnnnnnnnnnnnnnnnnnnnnnnnnnnnnnnnnnnnnnnnnnnnnnnnnnnnnnnnnnnnnnnnnnnnnnagcgcgtacgaacacgtaacagtgatcccgaacacggtgggagtaccgtataagactctagtcaatagaccgggctacagtcccatggtattggagatggaactactgtcagtcactttggagccaacgctatcgcttgattacatcacgtgcgagtacaaaaccgttatcccgtctccgtacgtgaaatgctgcggtacagcagagtgcaaggacaaaaacctacctgactacagctgtaaggtcttcaccggcgtctacccatttatgtggggcggagcctnnnnnnnnnnnnnnnnnnccaatgtcttcagcctggacacctttcgacaacaaaattgtggtgtacaaaggtgacgtctataacatggactacccgccctttggcgcaggaagaccaggacaatttggcgatatccaaagtcgcacacctgagagtaaagacgtctatgctaatacacaactggtactgcagagaccggctgcgggtacggtacatgtgccatactctcaggcaccatctggctttaagtattggctaaaagaacgaggggcgtcgctgcagcacacagcaccatttggctgccaaatagcaacaaacccggtaagagcggtgaattgcgccgtagggaacatgcccatctccatcgacataccgga

>MK752951

cgtctgcccgatgcgcagcgcagaagatcccgagagactcgccaattatgcgagaaagctagcatctgctgcaggaaaagtcctggacagaaacatctctggaaagatcggggacttacaagcagtaatggccgtgccagacacggagacgccaacattctgcttgcacacagatgtctcatgtagacagagagcagacgtcgcgatataccaagacgtctatgctgtacacgcacccacgtcgctataccaccaggcgattaaaggggtccgagtggcgtactgggtagggttcgacacaaccccgttcatgtacaatgccatggcgggtgcctacccctcatattcgacaaactgggcagacgagcaggtactgaaggctaagaacataggattatgttcaacagacctgacggaaggtagacgaggtaaattgtctattatgagagggaagaagctaaaaccgtgtgaccgtgtgctgttctcagtagggtcaacgctctacccggaaagccgcaagctacttaagagctggcacttaccatcggtgttccatctaaagggcaaactcagcttcacatgccgctgtgatacagtggtttcgtgcgagggctacgtcgttaagagaataacgatgagcccaggcctttatggaaaaaccacagggtatgcggtaacccaccacgcagacggattcttgatgtgcaagactaccgacacggttgacggcgaaagagtgtcattctcggtgtgcacatacgtgccggcgaccatttgtgatcaaatgaccggcatccttgctacagaagtcacgccggaggatgcacagaagctgttggtggggctgaaccagagaatagtggttaacggcagaacgcaacggaatacgaacaccatgaaaaactatctgcttcccgtggtcgcccaagccttcagtaagtgggcaaaggagtgccggaaagacatggaagatgaaaaactcctgggggtcagagaaagaacactgacctgctgctgtctatgggcattcaagaagcagaaaacacacacggtctacaagaggcctgatacccagtcaattcagaaggttcaggccgagtttgacagctttgtggtaccgagcctgtggtcgtccgggttgtcaatccccttgaggactagaatcaaatggttgttaagcaaggtgccaaaaaccgacctgatcccatatagcggggacgcccaagaagcccgggacgcagaaaaagaagcagaggaagaacgagaagcagaannnnnnnnnnnnnnnnnnnnnnnnnnnnnnnnnnnnnnnnnnnnnnnnnnnnnnnnnnnnnnnnnnnnnnagcttgaggacagagcgggtgcaggaataatagagactcctagaggagctatcaaagttactgcccaaccaacagaccacgtcgtgggagagtacctggtactttccccgcagaccgtactacggagccagaagctcagcctaattcacgctttggcggagcaagtgaagacgtgcnnncacagcggacgagcagggaggtacgcggtcgaagcgtacgacggccgagtcctagtgccctcaggctatgcaatctcgcctgaagacttccagagtctaagcgaaagcgcaacgatggtgtataacgaaagagagttcgtaaacagaaagctacaccacattgcgatgcacggaccagccctgaacaccgacgaagagtcgtatgagctggtgagggcagagaggacagaacacgagtacgtctacgatgtggaccagagaagatgctgtaagaaggaagaagctgcaggactggtactggtgggcgacttgactaatccgccctaccacgaattcgcatatgaagggctaaaaatccgccctgcctgcccatacaaaattgcagtcataggagtcttcggagtaccaggatctggcaagtcagctattatcaagaacctagttaccaggcaagacctggtgactagcggaaagaaagaaaactgccaagaaatcaccaccgacgtgatgagacagagaggtctagagatatctgcacgtacggttgactcgctgctcttgaatggatgcaacaggccagtcgacgtgttgtacgtagacgaggcgtttgcgtgccactctggaacgttacttgctttgatcgccttggttagaccaaggcagaaagttgtactctgtggtgacccgaagcagtgcggcttcttcaatatgatgcagatgaaagtcaactataatcacaacatctgcacccaagtgtaccacaaaagtatctccaggcggtgtacactgcctgtgaccgccattgtgtcatcgttgcattacgaaggcaaaatgcgcactacgaatgagtacaacaagccgattgtagtggacactacaggctcaacaaaacctgaccctggagatctcgtgttaacgtgcttcagagggtgggttaaacaactgcaaattgactatcgtggatacgaggtcatgacagcagccgcatcccaagggttaaccagaaaaggagtttatgcagttaggcaaaaagttaatgaaaacccgctctatgcatcaacgtcagagcacgtcaacgnnnnnnnnnnnnnnnnnnnnnnnnnnnnnnnnnnnnnnnnnnnnnnnnnnnnnnnnnnnnnnnnnnnnnnnnnnnnnnnnnnnnnnnnnnnnnnnnnnnnnnnnnnnnnnnnnnnnnnnnnnnnnnnnnnnnnnnnnnnnnnnnnnnnnnnnnnnnnnnnnnnnnnnnnnnnnnnnnnnnnnnnnnnnnnnnnnnnnnnnnnnnnnnnnnnnnnnnnnnnnnnnnnnnnnnnnnnnnnnnnnnnnnnnaatgataggcagtggtcccagataattcaagccttcaaagaagataaagcatactcacctgaagtagccctgaatgaaatatgcacgcgcatgtatggggtggatttagacagtgggctattttctaaaccattggtgtctgtgtattacgcggataatcactgggataataggcctggagggaagatgttcggattcaaccccgaggcagcatccattctagaaagaaagtatccattcacaaaagggaagtggaacatcaacaagnnnnnnnnnnnnnnnnnnnnnnnnnnnnnnnnnnnnnnnnnnnnnnnnnnnnnnnnnnnnnnnnnnnnnnnnnnnnnnnnnnnnnnnnnnnnnnnnnnnnnnnnnnnnnnnnnnnnnnnnnnnnnnnnnnnnnnnnnnnnnnnnnnnnnnnnnnnnnnnnnnnnnnnnnnnnnnnnnnnnnnnnnnnnnnnnnnnnnnnnnnnnnnnnnnnnnnnnnnnnnnnnnnnnnnnnnnnnnnnnnnnnnnnnnnnnnnnnnnnnnnnnnnnnnnnnnnnnnnnnnnnnnnnnnnnnnnnnnnnnnnnnnnnnnnnnnnnnnnnnnnnnnnnnnnnnnnnnnnnnnnnnnnnnnnnnnnnnnnnnnnnnnnnnnnnnnnnnnnnnnnnnnnnnnnnnnnnnnnnnnnnnnnnnnnnnnnnnnnnnnnnnnnnnnnnnnnnnnnnnnnntagaaccagtgaacgagtcatctgcgtgttgggacgcaagttcagatcgtctagagcgttgaaaccaccatgtgtcaccagcaacactgagatgtttttcctattcagcaactttgacaatggcagaaggaatttcacaactcatgtcatgaataatcaactgaatgcagcctttgtaggacaggccacccgagcaggatgtgcaccgtcgtaccgggtaaaacgcatggacatcgcgaagaacgatnnagngtgcnnnnnnnnnnnnnnnnnnnnnnnnnnnnnnnnnnnnnnnnnnnnnnnnnnnnnnnnnnnnnnnnnnnnnnnnnnnnnnnnnnnnnnnnnnnnnnnnnnnnnnnnnnnnnnnnnnnnnnnnnnnnnnnnnnnnnnnnnnnnnnnnnnnnnnnnnnnnnnnnnnnnnnnnnnnnnnnnnnnnnnnnnnnnnnnnnnnnnnnnnnnnnnnnnnnnnnnnnnnnnnnnnnnnnnnnnnnnnnnnnnnnnnnnnnnnnnnnnnnnnnnnnnnnnnnnnnnnnnnnnnnnnnnnnnnnnnnnnnnnnnnnnnnnnnnnnnnnnnnnnnnnnnnnnnnnnnnnnnnnnnnnnnnnnnnnnnnnnnnnnnnnnnnnnnnnnnnnnnnnnnnnnnnnnnnnnnnnnnnnnnnnnnnnnnnnnnnnnnnnnnnnnnnnnnnnnnnnnnnnnnnnnnnnnnnnnnnnnnnnnnnnnnnnnnnnnnnnnnnnnnnnnnnnnnnnnnnnnnnnnnnnnnnnnnnnnnnnnnnnnnnnnnnnnnnnnnnnnnnnnnnnnnnnnnnnnnnnnnnnnnnnnnnnnnnnggcagtggacatggcagagatatatactatgtggccaaagcaaacagaggccaacgagcaagtctgcctatatgccctgggggaaagtattgaatcgatcaggcagaaatgcccggtggatgatgcagacgcatcgtctcctccaaaaactgtcccgtgcctttgccgttacgccatgactccggaacgcgtcacccgacttcgcatgaaccacgtcacaagtataattgtgtgttccnngnnnnnncttnnnnagtacaaaatagaaggagngcnnaaagtcaaatgctctaaggtnnngctatttgaccacaacgtgccatcacncnnaagtccnagggaatatagatcnnncccngaggctgnacaggaagcgagtacaaccacgtcactgacgcatagccaattcgacctaagngttgacggcgagatactacccgtcccgtcagatctngatgctgacgncccagccctagaaccagctctagacgacagggcgnnacacacgttgccatccacaaccngnaaccntgcggccnngtcngactnnnnaatgagcaccgtacctgtnncgccgccnnnnagnnngcgnggnngaaanctgactgtgacatnngacgngagnnnnnggaacataacncccatggctngcgtccgattctttagggcngngctgcgtncggtcgtacannnaannncnnnnannngngnnacannnnnnnnnnnncaggcaccgccgagtaccgccacggaactgaatcatccgccgatctccttcggagcaccaagcgagacgttccccattacatttggggacttcaacgaaggagaaatcgaaagcttgtcttctgagctgctaactttcggagacttcctacccggagaagtggatgacttgacagatagcgactggtccacgtgctcagacacggacgacgagttactagacagggcaggtgggtatatattctcgtcggacaccggtccaggtcatttacaacagaagtcagtacgccagtcagtgctgccggtgaacaccctggaggaagtccacgaggagaagtgttacccacctaagttggatgaagtaaaggagcaactattacttaagaaactccaggagagtgcatccatggccaacagaagcaggtaccagtcgcgtaaagtagaaaacatgaaagcaacaatcatccagagactaaagagaggctgtagactatacttaatgtcagagacctcaaaagtccctacttaccggaccacatatccggcgcctgtgtactcgcctccgatcaacgtccgactgtccaaccccgagtccgcagtggcngcatgcaatgagttcctagctagaaactatccaactgtctcatcataccaaattaccgacgagtatgatgcatatttagacatggtggacgggtcggagagttgcctggatcgagcgacattcaatccgtcaaaacttaggagctacccgaaacagcacgcttaccatgcgccctccatcagaagcgctgtnnnnnnnnnnnnnnnnnnnnnnnnnnnnnnnnnnnnnnnnnnnnnnnnnnnnnnnnnnnnnnnnnnnnnnnnnnnnnnnnnnnnnnnnnnnnnnnnnnnnnnnnnnnnnnnnnnnnnnnnnnnnnnnnnnnnnnnnnnnnnnnnnnnnnnnnnnnnnnnnnnnnnnnnnnnnnnnnnnnnnnnnnnnnnnnnnnnnnnnnnnnnnnnnnnnnnnnnnnnnnnnnnnnnnnnnnnnnnnnnnnnnnnnnnnnnnnnnnnnnnnnnnntgccnctgcaggaagtaccaatggataggttcacagtagatatgaaaagggacgtgaaggtgactcctggtacaaagcacacagaggaaagacctaaggtgcaggttatacaggcggctgaacccttggcgacagcatacctatgtgggattcacagagagctggttaggaggctgaacgccgtcctcctacccaatgtacatacactatttgatatgtctgccgaggatttcgatgccatcatagccgcacactttaagccaggagacactgttttagaaacggacatagcctcctttgacaagagccaagatgattcacttgcgcttactgccttgatgttgttagaagatttaggggtggatcactccctgttggacttgatagaggccgcttttggagagatttccagctgtcannnnnnnnnnnnnnnnnnnnnnnnnnnnggcgccntgatgaaatctggtatgttnctaactctgttcgtcaacacattgcnnaacatcaccancgctagccncnngctngaagatcgtctgannaaannnncgnnnnnggncttcatcggcgacgacaacataatacatggagtcgtntccgatgaattgatggcagccagannngccacntggatgaacatnnaagngnagatcatagatgcagttgtatncntgnnagnnccntacntttgtgganggtttatactgcacgatactgtgacgggaacagcctgcagagtggcagacccgttaaaaaggctattcaaactgggcaaaccgctagcggcaggtgacgaacaagatgaagatagaagacgagcgctggctgacgaagtgatcagatggcaacgaacagggctaattgatgagttggagaaagcggtatactctaggtatgaagtgcagggtatatcagttgtggtaatgtccatggccacctttgcaagctccagatctaacttcgagaagctcagaggaccccagttaataaactgacaatgcgcgcggtaccccaacagaagccacgcaggaatcggaagaataagaagcaaaagcaaaagcgacaggcgccacaaaacaacacaaaccaaaagaagcagccgcctaaaaagaaaccagctcaaaagaaaaagaagccgggccgcagagagaggatgtgcatgaaaatcgaaaatgattgtatttttgaagtcaagtatgaaggtaaggtaacaggttacgcgtgcttggtgggggacaaagtaatgaaaccagcnnnnnnnnnnnnnnnnnnnnnnnnnnnnnnnnnnnnnnnnnnnnnnnnnnnnnnnnnnnnnnnnnnnnnnnnnnnnnnnnnnnnnnnnnnnnnnnnnnnnnnnnnnnnnnnnnnnnnnnnnnnnnnnnnnnnnnnnnnnnnnnnnnnnnnnnnnnnnnnnnnnnnnnnnnnnnnnnnnnnnnnnnnnnnnnnnnnnnnnnnnnnnnnnnnnnnnnnnnnnnnnnnnnnnnnnnnnnnnnnnnnnnnnnnnnnacgcgtggtggccatagtcttaggaggagctaatgaaggagcccgtacagccctctcggtggtgacctggaataaagacattgtcactaaaatcacccccgagggggccgaagagtggagtcttgccatcccagtcatgtgcctgctggcaaataccacgttcccctgctcccagcccccttgcactccctgctgctatgaaagggaaccagaggaaaccctacgcatgcttgaggacaacgtcatgagacctgggtactatcagctgctacaagcatnnnnnnnnnnnnnnngccacgattggaccaagctgcgttacatggacaaccacacgccagcggacgcagagagggcggggctatttgtaagaacatcagcaccgtgcacgattactggaacaatgggacacttcatcctgacccgatgtccgaaaggggaaactctgacggtgggattcactgacagtaggaagatcagtcactcatgtacgcacccatttcaccacgaccctcctgtgataggccgggagaaattccattcccgaccgcagcacnnnnnngagctgccttgcagcacgtacgtgcagagcaccgccgcaactaccgaggagatagaggtacacatgcccccagacacccctgatcgcacattgatgtcacaacagtccggcaacgtaaagatcacagttaatggccagacggtgcggtacaagtgtaattgcggtggctcaaatgaaggactaacaactacagacaaagtgattaataactgcaaagttgatcaatgtcatgccgcggtcaccaatcacaaaaagtggcagtacaactcccctctggtcccgcgtaatgctgaacttggggaccgaaaaggaaaaatccacatcccgtttccgctggcaaatgtaacatgcagggtgcctaaagcaaggaaccccaccgtgacgtacgggaaaaaccaagtcatcatgctactgtatcccgaccacccaacactcctgtcctaccggaacatgggagaagaaccaaactaccaagaagagtgggtgacgcataagaaggaagtcgtgctaaccgtgccgactgaagggctcgaggtcacgtggggtaacaacgagccgtataagtattggccgcagttatctacaaacggtacagcccatggccacccgcatgagataattctgtattattatgagctgtaccctactatgactgtagtagttgtgtcagtggcctcgttcgtactcctgtcgatggtgggtgtggcagtggggatgtgcatgtgtgcacgacgcagatgcannnnnnnnnnnnnnnnnnnnnnnnnnnnnnnnnnnnnnnnnnnnnnnnnnnnnnnnnnnnnnnnnnnnnnnnnnnnnnnnnnnnnnnnnnnnnnnnnnnnnnnnnnnnnnnnnnnnnnnnnnnnnnnnnnnnnnnnnnnnnnnnnnnnnnnnnnnnnnnnnnnnnnnnnnnnnnnnnnnnnnnnnnnnnnnnnnnnnnnnnnnnnnnnnnnnnnnnnnnnnnnnnnnnnnnnnnnnnnnnnnnnnnnnnnnnnnnnnagcgcgtacgaacacgtaacagtgatcccgaacacggtgggagtaccgtataagactctagtcaatagaccgggctacagtcccatggtattggagatggaactactgtcagtcactttggagccaacgctatcgcttgattacatcacgtgcgagtacaaaaccgttatcccgtctccgtacgtgaaatgctgcggtacagcggagtgcaaggacaaaaacctacctgactacagctgtaaggtcttcaccggcgtctacccatttatgtggggcggagcctnnnnnnnnnnnnnnnnnnccaatgtcttcagcctggacacctttcgacaacaaaattgtggtgtacaaaggtgacgtctataacatggactacccgccctttggcgcaggaagaccaggacaatttggcgatatccaaagtcgcacacctgagagtaaagacgtctatgctaatacacaactggtactgcagagaccggctgcgggtacggtacatgtgccatactctcaggcaccatctggctttaagtattggctaaaagaacgaggggcgtcgctgcagcacacagcaccatttggctgccaaatagcaacaaacccggtaagagcggtgaattgcgccgtagggaacatgcccatctccatcgacataccgga

>MK752952

cgtctgcccgatgcgcagcgcagaagatcccgagagactcgccaattatgcgagaaagttagcatctgctgcaggaaaagtcctggacagaaacatctctggaaagatcggggacttacaagcagtaatggccgtgccagacacggagacgccaacattctgcttgcacacagatgtctcatgtagacagagagcagacgtcgcgatataccaagacgtctatgctgtacacgcacccacgtcgctataccaccaggcgattaaaggggtccgagtggcgtactgggtagggttcgacacaaccccgttcatgtacaatgccatggcgggtgcctacccctcatattcgacaaactgggcagacgagcaggtactgaaggctaagaacataggattatgttcaacagacctgacggaaggtagacgaggtaaattgtctattatgagagggaagaagctaaaaccgtgtgaccgtgtgctgttctcagtagggtcaacgctctacccggaaagccgcaagctacttaagagctggcacttaccatcggtgttccatctaaagggcaaactcagcttcacatgccgctgtgatacagtggtttcgtgcgagggctacgtcgttaagagaataacgatgagcccaggcctttatggaaaaaccacagggtatgcggtaacccaccacgcagacggattcttgatgtgcaagactaccgacacggttgacggcgaaagagtgtcattctcggtgtgcacatacgtgccggcgaccatttgtgatcaaatgaccggcatccttgctacagaagtcacgccggaggatgcacagaagctgttggtggggctgaaccagagaatagtggttaacggcagaacgcaacggaatacgaacaccatgaaaaactatctgcttcccgtggtcgcccaagccttcagtaagtgggcaaaggagtgccggaaagacatggaagatgaaaaactcctgggggtcagagaaagaacactgacctgctgctgtctatgggcattcaagaagcagaaaacacacacggtctacaagaggcctgatacccagtcaattcagaaggttcaggccgagtttgacagctttgtggtaccgagcctgtggtcgtccgggatgtcaatccccttgaggactagaatcaaatggttgttaagcaaggtgccaaaaaccgacctgatcccatatagcggggacgcccaagaagcccgggacgcagaaaaagaagcagaggaagaacgagaagcannnnnnnnnnnnnnnnnnnnnnnnnnnnnnnnnnnnnnnnnnnnnnnnnnnnnnnnnnnnnnnnnnnnnnnnnagcttgaggacagagcgggtgcaggaataatagagactcctagaggagctatcaaagttactgcccaaccaacagaccacgtcgtgggagagtacctggtactttccccgcagaccgtactacggagccagaagctcagcctaattcacgctttggcggagcaagtgaagacgtgcnnnnnnnnnnnnnnnnnnnnnnnnnnnnnnnnnnnnnnnnnnnnnnnnnnnnnnnnnnnnnnnnnnnnnnnnnnnnnnnnnnnnnnnnnnnnnnnnnnnnnnnnnnnnnnnnnnnnnnnnnnnnnnnnnnnnnnnnnnnnnnnnnnnnnnnnnnnnnnnnnnnnnnnnnnnnnnnnnnnnnnnnnnnnnnnnnnnnnnnnnnnnnnnnnnnnnnnnnnnnnnnnnnnnnnnnnnnnnnnnnnnnnnnnnnnnnnnnnnnnnnnnnnnnnnnnnnnnnnnnnnnnnnnnnnnngactggtactggtgggcgacttgactaatccgccctaccacgaattcgcatatgaagggctaaaaatccgccctgcctgcccatacaaaattgcagtcataggagtcttcggagtaccaggatctggcaagtcagctattatcaagaacctagttaccaggcaagacctggtgactagcggaaagaaagaaaactgccaagaaatcaccaccgacgtgatgagacagagaggtctagagatatctgcacgtacggttgactcgctgctcttgaatggatgcaacaggccagtcgacgtgttgtacgtagacgaggcgtttgcgtgccactctggaacgttacntnntttnntcnccttngttanancnnnnnnnnnagttgtacnntgtggtganncnnagcnnnnnggcntnnncaatatgatnnagatgnaagtcaactataatnncaacatnnnnncnnnannntaccacnnnngtannnccaggcggtgtacantgnnngnnnnnnncnnnnnnncnncgttgcatnnnnnnngcnaannnnncactacgaatgagtacaacaagccgattgtagtggacactacaggctcaacaaaacctgaccctggagatctcgtgttaacgtgcttcagagggtgggttaaacaactgcaaattgactatcgtggatacgaggtcatgacagcagccgcatcccaagggttaaccagaaaaggagtttatgcagttaggcaaaaagttaatgaaaacccgctctatgcatcaacgtcagagcacgtcaacgnnnnnnnnnnnnnnnnnnnnnnnnnnnnnnnnnnnnnnnnnnnnnnnnnnnnnnnnnnnnnnnnnnnnnnnnnnnnnnnnnnnnnnnnnnnnnnnnnnnnnnnnnnnnnnnnnnnnnnnnnnnnnnnnnnnnnnnnnnnnnnnnnnnnnnnnnnnnnnnnnnnnnnnnnnnnnnnnnnnnnnnnnnnnnnnnnnnnnnnnnnnnnnnnnnnnnnnnnnnnnnnnnnnnnnnnnnnnnnnnnnnnnnnnnntgataggcagtggtcccagataattcaagccttcaaagaagataaagcatactcacctgaagtagccctgaatgaaatatgcacgcgcatgtatggggtggatttagacagtgggctattttctaaaccattggtgtctgtgtattacgcggataatcactgggataataggcctggagggaagatgttcggattcaaccccgaggcagcatccattctagaaagaaagtatccattcacaaaagggaagtggaacatcaacaagnnnnnnnnnnnnnnnnnnnnnnnnnnnnnnnnnnnnnnnnnnnnnnnnnnnnnnnnnnnnnnnnnnnnnnnnnnnnnnnnnnnnnnnnnnnnnnnnnnnnnnnnnnnnnnnnnnnnnnnnnnnnnnnnnnnnnnnnnnnnnnnnnnnnnnnnnnnnnnnnnnnnnnnnnnnnnnnnnnnnnnnnnnnnnnnnnnnnnnnnnnnnnnnnnnnnnnnnnnnnnnnnnnnnnnnnnnnnnnnnnnnnnnnnnnnnnnnnnnnnnnnnnnnnnnnnnnnnnnnnnnnnnnnnnnnnnnnnnnnnnnnnnnnnnnnnnnnnnnnnnnnnnnnnnnnnnnnnnnnnnnnnnnnnnnnnnnnnnnnnnnnnnnnnnnnnnnnnnnnnnnnnnnnnnnnnnnnnnnnnnnnnnnnnnnnnnnnnnnnnnnnnnnnnnnnnnnnnnnnnnnntagaaccagtgaacgagtcatctgcgtgttgggacgcaagttcagatcgtctagagcgttgaaaccaccatgtgtcaccagcaacactgagatgtttttcctattcagcaactttgacaatggcagaaggaatttcacaactcatgtcatgaataatcaactgaatgcagcctttgtaggacaggccacccgagcaggatgtgcaccgtcgtaccgggtaaaacgcatggacatcgcgaagaacgatnnagngtgcnnnnnnnnnnnnnnnnnnnnnnnnnnnnnnnnnnnnnnnnnnnnnnnnnnnnnnnnnnnnnnnnnnnnnnnnnnnnnnnnnnnnnnnnnnnnnnnnnnnnnnnnnnnnnnnnnnnnnnnnnnnnnnnnnnnnnnnnnnnnnnnnnnnnnnnnnnnnnnnnnnnnnnnnnnnnnnnnnnnnnnnnnnnnnnnnnnnnnnnnnnnnnnnnnnnnnnnnnnnnnnnnnnnnnnnnnnnnnnnnnnnnnnnnnnnnnnnnnnnnnnnnnnnnnnnnnnnnnnnnnnnnnnnnnnnnnnnnnnnnnnnnnnnnnnnnnnnnnnnnnnnnnnnnnnnnnnnnnnnnnnnnnnnnnnnnnnnnnnnnnnnnnnnnnnnnnnnnnnnnnnnnnnnnnnnnnnnnnnnnnnnnnnnnnnnnnnnnnnnnnnnnnnnnnnnnnnnnnnnnnnnnnnnnnnnnnnnnnnnnnnnnnnnnnnnnnnnnnnnnnnnnnnnnnnnnnnnnnnnnnnnnnnnnnnnnnnnnnnnnnnnnnnnnnnnnnnnnnnnnnnnnnnnnnnnnnnnnnnnnnnnnnnnnnnnnggcagtggacatggcagagatatatactatgtggccaaagcaaacagaggccaacgagcaagtctgcctatatgccctngggnaaagtattgaatcgatcaggcagaaatgcccggtggatgatgcagatgcatcgtctcctcnnaaaactgtcccgtgcctttgccgttacgccatgantccggaacgcgtcacccgacttcgcatgaaccacgtnncaagtataattgtgtgttcctcgtttncccttccaaagtacaaaatagaaggagtgcnaaaagtcaaatgctctaaggtaatgctatttgaccacaacgtgccatcacgcgtaagtccaagggaatatagatcttccccggaggctgcacaggaagcgagtacaaccacgtcactgacncatagccaattcgacctaagcgttgacggcgagatactacccgtcccgtcagatctngatgctgacgccccagccctagaaccagctctagacgacagggcgnnacacacgttgccatccacaaccggaaaccttgcggccgtgtctgactgggtaatgagcaccgtacctgtcgcgccgcccagaagaaggcgagggagaaacctgactgtgacatgcgacgagannnnnnggaacataacacccatggctagcgtccgattctttagggcagagctgcgtccggtcgtacaagaaacagcggagacgcgtgacacagcaatgtctcttcaggcaccgccgagtaccgccacggaactgaatcatccgccgatctccttcggagcaccaagcgagacgttccccattacatttggggacttcaacgaaggagaaatcgaaagcttgtcttctgagctgctaactttcggagacttcctacccggagaagtggatgacttgacagatagcgactggtccacgtgctcagacacggacgacgagttactagacagggcaggtgggtatatattctcgtcggacaccggtccaggtcatttacaacagaagtcagtacgccagtcagtgctgccggtgaacaccctggaggaagtccacgaggagaagtgttacccacctaagttggatgaagtaaaggagcaactattacttaagaaactccaggagagtgcatccatggccaacagaagcaggtaccagtcgcgcaaagtagaaaacatgaaagcaacaatcatccagagactaaagagaggctgtagactatacttaatgtcagagacctcaaaagtccctacttaccggaccacatatccggcgcctgtgtactcgcctccgatcaacgtccgactgtccaaccccgagtccgcagtggcagcatgcaatgagttcctagctagaaactatccaactgtctcatcataccaaattaccgacgagtatgatgcatatttagacatggtggacgggtcggagagttgcctggatcgagcgacattcaatccgtcaaaacttaggagctacccgaaacagcacgcttaccatgcgccctccatcagaagcgctgtnnnnnnnnnnnnnnnnnnnnnnnnnnnnnnnnnnnnnnnnnnnnnnnnnnnnnnnnnnnnnnnnnnnnnnnnnnnnnnnnnnnnnnnnnnnnnnnnnnnnnnnnnnnnnnnnnnnnnnnnnnnnnnnnnnnnnnnnnnnnnnnnnnnnnnnnnnnnnnnnnnnnnnnnnnnnnnnnnnnnnnnnnnnnnnnnnnnnnnnnnnnnnnnnnnnnnnnnnnnnnnnnnnnnnnnnnnnnnnnnnnnnnnnnnnnnnnnnnnnnnnnnnnnnnnnctgcaggaagtaccaatggataggttcacagtagatatgaaaagggacgtgaaggtgactcctggtacaaagcacacagaggaaagacctaaggtgcaggttatacaggcggctgaacccttggcgacagcatacctatgtgggattcacagagagctggttaggaggctgaacgccgtcctcctacccaatgtacatacactatttgatatgtctgccgaggatttcgatgccatcatagccgcacactttaagccaggagacactgttttagaaacggacatagcctcctttgacaagagccaagatgattcacttgcgcttactgccttgatgttgttagaagatttaggggtggatcactccctgttggacttgatagaggccgcttttggagagatttccagctgtcannnnnnnnnnnnnnnnnnnnnnnnnnnnnnnnnnnnnnnnnnnnnnnnnnnnnnnnnnnnnnnnnnnnnnnnnnnnnnnnnnnnnnnnnnnnnnnnnnnnnnnnnnnnnnnnnnnnnnnnnnnnnnnnnnnnnnnnnnnnnnnnnnnnnnnnnnnnnnnnnnnnnnnnnnnnnnnnnnnnnnnnnnnnnnnnnnnnnnnnnnnnnnnnnnnnnnnnnnnnnnnnnnnnnnnnnnnnnnnnnnnnnnnnnnnnnnnnnnnnnnnnnnnnnnnnnnnnnnnnnttatactgcacgatactgtgacgggaacagcctgcagagtggcagacccgctaaaaaggctattcaaactgggcaaaccgctagcggcaggtgacgaacaagatgaagatagaagacgagcgctggctgacgaagtgatcagatggcaacgaacagggctaattgatgagttggagaaagcggtatactctaggtatgaagtgcagggtatatcagttgtggtaatgtccatggccacctttgcaagctccagatctaacttcgagaagctcagaggaccccagttaataaactgacaatgcgcgcggtaccccaacagaagccacgcaggaatcggaagaataagaagcaaaagcaaaagcgacaggcgccacaaaacaacacaaaccaaaagaagcagccgcctaaaaagaaaccagctcaaaagaaaaagaagccgggccgcagagagaggatgtgcatgaaaatcgaaaatgattgtatttttgaagtcaagtatgaaggtaaggtaacaggttacgcgtgcttggtgggggacaaagtaatgaaaccagcnnnnnnnnnnnnnnnnnnnnnnnnnnnnnnnnnnnnnnnnnnnnnnnnnnnnnnnnnnnnnnnnnnnnnnnnnnnnnnnnnnnnnnnnnnnnnnnnnnnnnnnnnnnnnnnnnnnnnnnnnnnnnnnnnnnnnnnnnnnnnnnnnnnnnnnnnnnnnnnnnnnnnnnnnnnnnnnnnnnnnnnnnnnnnnnnnnnnnnnnnnnnnnnnnnnnnnnnnnnnnnnnnnnnnnnnnnnnnnnnnnnnnnnnnnnnacgcgtggtggccatagtcttaggaggagctaatgaaggagcccgtacagccctctcggtggtgacctggaataaagacattgtcactaaaatcacccccgagggggccgaagagtggagtcttgccatcccagtcatgtgcctgctggcaaataccacgttcccctgctcccagcccccttgcactccctgctgctatgaaagggaaccagaggaaaccctacgcatgcttgaggacaacgtcatgagacctgggtactatcagctgctacaagcatnnnnnnnnnnnnnnngccacgattggaccaagctgcgttacatggacaaccacacgccagcggacgcagagagggcggggctatttgtaagaacatcagcaccgtgcacgattactggaacaatgggacacttcatcctgacccgatgtccgaaaggggaaactctgacggtgggattcactgacagtaggaagatcagtcactcatgtacgcacccatttcaccacgaccctcctgtgataggccgggagaaattccattcccgaccgcagcacnnnnnngagctgccttgcagcacgtacgtgcagagcaccgccgcaactaccgaggagatagaggtacacatgcccccagacacccctgatcgcacattgatgtcacaacagtccggcaacgtaaagatcacagttaatggccagacggtgcggtacaagtgtaattgcggtggctcaaatgaaggactaataactacagacaaagtgattaataactgcaaggttgatcaatgtcatgccgcggtcaccaatcacaaaaagtggcagtacaactcccctctggtcccgcgtaatgctgaacttggggaccgaaaaggaaaaatccacatcccgtttccgctggcaaatgtaacatgcagggtgcctaaagcaaggaaccccaccgtgacgtacgggaaaaaccaagtcatcatgctactgtatcccgaccacccaacactcctgtcctaccggaacatgggagaagaaccaaactaccaagaagagtgggtgacgcataagaaggaagtcgtgctaaccgtgccgactgaagggctcgaggtcacgtggggtaacaacgagccgtataagtattggccgcagttatctacaaacggtacagcccatggccacccgcatgagataattctgtattattatgagctgtaccctactatgactgtagtagttgtgtcagtggcctcgttcgtactcctgtcgatggtgggtgtggcagtggggatgtgcatgtgtgcacgacgcagatgcannnnnnnnnnnnnnnnnnnnnnnnnnnnnnnnnnnnnnnnnnnnnnnnnnnnnnnnnnnnnnnnnnnnnnnnnnnnnnnnnnnnnnnnnnnnnnnnnnnnnnnnnnnnnnnnnnnnnnnnnnnnnnnnnnnnnnnnnnnnnnnnnnnnnnnnnnnnnnnnnnnnnnnnnnnnnnnnnnnnnnnnnnnnnnnnnnnnnnnnnnnnnnnnnnnnnnnnnnnnnnnnnnnnnnnnnnnnnnnnnnnnnnnnnnnnnnnnnagcgcgtacgaacacgtaacagtgatcccgaacacggtgggagtaccgtataagactctagtcaatagaccgggctacagtcccatggtattggagatggaactactgtcagtcactttggagccaacgctatcgcttgattacatcacgtgcgagtacaaaaccgttatcccgtctccgtacgtgaaatgctgcggtacagcagagtgcaaggacaaaaacctacctgactacagctgtaaggtcttcaccggcgtctacccatttatgtggggcggagcctnnnnnnnnnnnnnnnnnnccaatgtcttcagcctggacacctttcgacaacaaaattgtggtgtacaaaggtgacgtctataacatggactacccgccctttggcgcaggaagaccaggacaatttggcgatatccaaagtcgcacacctgagagtaaagacgtctatgctaatacacaactggtactgcagagaccggctgcgggtacggtacatgtgccatactctcaggcaccatctggctttaagtattggctaaaagaacgaggggcgtcgctgcagcacacagcaccatttggctgccaaatagcaacaaacccggtaagagcggtgaattgcgccgtagggaacatgcccatctccatcgacataccgga

>MK752953

cgtctgcccgatgcgcagcgcagaagatcccgagagactcgccaattatgcgagaaagctagcatctgctgcaggaaaagtcctggacagaaacatctctggaaagatcggggacttacaagcagtaatggccgtgccagacacggagacgccaacattctgcttgcacacagatgtctcatgtagacagagagcagacgtcgcgatataccaagacgtctatgctgtacacgcacccacgtcgctataccaccaggcgattaaaggggtccgagtggcgtactgggtagggttcgacacaaccccgttcatgtacaatgccatggcgggtgcctacccctcatattcgacaaactgggcagacgagcaggtactgaaggctaagaacataggattatgttcaacagacctgacggaaggtagacgaggtaaattgtctattatgagagggaagaagctaaaaccgtgtgaccgtgtgctgttctcagtagggtcaacgctctacccggaaagccgcaagctacttaagagctggcacttaccatcggtgttccatctaaagggcaaactcagcttcacatgccgctgtgatacagtggtttcgtgcgagggctacgtcgttaagagaataacgatgagcccaggcctttatggaaaaaccacagggtatgcggtaacccaccacgcagacggattcttgatgtgcaagactaccgacacggttgacggcgaaagagtgtcattctcggtgtgcacatacgtgccggcgaccatttgtgatcaaatgaccggcatccttgctacagaagtcacgccggaggatgcacagaagctgttggtggggctgaaccagagaatagtggttaacggcagaacgcaacggaatacgaacaccatgaaaaactatctgcttcccgtggtcgcccaagccttcagtaagtgggcaaaggagtgccggaaagacatggaagatgaaaaactcctgggggtcagagaaagaacactgacctgctgctgtctatgggcattcaagaagcagaaaacacacacggtctacaagaggcctgatacccagtcaattcagaaggttcaggccgagtttgacagctttgtggtaccgagcctgtggtcgtccgggttgtcaatccccttgaggactagaatcaaatggttgttaagcaaggtgccaaaaaccgacctgatcccatatagcggggacgcccaagaagcccgggacgcagaaaaagaagcagaggaagaacgagaagcagaactgactcgtgaagccctaccacctctacaggcagcacaggaagatgttcaggtcgaaatcgacgtggaacagcttgaggacagagcgggtgcaggaataatagagactcctagaggagctatcaaagttactgcccaaccaacagaccacgtcgtgggagagtacctggtactttccccgcagaccgtactacggagccagaagctcagcctaattcacgctttggcggagcaagtgaagacgtgnnnncacagcggacgagcagggaggtacgcggtcgaagcgtacgacggccgagtcctagtgccctcaggctatgcaatctcgcctgaagacttccagagtctaagcgaaagcgcaacgatggtgtataacgaaagagagttcgtaaacngnaagctacaccacattgcgatgcacggaccagccctgaacaccgacgaagagtcgtatgagctggtgagggcagagaggacagaacacgagtacgtctacgatgtggaccnnagaagatgctgtaagaaggaagaagctgcaggactggtactggtgggcgacttgactaatccgccctaccacgaattcgcatatgaagggctaaaaatccgccctgcctgcccatacaaaattgcagtcataggagtcttcggagtaccaggatctggcaagtcagctattatcaagaacctagttaccaggcaagacctggtgactagcggaaagaaagaaaactgccaagaaatcaccaccgacgtgatgagacagagaggtctagagatatctgcacgtacggttgactcgctgctcttgaatggatgcaacaggccagtcgacgtgttgtacgtagacgaggcgtttgngtgcnnnnnnnnnnnnnnnnnnnnnnnnnnnnnnnnnnnnnnnnnnnnnnnnnnnnnnnnnnnnnnnnnnnnnnnnnnnnnnnnnnnnnnnnnnnnnnnnnnnnnnnnnnnnnnnnnnnnnnnnnnnnnnnnnnnnnnnnnnnnnnnnnnnnnnnnnnnnnnnnnnnnnnnnnnnnnnnnnnnnnnnnnnnnnnnnnnnnnnnnnnnnnnnnnnnnnnnnnnnnnnnnnnnnnnnnagtacaacaagccgattgtagtggacactacaggctcaacaaaacctgacgctggagatctcgtgttaacgtgcttcagagggtgggttaaacaactgcaaattgactatcgtggatacgaggtcatgacagcagccgcatcccaagggttaaccagaaaaggagtttatgcagttaggcaaaaagttaatgaaaacccgctctatgcatcaacgtcagagcacgtcaacgnnnnnnnnnnnnnnnnnnnnnnnnnnnnnnnnnnnnnnnnnnnnnnnnnnnnnnnnnnnnnnnnnnnnnnnnnnnnnnnnnnnnnnnnnnnnnnnnnnnnnnnnnnnnnnnnnnnnnnnnnnnnnnnnnnnnnnnnnnnnnnnnnnnnnnnnnnnnnnnnnnnnnnnnnnnnnnnnnnnnnnnnnnnnnnnnnnnnnnnnnnnnnnnnnnnnnnnnnnnnnnnnnnnnnnnnnnnnnnnnnnnnnntaaatgataggcagtggtcccagataattcaagccttcaaagaagataaagcatactcacctgaagtagccctgaatgaaatatgcacgcgcatgtatggggtggatttagacagtgggctattttctaaaccattggtgtctgtgtattacgcggataaccactgggataataggcctggagggaagatgttcggattcaaccccgaggcagcatccattctagaaagaaagtatccattcacaaaagggaagtggaacatcaacaagnnnnnnnnnnnnnnnnnnnnnnnnnnnnnnnnnnnnnnnnnnnnnnnnnnnnnnnnnnnnnnnnnnnnnnnnnnnnnnnnnnnnnnnnnnnnnnnnnnnnnnnnnnnnnnnnnnnnnnnnnnnnnnnnnnnnnnnnnnnnnnnnnnnnnnnnnnnnnnnnnnnnnnnnnnnnnnnnnnnnnnnnnnnnnnnnnnnnnnnnnnnnnnnnnnnnnnnnnnnnnnnnnnnnnnnnnnnnnnnnnnnnnnnnnnnnnnnnnnnnnnnnnnnnnnnnnnnnnnnnnnnnnnnnnnnnnnnnnnnnnnnnnnnnnnnnnnnnnnnnnnnnnnnnnnnnnnnnnnnnnnnnnnnnnnnnnnnnnnnnnnnnnnnnnnnnnnnnnnnnnnnnnnnnnnnnnnnnnnnnnnnnnnnnnnnnnnnnnnnnnnnnnnnnnnnnnnnnnnnnnnntagaaccagtgaacgagtcatctgcgtgttgggacgcaagttcagatcgtctagagcgttgaaaccaccatgtgtcaccagcaacactgagatgtttttcctattcagcaactttgacaatggcagaaggaatttcacaactcatgtcatgaataatcaactgaatgcagcctttgtaggacaggccacccgagcaggatgtgcaccgtcgtaccgggtaaaacgcatggacatcgcgaagaacgatnnagngtgcnnnnnnnnnnnnnnnnnnnnnnnnnnnnnnnnnnnnnnnnnnnnnnnnnnnnnnnnnnnnnnnnnnnnnnnnnnnnnnnnnnnnnnnnnnnnnnnnnnnnnnnnnnnnnnnnnnnnnnnnnnnnnnnnnnnnnnnnnnnnnnnnnnnnnnnnnnnnnnnnnnnnnnnnnnnnnnnnnnnnnnnnnnnnnnnnnnnnnnnnnnnnnnnnnnnnnnnnnnnnnnnnnnnnnnnnnnnnnnnnnnnnnnnnnnnnnnnnnnnnnnnnnnnnnnnnnnnnnnnnnnnnnnnnnnnnnnnnnnnnnnnnnnnnnnnnnnnnnnnnnnnnnnnnnnnnnnnnnnnnnnnnnnnnnnnnnnnnnnnnnnnnnnnnnnnnnnnnnnnnnnnnnnnnnnnnnnnnnnnnnnnnnnnnnnnnnnnnnnnnnnnnnnnnnnnnnnnnnnnnnnnnnnnnnnnnnnnnnnnnnnnnnnnnnnnnnnnnnnnnnnnnnnnnnnnnnnnnnnnnnnnnnnnnnnnnnnnnnnnnnnnnnnnnnnnnnnnnnnnnnnnnnnnnnnnnnnnnnnnnnnnnnnnnnnnnggcagtggacatggcagagatatatactatgtggccaaagcaaacagaggccaacgagcaagtctgcctatatgccctgggggaaagtattgaatcgatcaggcagaaatgcccggtggatgatgcagatgcatcgtctcctccaaaaactgtcccgtgcctttgccgttacgccatgactccggaacgcgtcacccgacttcgcatgaaccacgtcacaagtataattgtgtgttcctcgtttccccttccaaagtacaaaatagaaggagtgcaaaaagtcaaatgctctaaggtaatgctatttgaccacaacgtgccatcacgcgtaagtccaagggaatatagatcttccccggaggctgcacaggaagcgagtacaaccacgtcactgacgcatagccaattcgacctaagcgttgacggcgagatactacccgtcccgtcagatctggatgctgacgccccagccctagaaccagctctagacgacagggcgacacacacgttgccatccacaaccggaaaccttgcggccgtgtctgactgggtaatgagcaccgtacctgtcgcgccgcccagaagaaggcgagggagaaacctgactgtgacatgcgacgagagagaagggaacataacacccatggctagcgtccgattctttagggcagagctgcgtccggtcgtacaagaaacagcggagacgcgtgacacagcaatgtctcttcaggcaccgccgagtaccgccacggaactgaatcatccgccgatctccttcggagcaccaagcgagacgttccccattacatttggggacttcaacgaaggagaaatcgaaagcttgtcttctgagctgctaactttcggagacttcctacccggagaagtggatgacttgacagatagcgactggtccacgtgctcagacacggacgacgagttactagacagggcaggtgggtatatattctcgtcggacaccggtccaggtcatttacaacagaagtcagtacgccagtcagtgctgccggtgaacaccctggaggaagtccacgaggagaagtgttacccacctaagttggatgaagtaaaggagcaactattacttaagaaactccaggagagtgcatccatggccaacagaagcaggtaccagtcgcgcaaagtagaaaacatgaaagcaacaatcatccagagactaaagagaggctgtagactatacttaatgtcagagacctcaaaagtccctacttaccggaccacatatccggcgcctgtgtactcgcctccgatcaacgtccgactgtccaaccccgagtccgcagcggcagcatgcaatgagttcctagctagaaactatccaactgtctcatcataccaaattaccgacgagtatgatgcatatttagacatggtggacgggtcggagagttgcctggatcgagcgacattcaatccgtcaaaacttaggagctacccgaaacagcacgcttaccatgcgccctccatcagaagcgctgtnnnnnnnnnnnnnnnnnnnnnnnnnnnnnnnnnnnnnnnnnnnnnnnnnnnnnnnnnnnnnnnnnnnnnnnnnnnnnnnnnnnnnnnnnnnnnnnnnnnnnnnnnnnnnnnnnnnnnnnnnnnnnnnnnnnnnnnnnnnnnnnnnnnnnnnnnnnnnnnnnnnnnnnnnnnnnnnnnnnnnnnnnnnnnnnnnnnnnnnnnnnnnnnnnnnnnnnnnnnnnnnnnnnnnnnnnnnnnnnnnnnnnnnnnnnnnnnnnnnnnnnnnnnnccactgcaggangtaccaatggataggttcacagtagatatgaaaagggacgtgaaggtgactcctggtacaaagcacacagaggaaagacctaaggtgcaggttatacaggcggctgaacccttggcgacagcatacctatgtgggattcacagagagctggttaggaggctgaacgccgtcctcctacccaatgtacatacactatttgatatgtctgccgaggatttcgatgccatcatagccgcacactttaagccaggagacactgttttagaaacggacatagcctcctttgacaagagccaagatgattcacttgcgcttactgccttgatgttgttagaagatttaggggtggatcactccctgttggacttgatagaggccgcttttggagagatttccagctgtcannnnnnnnnnnnnnnnnnnnnnnnnnnnggcgccatgatgaaatctggtatgttcctaactctgttcgtcaacacattgctaaacatcaccatcgctagccgcgtgctggaagatcgtctgacaaaanccgcgtgngcggccttcatcggcgacgacaacataatacatggagtcgtctccgatgaattgatggcagccagatgcgccacttggatgaacatggaagtgaagatcatagatgcagttgtatccttgaaagccccttacttttgtggagggtttatactgcacgatactgtgacgggaacagcctgcagagtggcagacccgctaaaaaggctattcaaactgggcaaaccgctagcggcaggtgacgaacaagatgaagatagaagacgagcgctggctgacgaagtgatcagatggcaacgaacagggctaattgatgagttggagaaagcggtatactctaggtatgaagtgcagggtatatcagttgtggtaatgtccatggccacctttgcaagctccagatctaacttcgagaagctcagaggaccccagttaataaactgacaatgcgcgcggtaccccaacagaagccacgcaggaatcggaagaataagaagcaaaagcaaaagcgacaggcgccacaaaacaacacaaaccaaaagaagcagccgcctaaaaagaaaccagctcaaaagaaaaagaagccgggccgcagagagaggatgtgcatgaaaatcgaaaatgattgtatttttgaagtcaagtatgaaggtaaggtaacaggttacgcgtgcttggtgggggacaaagtaatgaaaccagcnnnnnnnnnnnnnnnnnnnnnnnnnnnnnnnnnnnnnnnnnnnnnnnnnnnnnnnnnnnnnnnnnnnnnnnnnnnnnnnnnnnnnnnnnnnnnnnnnnnnnnnnnnnnnnnnnnnnnnnnnnnnnnnnnnnnnnnnnnnnnnnnnnnnnnnnnnnnnnnnnnnnnnnnnnnnnnnnnnnnnnnnnnnnnnnnnnnnnnnnnnnnnnnnnnnnnnnnnnnnnnnnnnnnnnnnnnnnnnnnnnnnnnnnnnnnacgcgtggtggccatagtcttaggaggagctaatgaaggagcccgtacagccctctcggtggtgacctggaataaagacattgtcactaaaatcacccccgagggggccgaagagtggagtcttgccatcccagtcatgtgcctgctggcaaataccacgttcccctgctcccagcccccttgcactccctgctgctatgaaagggaaccagaggaaaccctacgcatgcttgaggacaacgtcatgagacctgggtactatcagctgctacaagcatnnnnnnnnnnnnnnnnccacgattggaccaagctgcgttacatggacaaccacacgccagcggacgcagagagggcggggctatttgtaagaacatcagcaccgtgcacgattactggaacaatgggacacttcatcctgacccgatgtccgaaaggggaaactctgacggtgggattcactgacagtaggaagatcagtcactcatgtacgcacccatttcaccatgaccctcctgtgataggccgggagaaattccattcccgaccgcagcacnnnnnngagctgccttgcagcacgtacgtgcagagcaccgccgcaactaccgaggagatagaggtacacatgcccccagacacccctgatcgcacattgatgtcacaacagtccggcaacgtaaagatcacagttaatggccagacggtgcggtacaagtgtaattgcggtggctcaaatgaaggactaataactacagacaaagtgattaataactgcaaagttgatcaatgtcatgccgcggtcaccaatcacaaaaagtggcagtacaactcccctctggtcccgcgtaatgctgaacttggggaccgaaaaggaaaaatccacatcccgtttccgctggcaaatgtaacatgcagggtgcctaaagcaaggaaccccaccgtgacgtacgggaaaaaccaagtcatcatgctactgtatcccgaccacccaacactcctgtcctaccggaacatgggagaagaaccaaactaccaagaagagtgggtgacacataagaaggaagtcgtgctaaccgtgccgactgaagggctcgaggtcacgtggggtaacaacgagccgtataagtattggccgcagttatctacaaacggtacagcccatggccacccgcatgagataattctgtattattatgagctgtaccctactatgactgtagtagttgtgtcagtggcctcgttcgtactcctgtcgatggtgggtgtggcagtggggatgtgcatgtgcgcacgacgcagatgcannnnnnnnnnngaactgacaccaggagctaccgtccctttcctgcttagcntaatatgctgcatcagaacagctaaagcggccacataccaagaggctgcggtatacctgtggaatgagcagcaacctttgttttggctacaagcccttattccgctggcagccctgnttgtccnnnnnnnnnnnnnnnnnnnnnnnnnnnnnnnnnnnnnnnnnnnnnnnnnnnnnnnnnnnnnnnnnnnnnnnnnnnnnnnnnnnagcgcgtacgaacacgtaacagtgatcccgaacacggtgggagtaccgtataagactctagtcaatagaccgggctacagtcccatggtattggagatggaactactgtcagtcactttggagccaacgctatcgcttgattacatcacgtgcgagtacaaaaccgttatcccgtctccgtacgtgaaatgctgcggtacagcagagtgcaaggacaaaaacctacctgactacagctgtaaggtcttcaccggcgtctacccatttatgtggggcggagcctnnnnnnnnnnnnnnnnnnccaatgtcttcagcctggacacctttcgacaacaaaattgtggtgtacaaaggtgacgtctataacatggactacccgccctttggcgcaggaagaccaggacaatttggcgatatccaaagtcgcacacctgagagtaaagacgtctatgctaatacacaactggtactgcagagaccggctgcgggtacggtacatgtgccatactctcaggcaccatctggctttaagtattggctaaaagaacgaggggcgtcgctgcagcacacagcaccatttggctgccaaatagcaacaaacccggtaagagcggtgaattgcgccgtagggaacatgcccatctccatcgacataccgga

>MK752954

cgtctgcccgatgcgcagcgcagaagatcccgagagactcgccaattatgcgagaaagctagcatctgctgcaggaaaagtcctggacagaaacatctctggaaagatcggggacttacaagcagtaatggccgtgccagacacggagacgccaacattctgcttgcacacagatgtctcatgtagacagagagcagacgtcgcgatataccaagacgtctatgctgtacacgcacccacgtcgctataccaccaggcgattaaaggggtccgagtggcgtactgggtagggttcgacacaaccccgttcatgtacaatgccatggcgggtgcctacccctcatattcgacaaactgggcagacgagcaggtactgaaggctaagaacataggattatgttcaacagacctgacggaaggtagacgaggtaaattgtctattatgagagggaagaagctaaaaccgtgtgaccgtgtgctgttctcagtagggtcaacgctctacccggaaagccgcaagctacttaagagctggcacttaccatcggtgttccatctaaagggcaaactcagcttcacatgccgctgtgatacagtggtttcgtgcgagggctacgtcgttaagagaataacgatgagcccaggcctttatggaaaaaccacagggtatgcggtaacccaccacgcagacggattcttgatgtgcaagactaccgacacggttgacggcgaaagagtgtcattctcggtgtgcacatacgtgccggcgaccatttgtgatcaaatgaccggcatccttgctacagaagtcacgccggaggatgcacagaagctgttggtggggctgaaccagagaatagtggttaacggcagaacgcaacggaatacgaacaccatgaaaaactatctgcttcccgtggtcgcccaagccttcagtaagtgggcaaaggagtgccggaaagacatggaagatgaaaaactcctgggggtcagagaaagaacactgacctgctgctgtctatgggcattcaagaagcagaaaacacacacggtctacaagaggcctgatacccagtcaattcagaaggttcaggccgagtttgacagctttgtggtaccgagcctgtggtcgtccgggttgtcaatccccttgaggactagaatcaaatggttgttaagcaaggtgccaaaaaccgacctgatcccatatagcggggacgcccaagaagcccgggacgcagaaaaagaagcagaggaagaacgagaagcagaactgactcgtgaagccctaccacctctacaggcagcacaggaagatgttcaggtcgaaatcgacgtggaacagcttgaggacagagcgggtgcaggaataatagagactcctagaggagctatcaaagttactgcccaaccaacagaccacgtcgtgggagagtacctggtactttccccgcagaccgtactacggagccagaagctcagcctaattcacgctttggcggagcaagtgaagacgtgcacnnnnnnnnnnnnnnnnnnnnnnnnnnnnnnnnnnnnnnnnnnnnnnnnnnnnnnnnnnnnnnnnnnnnnnnnnnnnnnnnnnnnnnnnnnnnnnnnnnnnnnnnnnnnnnnnnnnnnnnnnnnnnnnnnnnnnnnnnnnnnnnnnnnnnnnnnnnnnnnnnnnnnnnnnnnnnnnnnnnnnnnnnnnnnnnnnnnnnnnnnnnnnnnnnnnnnnnnnnnnnnnnnnnnnnnnnnnnnnnnnnnnnnnnnnnnnnnnnnnnnnnnnnnnnnnnnnnnnnnnnnnnnnnnnngactggtactggtgggcgacttgactaatccgccctaccacgaattcgcatatgaagggctaaaaatccgccctgcctgcccatacaaaattgcagtcataggagtcttcggagtaccaggatctggcaagtcagctattatcaagaacctagttaccaggcaagacctggtgactagcggaaagaaagaaaactgccaagaaatcaccaccgacgtgatgagacagagaggtctagagatatctgcacgtacggttgactcgctgctcttgaatggatgcaacaggccagtcgacgtgttgtacgtagacgaggcgtttgcgtgcnnnnnnnnnnnnnnnnnnnnnnnnnnnnnnnnnnnnnnnnnnnnnnnnnnnnnnnnnnnnnnnnnnnnnnnnnnnnnnnnnnnnnnnnnnnnnnnnnnnnnnnnnnnnnnnnnnnnnnnnnnnnnnnnnnnnnnnnnnnnnnnnnnnnnnnnnnnnnnnnnnnnnnnnnnnnnnnnnnnnnnnnnnnnnnnnnnnnnnnnnnnnnnnnnnnnnnnnnnnnnnnnnnnnnnnnagtacaacaagccgattgtagtggacactacaggctcaacaaaacctgacgctggagatctcgtgttaacgtgcttcagagggtgggttaaacaactgcaaattgactatcgtggatacgaggtcatgacagcagccgcatcccaagggttaaccagaaaaggagtttatgcagttaggcaaaaagttaatgaaaacccgctctatgcatcaacgtcagagcacgtcaacgnnnnnnnnnnnnnnnnnnnnnnnnnnnnnnnnnnnnnnnnnnnnnnnnnnnnnnnnnnnnnnnnnnnnnnnnnnnnnnnnnnnnnnnnnnnnnnnnnnnnnnnnnnnnnnnnnnnnnnnnnnnnnnnnnnnnnnnnnnnnnnnnnnnnnnnnnnnnnnnnnnnnnnnnnnnnnnnnnnnnnnnnnnnnnnnnnnnnnnnnnnnnnnnnnnnnnnnnnnnnnnnnnnnnnnnnnnnnnnnnnnnnnnnnnntgataggcagtggtcccagataattcaagccttcaaagaagataaagcatactcacctgaagtagccctgaatgaaatatgcacgcgcatgtatggggtggatttagacagtgggctattttctaaaccattggtgtctgtgtattacgcggataaccactgggataataggcctggagggaagatgttcggattcaaccccgaggcagcatccattctagaaagaaagtatccattcacaaaagggaagtggaacatcaacaagnnnnnnnnnnnnnnnnnnnnnnnnnnnnnnnnnnnnnnnnnnnnnnnnnnnnnnnnnnnnnnnnnnnnnnnnnnnnnnnnnnnnnnnnnnnnnnnnnnnnnnnnnnnnnnnnnnnnnnnnnnnnnnnnnnnnnnnnnnnnnnnnnnnnnnnnnnnnnnnnnnnnnnnnnnnnnnnnnnnnnnnnnnnnnnnnnnnnnnnnnnnnnnnnnnnnnnnnnnnnnnnnnnnnnnnnnnnnnnnnnnnnnnnnnnnnnnnnnnnnnnnnnnnnnnnnnnnnnnnnnnnnnnnnnnnnnnnnnnnnnnnnnnnnnnnnnnnnnnnnnnnnnnnnnnnnnnnnnnnnnnnnnnnnnnnnnnnnnnnnnnnnnnnnnnnnnnnnnnnnnnnnnnnnnnnnnnnnnnnnnnnnnnnnnnnnnnnnnnnnnnnnnnnnnnnnnnnnnnnnnnntagaaccagtgaacgagtcatctgcgtgttgggacgcaagttcagatcgtctagagcgttgaaaccaccatgtgtcaccagcaacactgagatgtttttcctattcagcaactttgacaatggcagaaggaatttcacaactcatgtcatgaataatcaactgaatgcagcctttgtaggacaggccacccgagcaggatgtgcaccgtcgtaccgggtaaaacgcatggacatcgcgaagaacgatnnagngtgcnnnnnnnnnnnnnnnnnnnnnnnnnnnnnnnnnnnnnnnnnnnnnnnnnnnnnnnnnnnnnnnnnnnnnnnnnnnnnnnnnnnnnnnnnnnnnnnnnnnnnnnnnnnnnnnnnnnnnnnnnnnnnnnnnnnnnnnnnnnnnnnnnnnnnnnnnnnnnnnnnnnnnnnnnnnnnnnnnnnnnnnnnnnnnnnnnnnnnnnnnnnnnnnnnnnnnnnnnnnnnnnnnnnnnnnnnnnnnnnnnnnnnnnnnnnnnnnnnnnnnnnnnnnnnnnnnnnnnnnnnnnnnnnnnnnnnnnnnnnnnnnnnnnnnnnnnnnnnnnnnnnnnnnnnnnnnnnnnnnnnnnnnnnnnnnnnnnnnnnnnnnnnnnnnnnnnnnnnnnnnnnnnnnnnnnnnnnnnnnnnnnnnnnnnnnnnnnnnnnnnnnnnnnnnnnnnnnnnnnnnnnnnnnnnnnnnnnnnnnnnnnnnnnnnnnnnnnnnnnnnnnnnnnnnnnnnnnnnnnnnnnnnnnnnnnnnnnnnnnnnnnnnnnnnnnnnnnnnnnnnnnnnnnnnnnnnnnnnnnnnnnnnnnnnnnnggcagtggacatggcagagatatatactatgtggccaaagcaaacagaggccaacgagcaagtctgcctatatgccctgggggaaagtattgaatcgatcaggcagaaatgcccggtggatgatgcagatgcatcgtctcctccaaaaactgtcccgtgcctttgccgttacgccatgactccggaacgcgtcacccgacttcgcatgaaccacgtnncaagtataattgtgtgttcctcgtttccccttccaaagtacaaaatagaaggagtgcaaaaagtcaaatgctctaaggtaatgctatttgaccacaacgtgccatcacgcgtaagtccaagggaatatagatcttccccggaggctgcacaggaagcgagtacaaccacgtcactgacgcatagccaattcgacctaagcgttgacggcgagatactacccgtcccgtcagatctggatgctgacgccccagccctagaaccagctctagacgacagggcgacacacacgttgccatccacaaccggaaaccttgcggccgtgtctgactgggtaatgagcaccgtacctgtcgcgccgcccagaagaaggcgagggagaaacctgactgtgacatgcgacgagagagaagggaacataacacccatggctagcgtccgattctttagggcagagctgcgtccggtcgtacaagaaacagcggagacgcgtgacacagcaatgtctcttcaggcaccgccgagtaccgccacggaactgaatcatccgccgatctccttcggagcaccaagcgagacgttccccattacatttggggacttcaacgaaggagaaatcgaaagcttgtcttctgagctgctaactttcggagacttcctacccggagaagtggatgacttgacagatagcgactggtccacgtgctcagacacggacgacgagttactagacagggcaggtgggtatatattctcgtcggacaccggtccaggtcatttacaacagaagtcagtacgccagtcagtgctgccggtgaacaccctggaggaagtccacgaggagaagtgttacccacctaagttggatgaagtaaaggagcaactattacttaagaaactccaggagagtgcatccatggccaacagaagcaggtaccagtcgcgcaaagtagaaaacatgaaagcaacaatcatccagagactaaagagaggctgtagactatacttaatgtcagagacctcaaaagtccctacttaccggaccacatatccggcgcctgtgtactcgcctccgatcaacgtccgactgtccaaccccgagtccgcagcggcggcatgcaatgagttcctagctagaaactatccaactgtctcatcataccaaattaccgacgagtatgatgcatatttagacatggtggacgggtcggagagttgcctggatcgagcgacattcaatccgtcaaaacttaggagctacccgaaacagcacgcttaccatgcgccctccatcagaagcgctgtnnnnnnnnnnnnnnnnnnnnnnnnnnnnnnnnnnnnnnnnnnnnnnnnnnnnnnnnnnnnnnnnnnnnnnnnnnnnnnnnnnnnnnnnnnnnnnnnnnnnnnnnnnnnnnnnnnnnnnnnnnnnnnnnnnnnnnnnnnnnnnnnnnnnnnnnnnnnnnnnnnnnnnnnnnnnnnnnnnnnnnnnnnnnnnnnnnnnnnnnnnnnnnnnnnnnnnnnnnnnnnnnnnnnnnnnnnnnnnnnnnnnnnnnnnnnnnnnnnnnnnnnnnnnncactgcaggaagtaccaatggataggttcacagtagatatgaaaagggacgtgaaggtgactcctggtacaaagcacacagaggaaagacctaaggtgcaggttatacaggcggctgaacccttggcgacagcatacctatgtgggattcacagagagctggttaggaggctgaacgccgtcctcctacccaatgtacatacactatttgatatgtctgccgaggatttcgatgccatcatagccgcacactttaagccaggagacactgttttagaaacggacatagcctcctttgacaagagccaagatgattcacttgcgcttactgccttgatgttgttagaagatttaggggtggatcactccctgttggacttgatagaggccgcttttggagagatttccagctgtcannnnnnnnnnnnnnnnnnnnnnnnnnnnnnnnnnnnnnnnnnnnnnnnnnnnnnnnnnnnnnnnnnnnnnnnnnnnnnnnnnnnnnnnnnnnnnnnnnnnnnnnnnnnnnnnnnnnnnnnnnnnnnnnnnnnnnnnnnnnnnnnnnnnnnnnnnnnnnnnnnnnnnnnnnnnnnnnnnnnnnnnnnnnnnnnnnnnnnnnnnnnnnnnnnnnnnnnnnnnnnnnnnnnnnnnnnnnnnnnnnnnnnnnnnnnnnnnnnnnnnnnnnnnnnnnnnnnnnnnnttatactgcacgatactgtgacgggaacagcctgcagagtggcagacccgctaaaaaggctattcaaactgggcaaaccgctagcggcaggtgacgaacaagatgaagatagaagacgagcgctggctgacgaagtgatcagatggcaacgaacagggctaattgatgagttggagaaagcggtatactctaggtatgaagtgcagggtatatcagttgtggtaatgtccatggccacctttgcaagctccagatctaacttcgagaagctcagaggaccccagttaataaactgacaatgcgcgcggtancccaacagaagccacgcaggaatcggaagaataagaagcaaaagcnaaagcgacaggcgccacaaaacaacacaaaccaaaagaagcagccgcctnaaaagaaaccagctcnaaagnnaaagaagccgggccgcnnagagnggatgtgcatgaaaatcgaaaatgattgtanttttgaagtcaagtatgaaggtaaggtaacaggttacgcgtgcttggtnggggacaaagtaatgaaaccagcnnnnnnnnnnnnnnnnnnnnnnnnnnnnnnnnnnnnnnnnnnnnnnnnnnnnnnnnnnnnnnnnnnnnnnnnnnnnnnnnnnnnnnnnnnnnnnnnnnnnnnnnnnnnnnnnnnnnnnnnnnnnnnnnnnnnnnnnnnnnnnnnnnnnnnnnnnnnnnnnnnnnnnnnnnnnnnnnnnnnnnnnnnnnnnnnnnnnnnnnnnnnnnnnnnnnnnnnnnnnnnnnnnnnnnnnnnnnnnnnnnnnnnnnnnnnacgcgtggtggccatagtcttaggaggagctaatgaaggagcccgtacagccctctcggtggtgacctggaataaagacattgtcactaaaatcacccccgagggggccgaagagtggagtcttgccatcccagtcatgtgcctgctggcaaataccacgttcccctgctcccagcccccttgcactccctgctgctatgaaagggaaccagaggaaaccctacgcatgcttgaggacaacgtcatgagacctgggtactatcagctgctacaagcatnnnnnnnnnnnnnnnnccacgattggaccaagctgcgttacatggacaaccacacgccagcggacgcagagagggcggggctatttgtaagaacatcagcaccgtgcacgattactggaacaatgggacacttcatcctgacccgatgtccgaaaggggaaactctgacggtgggattcactgacagtaggaagatcagtcactcatgtacgcacccatttcaccatgaccctcctgtgataggccgggagaaattccattcccgaccgcagcacnnnnnngagctgccttgcagcacgtacgtgcagagcaccgccgcaactaccgaggagatagaggtacacatgcccccagacacccctgatcgcacattgatgtcacaacagtccggcaacgtaaagatcacagttaatggccagacggtgcggtacaagtgtaattgcggtggctcaaatgaaggactaataactacagacaaagtgattaataactgcaaagttgatcaatgtcatgccgcggtcaccaatcacaaaaagtggcagtacaactcccctctggtcccgcgtaatgctgaacttggggaccgaaaaggaaaaatccacatcccgtttccgctggcaaatgtaacatgcagggtgcctaaagcaaggaaccccaccgtgacgtacgggaaaaaccaagtcatcatgctactgtatcccgaccacccaacactcctgtcctaccggaacatgggagaagaaccaaactaccaagaagagtgggtgacacataagaaggaagtcgtgctaaccgtgccgactgaagggctcgaggtcacgtggggtaacaacgagccgtataagtattggccgcagttatctacaaacggtacagcccatggccacccgcatgagataattctgtattattatgagctgtaccctactatgactgtagtagttgtgtcagtggcctcgttcgtactcctgtcgatggtgggtgtggcagtggggatgtgcatgtgcgcacgacgcagatgcannnnnnnnnnnnnnnnnnnnnnnnnnnnnnnnnnnnnnnnnnnnnnnnnnnnnnnnnnnnnnnnnnnnnnnnnnnnnnnnnnnnnnnnnnnnnnnnnnnnnnnnnnnnnnnnnnnnnnnnnnnnnnnnnnnnnnnnnnnnnnnnnnnnnnnnnnnnnnnnnnnnnnnnnnnnnnnnnnnnnnnnnnnnnnnnnnnnnnnnnnnnnnnnnnnnnnnnnnnnnnnnnnnnnnnnnnnnnnnnnnnnnnnnnnnnnnnnnagcgcgtacgaacacgtaacagtgatcccgaacacggtgggagtaccgtataagactctagtcaatagaccgggctacagtcccatggtattggagatggaactactgtcagtcactttggagccaacgctatcgcttgattacatcacgtgcgagtacaaaaccgttatcccgtctccgtacgtgaaatgctgcggtacagcagagtgcaaggacaaaaacctacctgactacagctgtaaggtcttcaccggcgtctacccatttatgtggggcggagcctnnnnnnnnnnnnnnnnnnccaatgtcttcagcctggacacctttcgacaacaaaattgtggtgtacaaaggtgacgtctataacatggactacccgccctttggcgcaggaagaccaggacaatttggcgatatccaaagtcgcacacctgagagtaaagacgtctatgctaatacacaactggtactgcagagaccggctgcgggtacggtacatgtgccatactctcaggcaccatctggctttaagtattggctaaaagaacgaggggcgtcgctgcagcacacagcaccatttggctgccaaatagcaacaaacccggtaagagcggtgaattgcgccgtagggaacatgcccatctccatcgacataccgga

>MK752955

cgtctgcccgatgcgcagcgcagaagatcccgagagactcgccaattatgcgagaaagctagcatctgctgcaggaaaagtcctggacagaaacatctctggaaagatcggggacttacaagcagtaatggccgtgccagacacggagacgccaacattctgcttgcacacagatgtctcatgtagacagagagcagacgtcgcgatataccaagacgtctatgctgtacacgcacccacgtcgctataccaccaggcgattaaaggggtccgagtggcgtactgggtagggttcgacacaaccccgttcatgtacaatgccatggcgggtgcctacccctcatattcgacaaactgggcagacgagcaggtactgaaggctaagaacataggattatgttcaacagacctgacggaaggtagacgaggtaaattgtctattatgagagggaagaagctaaaaccgtgtgaccgtgtgctgttctcagtagggtcaacgctctacccggaaagccgcaagctacttaagagctggcacttaccatcggtgttccatctaaagggcaaactcagcttcacatgccgctgtgatacagtggtttcgtgcgagggctacgtcgttaagagaataacgatgagcccaggcctttatggaaaaaccacagggtatgcggtaacccaccacgcagacggattcttgatgtgcaagactaccgacacggttgacggcgaaagagtgtcattctcggtgtgcacatacgtgccggcgaccatttgtgatcaaatgaccggcatccttgctacagaagtcacgccggaggatgcacagaagctgttggtggggctgaaccagagaatagtggttaacggcagaacgcaacggaatacgaacaccatgaaaaactatctgcttcccgtggtcgcccaagccttcagtaagtgggcaaaggagtgccggaaagacatggaagatgaaaaactcctgggggtcagagaaagaacactgacctgctgctgtctatgggcattcaagaagcagaaaacacacacggtctacaagaggcctgatacccagtcaattcagaaggttcaggccgagtttgacagctttgtggtaccgagcctgtggtcgtccgggttgtcaatccccttgaggactagaatcaaatggttgttaagcaagttgccaaaaaccgacctgatcccatatagcggggacgcccaagaagcccgggacgcagaaaaagaagcagaggaagaacgagaagcagaannnnnnnnnnnnnnnnnnnnnnnnnnnnnnnnnnnnnnnnnnnnnnntcaggtcgaaatcgacgtggaacagcttgaggacagagcgggtgcaggaataatagagactcctagaggagctatcaaagttactgcccaaccaacagaccacgtcgtgggagagtacctggtactttccccgcagaccgtactacggagccagaagctcagcctaattcacgctttggcggagcaagtgaagacgtgcacnnnnnnnnnnnnnnnnnnnnnnnnnnnnnnnnnnnnnnnnnnnnnnnnnnnnnnnnnnnnnnnnnnnnnnnnnnnnnnnnnnnnnnnnnnnnnnnnnnnnnnnnnnnnnnnnnnnnnnnnnnnnnnnnnnnnnnnnnnnnnnnnnnnnnnnnnnnnnnnnnnnnnnnnnnnnnnnnnnnnnnnnnnnnnnnnnnnnnnnnnnnnnnnnnnnnnnnnnnnnnnnnnnnnnnnnnnnnnnnnnnnnnnnnnnnnnnnnnnnnnnnnnnnnnnnnnnnnnnnnnnnnnnnnnngactggtactggtgggcgacttgactaatccgccctaccacgaattcgcatatgaagggctaaaaatccgccctgcctgcccatacaaaattgcagtcataggagtcttcggagtaccaggatctggcaagtcagctattatcaagaacctagttaccaggcaagacctggtgactagcggaaagaaagaaaactgccaagaaatcaccaccgacgtgatgagacagagaggtctagagatatctgcacgtacggttgactcgctgctcttgaatggatgcaacaggccagtcgacgtgttgtacgtagacgaggcgtttgcgtgccactctggaacgttacttgctttgatcgccttggttagaccaaggcagaaagttgtactctgtggtgacccgaagcagtgcggcttcttcaatatgatgcagatgaaagtcaactataatcacaacatctgcacccaagtgtaccacaaaagtatctccaggcggtgtacactgcctgtgaccgccattgtgtcatcgttgcattacgaaggcaaaatgcgcactacgaatgagtacaacaagccgattgtagtggacactacaggctcaacaaaacctgaccctggagatctcgtgttaacgtgcttcagagggtgggttaaacaactgcaaattgactatcgtggatacgaggtcatgacagcagccgcatcccaagggttaaccagaaaaggagtttatgcagttaggcaaaaagttaatgaaaacccgctctatgcatcaacgtcagagcacgtcaacgnnnnnnnnnnnnnnnnnnnnnnnnnnnnnnnnnnnnnnnnnnnnnnnnnnnnnnnnnnnnnnnnnnnnnnnnnnnnnnnnnnnnnnnnnnnnnnnnnnnnnnnnnnnnnnnnnnnnnnnnnnnnnnnnnnnnnnnnnnnnnnnnnnnnnnnnnnnnnnnnnnnnnnnnnnnnnnnnnnnnnnnnnnnnnnnnnnnnnnnnnnnnnnnnnnnnnnnnnnnnnnnnnnnnnnnnnnnnnnnnnnnnnntaaatgataggcagtggtcccagataattcaagccttcaaagaagataaagcatactcacctgaagtagccctgaatgaaatatgcacgcgcatgtatggggtggatttagacagtgggctattttctaaaccattggtgtctgtgtattacgcggataatcactgggataataggcctggagggaagatgttcggattcaaccccgaggcagcatccattctagaaagaaagtatccattcacaaaagggaagtggaacatcaacaagnnnnnnnnnnnnnnnnnnnnnnnnnnnnnnnnnnnnnnnnnnnnnnnnnnnnnnnnnnnnnnnnnnnnnnnnnnnnnnnnnnnnnnnnnnnnnnnnnnnnnnnnnnnnnnnnnnnnnnnnnnnnnnnnnnnnnnnnnnnnnnnnnnnnnnnnnnnnnnnnnnnnnnnnnnnnnnnnnnnnnnnnnnnnnnnnnnnnnnnnnnnnnnnnnnnnnnnnnnnnnnnnnnnnnnnnnnnnnnnnnnnnnnnnnnnnnnnnnnnnnnnnnnnnnnnnnnnnnnnnnnnnnnnnnnnnnnnnnnnnnnnnnnnnnnnnnnnnnnnnnnnnnnnnnnnnnnnnnnnnnnnnnnnnnnnnnnnnnnnnnnnnnnnnnnnnnnnnnnnnnnnnnnnnnnnnnnnnnnnnnnnnnnnnnnnnnnnnnnnnnnnnnnnnnnnnnnnnnnnnnnntagaaccagtgaacgagtcatctgcgtgttgggacgcaagttcagatcgtctagagcgttgaaaccaccatgtgtcaccagcaacactgagatgtttttcctattcagcaactttgacaatggcagaaggaatttcacaactcatgtcatgaataatcaactgaatgcagcctttgtaggacaggccacccgagcaggatgtgcaccgtcgtaccgggtaaaacgcatggacatcgcgaagaacgatgaagngtgcnnnnnnnnnnnnnnnnnnnnnnnnnnnnnnnnnnnnnnnnnnnnnnnnnnnnnnnnnnnnnnnnnnnnnnnnnnnnnnnnnnnnnnnnnnnnnnnnnnnnnnnnnnnnnnnnnnnnnnnnnnnnnnnnnnnnnnnnnnnnnnnnnnnnnnnnnnnnnnnnnnnnnnnnnnnnnnnnnnnnnnnnnnnnnnnnnnnnnnnnnnnnnnnnnnnnnnnnnnnnnnnnnnnnnnnnnnnnnnnnnnnnnnnnnnnnnnnnnnnnnnnnnnnnnnnnnnnnnnnnnnnnnnnnnnnnnnnnnnnnnnnnnnnnnnnnnnnnnnnnnnnnnnnnnnnnnnnnnnnnnnnnnnnnnnnnnnnnnnnnnnnnnnnnnnnnnnnnnnnnnnnnnnnnnnnnnnnnnnnnnnnnnnnnnnnnnnnnnnnnnnnnnnnnnnnnnnnnnnnnnnnnnnnnnnnnnnnnnnnnnnnnnnnnnnnnnnnnnnnnnnnnnnnnnnnnnnnnnnnnnnnnnnnnnnnnnnnnnnnnnnnnnnnnnnnnnnnnnnnnnnnnnnnnnnnnnnnnnnnnnnnnnnnnnnnnggcagtggacatggcagagatatatactatgtggccaaagcaaacagaggccaacgagcaagtctgcctatatgccctgggggaaagtattgaatcgatcaggcagaaatgcccggtggatgatgcagatgcatcgtctcctccaaaaactgtcccgtgcctttgccgttacgccatgactccggaacgcgtcacccgacttcgcatgaaccacgtcacaagtataattgtgtgttcctcgtttccccttccaaagtacaaaatagaaggagtgcnaaaagtcaaatgctctaaggtaatgctatttgaccacaacgtgccatcacgcgtaagtccaagggaatatagatcttccccggaggctgcacaggaagcgagtacaaccacgtcactgacgcatagccaattcgacctaagcgttgacggcgagatactacccgtcccgtcagatctggatgctgacgccccagccctagaaccagctctagacgacagggcgacacacacgttgccatccacaaccggaaaccttgcggccgtgtctgactgggtaatgagcaccgtacctgtcgcgccgcccagaagaaggcgagggagaaacctgactgtgacatgcgacgagagagaagggaacataacacccatggctagcgtccgattctttagggcagagctgcgtccggtcgtacaagaaacagcggagacgcgtgacacagcaatgtctcttcaggcaccgccgagtaccgccacggaactgaatcatccgccgatctccttcggagcaccaagcgagacgttccccattacatttggggacttcaacgaaggagaaatcgaaagcttgtcttctgagctgctaactttcggagacttcctacccggagaagtggatgacttgacagatagcgactggtccacgtgctcagacacggacgacgagttactagacagggcaggtgggtatatattctcgtcggacaccggtccaggtcatttacaacagaagtcagtacgccagtcagtgctgccggtgaacaccctggaggaagtccacgaggagaagtgttacccacctaagttggatgaagtaaaggagcaactattacttaagaaactccaggagagtgcatccatggccaacagaagcaggtaccagtcgcgcaaagtagaaaacatgaaagcaacaatcatccagagactaaagagaggctgtagactatacttaatgtcagagacctcaaaagtccctacttaccggaccacatatccggcgcctgtgtactcgcctccgatcaacgtccgactgtccaaccccgagtccgcagtggcagcatgcaatgagttcctagctagaaactatccaactgtctcatcataccaaattaccgacgagtatgatgcatatttagacatggtggacgggtcggagagttgcctggatcgagcgacattcaatccgtcaaaacttaggagctacccgaaacagcacgcttaccatgcgccctccatcagaagcgctgtnnnnnnnnnnnnnnnnnnnnnnnnnnnnnnnnnnnnnnnnnnnnnnnnnnnnnnnnnnnnnnnnnnnnnnnnnnnnnnnnnnnnnnnnnnnnnnnnnnnnnnnnnnnnnnnnnnnnnnnnnnnnnnnnnnnnnnnnnnnnnnnnnnnnnnnnnnnnnnnnnnnnnnnnnnnnnnnnnnnnnnnnnnnnnnnnnnnnnnnnnnnnnnnnnnnnnnnnnnnnnnnnnnnnnnnnnnnnnnnnnnnnnnnnnnnnnnnnnnnnnnnnnctgccactgcaggaagtaccaatggataggttcacagtagatatgaaaagggacgtgaaggtgactcctggtacaaagcacacagaggaaagacctaaggtgcaggttatacaggcggctgaacccttggcgacagcatacctatgtgggattcacagagagctggttaggaggctgaacgccgtcctcctacccaatgtacatacactatttgatatgtctgccgaggatttcgatgccatcatagccgcacactttaagccaggagacactgttttagaaacggacatagcctcctttgacaagagccaagatgattcacttgcgcttactgccttgatgttgttagaagatttaggggtggatcactccctgttggacttgatagaggccgcttttggagagatttccagctgtcannnnnnnnnnnnnnnnnnnnnnnnnnnnggcgccatgatgaaatctggtatgttcctaactctgttcgtcaacacattgctaaacatcaccatcgctagccgngtgctggaagatcgtctgacaaaatccgcgtgcgcggccttcatcggcgacgacaacataatacatggngtcgtctccgatgaattgatggcagccagatgcgccacttggatgaacatggaagtgaagatcatagatgcagttgtatccttgnaagncccttacntttgtgganggtttatactgcacgatactgtgacgggaacagcctgcagagtggcagacccgctaaaaaggctattcaaactgggcaaaccgctagcggcaggtgacgaacaagatgaagatagaagacgagcgctggctgacgaagtgatcagatggcaacgaacagggctaattgatgagttggagaaagcggtatactctaggtatgaagtgcagggtatatcagttgtggtaatgtccatggccacctttgcaagctccagatctaacttcgagaagctcagaggaccccagttaataaactgacaatgcgcgcggtaccccaacagaagccacgcaggaatcggaagaataagaagcaaaagcaaaagcgacaggcgccacaaaacaacacaaaccaaaagaagcagccgcctaaaaagaaaccagctcaaaagaaaaagaagccgggccgcagagagaggatgtgcatgaaaatcgaaaatgattgtatttttgaagtcaagtatgaaggtaaggtaacaggttacgcgtgcttggtgggggacaaagtaatgaaaccagcnnnnnnnnnnnnnnnnnnnnnnnnnnnnnnnnnnnnnnnnnnnnnnnnnnnnnnnnnnnnnnnnnnnnnnnnnnnnnnnnnnnnnnnnnnnnnnnnnnnnnnnnnnnnnnnnnnnnnnnnnnnnnnnnnnnnnnnnnnnnnnnnnnnnnnnnnnnnnnnnnnnnnnnnnnnnnnnnnnnnnnnnnnnnnnnnnnnnnnnnnnnnnnnnnnnnnnnnnnnnnnnnnnnnnnnnnnnnnnnnnnnnnnnnnnnnacgcgtggtggccatagtcttaggaggagctaatgaaggagcccgtacagccctctcggtggtgacctggaataaagacattgtcactaaaatcacccccgagggggccgaagagtggagtcttgccatcccagtcatgtgcctgctggcaaataccacgttcccctgctcccagcccccttgcactccctgctgctatgaaagggaaccagaggaaaccctacgcatgcttgaggacaacgtcatgagacctgggtactatcagctgctacaagcatnnnnnnnnnnnnnnngccacgattggaccaagctgcgttacatggacaaccacacgccagcggacgcagagagggcggggctatttgtaagaacatcagcaccgtgcacgattactggaacaatgggacacttcatcctgacccgatgtccgaaaggggaaactctgacggtgggattcactgacagtaggaagatcagtcactcatgtacgcacccatttcaccacgaccctcctgtgataggccgggagaaattccattcccgaccgcagcacnnnnnngagctgccttgcagcacgtacgtgcagagcaccgccgcaactaccgaggagatagaggtacacatgcccccagacacccctgatcgcacattgatgtcacaacagtccggcaacgtaaagatcacagttaatggccagacggtgcggtacaagtgtaattgcggtggctcaaatgaaggactaataactacagacaaagtgattaataactgcaaagttgatcaatgtcatgccgcggtcaccaatcacaaaaagtggcagtacaactcccctctggtcccgcgtaatgctgaacttggggaccgaaaaggaaaaatccacatcccgtttccgctggcaaatgtaacatgcagggtgcctaaagcaaggaaccccaccgtgacgtacgggaaaaaccaagtcatcatgctactgtatcccgaccacccaacactcctgtcctaccggaacatgggagaagaaccaaactaccaagaagagtgggtgacgcataagaaggaagtcgtgctaaccgtgccgactgaagggctcgaggtcacgtggggtaacaacgagccgtataagtattggccgcagttatctacaaacggtacagcccatggccacccgcatgagataattctgtattattatgagctgtaccctactatgactgtagtagttgtgtcagtggcctcgttcgtactcctgtcgatggtgggtgtggcagtggggatgtgcatgtgtgcacgacgcagatgcannnnnnnnnnnnnnnnnnnnnnnnnnnnnnnnnnnnnnnnnnnnnnnnnnnnnnnnnnnnnnnnnnnnnnnnnnnnnnnnnnnnnnnnnnnnnnnnnnnnnnnnnnnnnnnnnnnnnnnnnnnnnnnnnnnnnnnnnnnnnnnnnnnnnnnnnnnnnnnnnnnnnnnnnnnnnnnnnnnnnnnnnnnnnnnnnnnnnnnnnnnnnnnnnnnnnnnnnnnnnnnnnnnnnnnnnnnnnnnnnnnnnnnnnnnnnnnnnagcgcgtacgaacacgtaacagtgatcccgaacacggtgggagtaccgtataagactctagtcaacagaccgggctacagtcccatggtattggagatggaactactgtcagtcactttggagccaacgctatcgcttgattacatcacgtgcgagtacaaaaccgttatcccgtctccgtacgtgaaatgctgcggtacagcagagtgcaaggacaaaaacctacctgactacagctgtaaggtcttcaccggcgtctacccatttatgtggggcggagcctnnnnnnnnnnnnnnnnnnccaatgtcttcagcctggacacctttcgacaacaaaattgtggtgtacaaaggtgacgtctataacatggactacccgccctttggcgcaggaagaccaggacaatttggcgatatccaaagtcgcacacctgagagtaaagacgtctatgctaatacacaactggtactgcagagaccggctgcgggtacggtacatgtgccatactctcaggcaccatctggctttaagtattggctaaaagaacgaggggcgtcgctgcagcacacagcaccatttggctgccaaatagcaacaaacccggtaagagcggtgaattgcgccgtagggaacatgcccatctccatcgacataccgga

>MK752956

cgtctgcccgatgcgcagcgcagaagatcccgagagactcgccaattatgcgagaaagctagcatctgctgcaggaaaagtcctggacagaaacatctctggaaagatcggggacttacaagcagtaatggccgtgccagacacggagacgccaacattctgcttgcacacagatgtctcatgtagacagagagcagacgtcgcgatataccaagacgtctatgctgtacacgcacccacgtcgctataccaccaggcgattaaaggggtccgagtggcgtactgggtagggttcgacacaaccccgttcatgtacaatgccatggcgggtgcctacccctcatattcgacaaactgggcagacgagcaggtactgaaggctaagaacataggattatgttcaacagacctgacggaaggtagacgaggtaaattgtctattatgagagggaagaagctaaaaccgtgtgaccgtgtgctgttctcagtagggtcaacgctctacccggaaagccgcaagctacttaagagctggcacttaccatcggtgttccatctaaagggcaaactcagcttcacatgccgctgtgatacagtggtttcgtgcgagggctacgtcgttaagagaataacgatgagcccaggcctttatggaaaaaccacagggtatgcggtaacccaccacgcagacggattcttgatgtgcaagactaccgacacggttgacggcgaaagagtgtcattctcggtgtgcacatacgtgccggcgaccatttgtgatcaaatgaccggcatccttgctacagaagtcacgccggaggatgcacagaagctgttggtggggctgaaccagagaatagtggttaacggcagaacgcaacggaatacgaacaccatgaaaaactatctgcttcccgtggtcgcccaagccttcagtaagtgggcaaaggagtgccggaaagacatggaagatgaaaaactcctgggggtcagagaaagaacactgacctgctgctgtctatgggcattcaagaagcagaaaacacacacggtctacaagaggcctgatacccagtcaattcagaaggttcaggccgagtttgacagctttgtggtaccgagcctgtggtcgtccgggttgtcaatccccttgaggactagaatcaaatggttgttaagcaaggtgccaaaaaccgacctgatcccatatagcggggacgcccaagaagcccgggacgcagaaaaagaagcagaggaagaacgagaagcagaactgactcgtgaagccctaccacctctacaggcagcacaggaagatgttcaggtcgaaatcgacgtggaacagcttgaggacagagcgggtgcaggaataatagagactcctagaggagctatcaaagttactgcccaaccaacagaccacgtcgtgggagagtacctggtactttccccgcagaccgtactacggagccagaagctcagcctaattcacgctttggcggagcaagtgaagacgtgcacgcacagcggacgagcagggaggtacgcggtcgaagcgtacgacggccgagtcctagtgccctcaggctatgcaatctcgcctgaagacttccagagtctaagcgaaagcgcaacgatggtgtataacgaaagagagttcgtaaacagaaagctacaccacattgcgatgcacggaccagccctgaacaccgacgaagagtcgtatgagctggtgagggcagagaggacagaacacgagtacgtctacgatgtggaccagagaagatgctgtaagaaggaagaagctgcaggactggtactggtgggcgacttgactaatccgccctaccacgaattcgcatatgaagggctaaaaatccgccctgcctgcccatacaaaattgcagtcataggagtcttcggagtaccaggatctggcaagtcagctattatcaagaacctagttaccaggcaagacctggtgactagcggaaagaaagaaaactgccaagaaatcaccaccgacgtgatgagacagagaggtctagagatatctgcacgtacggttgactcgctgctcttgaatggatgcaacaggccagtcgacgtgttgtacgtagacgaggcgtttgngtgcnnnnnnnnnnnnnnnnnnnnnnnnnnnnnnnnnnnnnnnnnnnnnnnnnnnnnnnnnnnnnnnnnnnnnnnnnnnnnnnnnnnnnnnnnnnnnnnnnnnnnnnnnnnnnnnnnnnnnnnnnnnnnnnnnnnnnnnnnnnnnnnnnnnnnnnnnnnnnnnnnnnnnnnnnnnnnnnnnnnnnnnnnnnnnnnnnnnnnnnnnnnnnnnnnnnnnnnnnnnnnnnnnnnnnnnnagtacaacaagccgattgtagtggacactacaggctcaacaaaacctgacgctggagatctcgtgttaacgtgcttcagagggtgggttaaacaactgcaaattgactatcgtggatacgaggtcatgacagcagccgcatcccaagggttaaccagaaaaggagtttatgcagttaggcaaaaagttaatgaaaacccgctctatgcatcaacgtcagagcacgtcaacgnnnnnnnnnnnnnnnnnnnnnnnnnnnnnnnnnnnnnnnnnnnnnnnnnnnnnnnnnnnnnnnnnnnnnnnnnnnnnnnnnnnnnnnnnnnnnnnnnnnnnnnnnnnnnnnnnnnnnnnnnnnnnnnnnnnnnnnnnnnnnnnnnnnnnnnnnnnnnnnnnnnnnnnnnnnnnnnnnnnnnnnnnnnnnnnnnnnnnnnnnnnnnnnnnnnnnnnnnnnnnnnnnnnnnnnnnnnnnnnnnnnnnnnnaatgataggcagtggtcccagataattcaagccttcaaagaagataaagcatactcacctgaagtagccctgaatgaaatatgcacgcgcatgtatggggtggatttagacagtgggctattttctaaaccattggtgtctgtgtattacgcggataaccactgggataataggcctggagggaagatgttcggattcaaccccgaggcagcatccattctagaaagaaagtatccattcacaaaagggaagtggaacatcaacaagnnnnnnnnnnnnnnnnnnnnnnnnnnnnnnnnnnnnnnnnnnnnnnnnnnnnnnnnnnnnnnnnnnnnnnnnnnnnnnnnnnnnnnnnnnnnnnnnnnnnnnnnnnnnnnnnnnnnnnnnnnnnnnnnnnnnnnnnnnnnnnnnnnnnnnnnnnnnnnnnnnnnnnnnnnnnnnnnnnnnnnnnnnnnnnnnnnnnnnnnnnnnnnnnnnnnnnnnnnnnnnnnnnnnnnnnnnnnnnnnnnnnnnnnnnnnnnnnnnnnnnnnnnnnnnnnnnnnnnnnnnnnnnnnnnnnnnnnnnncatnaacannnacacaccnnnncgcatacaccattaccaacannnnntagaccncgcaatgaaactgcaaatgctcnggggtgactcattgagactgctcaaaccgggtggnnctcnattgatcagagcatatggttacgcagatagaaccagtgaacgagtcatctgcgtgttgggacgcaagttcagatcgtctagagcgttgaaaccaccatgtgtcaccagcaacactgagatgtttttcctattcagcaactttgacaatggcagaaggaatttcacaactcatgtcatgaataatcaactgaatgcagcctttgtaggacaggccacccgagcaggatgtgcaccgtcgtaccgggtaaaacgcatggacatcgcgaagaacgatgnagngtgcnnnnnnnnnnnnnnnnnnnnnnnnnnnnnnnnnnnnnnnnnnnnnnnnnnnnnnnnnnnnnnnnnnnnnnnnnnnnnnnnnnnnnnnnnnnnnnnnnnnnnnnnnnnnnnnnnnnnnnnnnnnnnnnnnnnnnnnnnnnnnnnnnnnnnnnnnnnnnnnnnnnnnnnnnnnnnnnnnnnnnnnnnnnnnnnnnnnnnnnnnnnnnnnnnnnnnnnnnnnnnnnnnnnnnnnnnnnnnnnnnnnnnnnnnnnnnnnnnnnnnnnnnnnnnnnnnnnnnnnnnnnnnnnnnnnnnnnnnnnnnnnnnnnnnnnnnnnnnnnnnnnnnnnnnnnnnnnnnnnnnnnnnnnnnnnnnnnnnnnnnnnnnnnnnnnnnnnnnnnnnnnnnnatnggagaagnaaatatccgaggccatacagatgcggactcaagtggagttgctggatgagcacatctccatagactgcgatattgttcgcgtgcaccctgacagcagcttggcaggcagaaaaggatacagcaccacggaaggnnnnnnnnnnnnnnnnnnnnnnnnnnnnnnnnnnnnnnnnnnggcagtggacatggcagagatatatactatgtggccaaagcaaacagaggccaacgagcaagtctgcctatatgccctgggggaaagtattgaatcgatcaggcagaaatgcccggtggatgatgcagatgcatcgtctcctccaaaaactgtcccgtgcctttgccgttacgccatgactccggaacgcgtcacccgacttcgcatgaaccacgtcacaagtataattgtgtgttcctcgtttccccttccaaagtacaaaatagaaggagtgcaaaaagtcaaatgctctaaggtaatgctatttgaccacaacgtgccatcacgcgtaagtccaagggaatatagatcttccccggaggctgcacaggaagcgagtacaaccacgtcactgacgcatagccaattcgacctaagcgttgacggcgagatactacccgtcccgtcagatctggatgctgacgccccagccctagaaccagctctagacgacagggcgacacacacgttgccatccacaaccggaaaccttgcggccgtgtctgactgggtaatgagcaccgtacctgtcgcgccgcccagaagaaggcgagggagaaacctgactgtgacatgcgacgagagagaagggaacataacacccatggctagcgtccgattctttagggcagagctgcgtccggtcgtacaagaaacagcggagacgcgtgacacagcaatgtctcttcaggcaccgccgagtaccgccacggaactgaatcatccgccgatctccttcggagcaccaagcgagacgttccccattacatttggggacttcaacgaaggagaaatcgaaagcttgtcttctgagctgctaactttcggagacttcctacccggagaagtggatgacttgacagatagcgactggtccacgtgctcagacacggacgacgagttactagacagggcaggtgggtatatattctcgtcggacaccggtccaggtcatttacaacagaagtcagtacgccagtcagtgctgccggtgaacaccctggaggaagtccacgaggagaagtgttacccacctaagttggatgaagtaaaggagcaactattacttaagaaactccaggagagtgcatccatggccaacagaagcaggtaccagtcgcgcaaagtagaaaacatgaaagcaacaatcatccagagactaaagagaggctgtagactatacttaatgtcagagacctcaaaagtccctacttaccggaccacatatccggcgcctgtgtactcgcctccgatcaacgtccgactgtccaaccccgagtccgcagcggcggcatgcaatgagttcctagctagaaactatccaactgtctcatcataccaaattaccgacgagtatgatgcatatttagacatggtggacgggtcggagagttgcctggatcgagcgacattcaatccgtcaaaacttaggagctacccgaaacagcacgcttaccatgcgccctccatcagaagcgctgtnnnnnnnnnnnnnnnnnnnnnnnnnnnnnnnnnnnnnnnnnnnnnnnnnnnnnnnnnnnnnnnnnnnnnnnnnnnnnnnnnnnnnnnnnnnnnnnnnnnnnnnnnnnnnnnnnnnnnnnnnnnnnnnnnnnnnnnnnnnnnnnnnnnnnnnnnnnnnnnnnnnnnnnnnnnnnnnnnnnnnnnnnnnnnnnnnnnnnnnnnnnnnnnnnnnnnnnnnnnnnnnnnnnnnnnnnnnnnnnnnnnnnnnnnnnnnnnnnnnnatctgctgccactgcaggaagtaccaatggataggttcacagtagatatgaaaagggacgtgaaggtgactcctggtacaaagcacacagaggaaagacctaaggtgcaggttatacaggcggctgaacccttggcgacagcatacctatgtgggattcacagagagctggttaggaggctgaacgccgtcctccnacccaatgtacatacactatttgatatgtctgccgaggatttcgatgccatcatagccgcacactttaagccaggagacactgttttagaaacggacatagcctcctttgacaagagccaagatgattcacttgcgcttactgccttgatgttgttagaagatttaggggtggatcactccctgttggacttgatagaggccgcttttggagagatttccagctgtcannnnnnnnnnnnnnnnnnnnnnnnnnnnggcgccatgatgaaatctggtatgttcctaactctgttcgtcaacacattgctaaacatcaccatcgctagccgcgtgctggaagatcgtctgacaaaatccgcgtgcgcggccttcatcggcgacgacaacataatacatggagtcgtctccgatgaattgatggcagccagatgcgccacttggatgaacatggaagtgaagatcatagatgcagttgtatccttgaaagccccttacttttgtgganggtttatactgcacgatactgtgacgggaacagcctgcagagtggcagacccgctaaaaaggctattcaaactgggcaaaccgctagcggcaggtgacgaacaagatgaagatagaagacgagcgctggctgacgaagtgatcagatggcaacgaacagggctaattgatgagttggagaaagcggtatactctaggtatgaagtgcagggtatatcagttgtggtaatgtccatggccacctttgcaagctccagatctaacttcgagaagctcagaggaccccagttaataaactgacaatgcgcgcggtaccccaacagaagccacgcaggaatcggaagaataagaagcaaaagcaaaagcgacaggcgccacaaaacaacacaaaccaaaagaagcagccgcctaaaaagaaaccagctcaaaagaaaaagaagccgggccgcagagagaggatgtgcatgaaaatcgaaaatgattgtatttttgaagtcaagtatgaaggtaaggtaacaggttacgcgtgcttggtgggggacaaagtaatgaaaccagcnnnnnnnnnnnnnnnnnnnnnnnnnnnnnnnnnnnnnnnnnnnnnnnnnnnnnnnnnnnnnnnnnnnnnnnnnnnnnnnnnnnnnnnnnnnnnnnnnnnnnnnnnnnnnnnnnnnnnnnnnnnnnnnnnnnnnnnnnnnnnnnnnnnnnnnnnnnnnnnnnnnnnnnnnnnnnnnnnnnnnnnnnnnnnnnnnnnnnnnnnnnnnnnnnnnnnnnnnnnnnnnnnnnnnnnnnnnnnnnnnnnnnnnnnnnnacgcgtggtggccatagtcttaggaggagctaatgaaggagcccgtacagccctctcggtggtgacctggaataaagacattgtcactaaaatcacccccgagggggccgaagagtggagtcttgccatcccagtcatgtgcctgctggcaaataccacgttcccctgctcccagcccccttgcactccctgctgctatgaaagggaaccagaggaaaccctacgcatgcttgaggacaacgtcatgagacctgggtactatcagctgctacaagcatnnnnnnnnnnnnntagccacgattggaccaagctgcgttacatggacaaccacacgccagcggacgcagagagggcggggctatttgtaagaacatcagcaccgtgcacgattactggaacaatgggacacttcatcctgacccgatgtccgaaaggggaaactctgacggtgggattcactgacngtaggaagatcagtcactcatgtacgcacccatttcaccatgaccctcctgtgataggccgggagaaattccattcccgaccgcagcacnnnnnngagctgccttgcagcacgtacgtgcagagcaccgccgcaactaccgaggagatagaggtacacatgcccccagacacccctgatcgcacattgatgtcacaacagtccggcaacgtaaagatcacagttaatggccagacggtgcggtacaagtgtaattgcggtggctcaaatgaaggactaataactacagacaaagtgattaataactgcaaagttgatcaatgtcatgccgcggtcaccaatcacaaaaagtggcagtacaactcccctctggtcccgcgtaatgctgaacttggggaccgaaaaggaaaaatccacatcccgtttccgctggcaaatgtaacatgcagggtgcctaaagcaaggaaccccaccgtgacgtacgggaaaaaccaagtcatcatgctactgtatcccgaccacccaacactcctgtcctaccggaacatgggagaagaaccaaactaccaagaagagtgggtgacacataagaaggaagtcgtgctaaccgtgccgactgaagggctcgaggtcacgtggggtaacaacgagccgtataagtattggccgcagttatctacaaacggtacagcccatggccacccgcatgagataattctgtattattatgagctgtaccctactatgactgtagtagttgtgtcagtggcctcgttcgtactcctgtcgatggtgggtgtggcngtggggatgtgcatgtgcgcacgacgcagatgcannnnnnnnnnngaactgacaccaggagctaccgtccctttcctgcttagcctaatatgctgcatcagaacagctaaagcggccacataccaagaggctgcggtatacctgtggaatgagcagcaacctttgttttggctacaagcccttattccgctggcagccctgatnnnnnnnnnnnnnnnnnnnnnnnnnnnnnnnnnnnnnnnnnnnnnnnnnnnnnnnnnnnnnnnnnnnnnnnnnnnnnnnnnnnnnntnagcgcgtacgaacacgtaacagtgatcccgaacacggtgggagtaccgtataagactctagtcaatagaccgggctacagtcccatggtattggagatggaactactgtcagtcactttggagccaacgctatcgcttgattacatcacgtgcgagtacaaaaccgttatcccgtctccgtacgtgaaatgctgcggtacagcagagtgcaaggacaaaaacctacctgactacagctgtaaggtcttcaccggcgtctacccatttatgtggggcggagcctnnnnnnntcattgtggggccaatgtcttcagcctggacacctttcgacaacaaaattgtggtgtacaaaggtgacgtctataacatggactacccgccctttggcgcaggaagaccaggacaatttggcgatgtccaaagtcgcacacctgagagtaaagacgtctatgctaatacacaactggtactgcagagaccggctgcgggtacggtacatgtgccatactctcaggcaccatctggctttaagtattggctaaaagaacgaggggcgtcgctgcagcacacagcaccatttggctgccaaatagcaacaaacccggtaagagcggtgaattgcgccgtagggaacatgcccatctccatcgacataccgga

>MK752957

cgtctgcccgatgcgcagcgcagaagatcccgagagactcgccaattatgcgagaaagctagcatctgctgcaggaaaagtcctggacagaaacatctctggaaagatcggggacttacaagcagtaatggccgtgccagacacggagacgccaacattctgcttgcacacagatgtctcatgtagacagagagcagacgtcgcgatataccaagacgtctatgctgtacacgcacccacgtcgctataccaccaggcgattaaaggggtccgagtggcgtactgggtagggttcgacacaaccccgttcatgtacaatgccatggcgggtgcctacccctcatattcgacaaactgggcagacgagcaggtactgaaggctaagaacataggattatgttcaacagacctgacggaaggtagacgaggtaaattgtctattatgagagggaagaagctaaaaccgtgtgaccgtgtgctgttctcagtagggtcaacgctctacccggaaagccgcaagctacttaagagctggcacttaccatcggtgttccatctaaagggcaaactcagcttcacatgccgctgtgatacagtggtttcgtgcgagggctacgtcgttaagagaataacgatgagcccaggcctttacggaaaaaccacagggtatgcggtaacccaccacgcagacggattcttgatgtgcaagactaccgacacggttgacggcgaaagagtgtcattctcggtgtgcacatacgtgccggcgaccatttgtgatcaaatgaccggcatccttgctacagaagtcacgccggaggatgcacagaagctgttggtggggctgaaccagagaatagtggttaacggcagaacgcaacggaatacgaacaccatgaaaaactatctgcttcccgtggtcgcccaagccttcagtaagtgggcaaaggagtgccggaaagacatggaagatgaaaaactcctgggggtcagagaaagaacactgacctgctgctgtctatgggcattcaagaagcagaaaacacacacggtctacaagaggcctgatacccagtcaattcagaaggttcaggccgagtttgacagctttgtggtaccgagcctgtggtcgtccgggttgtcaatccccttgaggactagaatcaaatggttgttaagcaaggtgccaaaaaccgacctgatcccatatagcggggacgcccaagaagcccgggacgcagaaaaagaagcagaggaagaacgagaagcannnnnnnnnnnnnnnnnnnnnnnnnnnnnnnnnnnnnnnnnnnnnnnnnnnnnnnnnnnnnnnnnnnnnnnnnnnnnnnnnnnnnnnnnnnnnnnnnnnnnnnnnnnnnnnnnnnnnnnnnnnnnnnnnnnnnnnnnnnnnnnnnnnnnnnnnnnnnnnnnnnnnnnnnnnnnnnnnnnnnnnnnnnnnnnnnnnnnnnnnnnnnnnnnnnnnnnnnnnnnnnnnnnnnnnnnnnnnnnnnnnnnnnnnnnnnnnnnnnnnnnnnnnnnnnnnnnnnnnnnnnnnnnnnnnnnnnnnnnnnnnnnnnnnnnnnnnnnnnnnnnnnnnnnnnnnnnnnnnnnnnnnnnnnnnnnnnnnnnnnnnnnnnnnnnnnnnnnnnnnnnnnnnnnnnnnnnnnnnnnnnnnnnnnnnnnnnnnnnnnnnnnnnnnnnnnnnnnnnnnnnnnnnnnnnnnnnnnnnnnnnnnnnnnnnnnnnnnnnnnnnnnnnnnnnnnnnnnnnnnnnnnnnnnnnnnnnnnnnnnnnnnnnnnnnnnnnnnnnnngactggtactggtgggcgacttgactaatccgccctaccacgaattcgcatatgaagggctaaaaatccgccctgcctgcccatacaaaattgcagtcataggagtcttcggagtaccaggatctggcaagtcagctattatcaagaacctagttaccaggcaagacctggtgactagcggaaagaaagaaaactgccaagaaatcaccaccgacgtgatgagacagagaggtctagagatatctgcacgtacggttgactcgctgctcttgaatggatgcaacaggccagtcgacgtgttgtacgtagacgaggcgtttgngtgcnnnnnnnnnnnnnnnnnnnnnnnnnnnnnnnnnnnnnnnnnnnnnnnnnnnnnnnnnnnnnnnnnnnnnnnnnnnnnnnnnnnnnnnnnnnnnnnnnnnnnnnnnnnnnnnnnnnnnnnnnnnnnnnnnnnnnnnnnnnnnnnnnnnnnnnnnnnnnnnnnnnnnnnnnnnnnnnnnnnnnnnnnnnnnnnnnnnnnnnnnnnnnnnnnnnnnnnnnnnnnnnnnnnnnnnnagtacaacaagccgattgtagtggacactacaggctcaacaaaacctgacgctggagatctcgtgttaacgtgcttcagagggtgggttaaacaactgcaaattgactatcgtggatacgaggtcatgacagcagccgcatcccaagggttaaccagaaaaggagtttatgcagttaggcaaaaagttaatgaaaacccgctctatgcatcaacgtcagagcacgtcaacgnnnnnnnnnnnnnnnnnnnnnnnnnnnnnnnnnnnnnnnnnnnnnnnnnnnnnnnnnnnnnnnnnnnnnnnnnnnnnnnnnnnnnnnnnnnnnnnnnnnnnnnnnnnnnnnnnnnnnnnnnnnnnnnnnnnnnnnnnnnnnnnnnnnnnnnnnnnnnnnnnnnnnnnnnnnnnnnnnnnnnnnnnnnnnnnnnnnnnnnnnnnnnnnnnnnnnnnnnnnnnnnnnnnnnnnnnnnnnnnnnnnnnnnnnatgataggcagtggtcccagataattcaagccttcaaagaagataaagcatactcacctgaagtagccctgaatgaaatatgcacgcgcatgtatggggtggatttagacagtgggctattttctaaaccattggtgtctgtgtattacgcggataaccactgggataataggcctggagggaagatgttcggattcaaccccgaggcagcatccattctagaaagaaagtatccattcacaaaagggaagtggaacatcaacaagnnnnnnnnnnnnnnnnnnnnnnnnnnnnnnnnnnnnnnnnnnnnnnnnnnnnnnnnnnnnnnnnnnnnnnnnnnnnnnnnnnnnnnnnnnnnnnnnnnnnnnnnnnnnnnnnnnnnnnnnnnnnnnnnnnnnnnnnnnnnnnnnnnnnnnnnnnnnnnnnnnnnnnnnnnnnnnnnnnnnnnnnnnnnnnnnnnnnnnnnnnnnnnnnnnnnnnnnnnnnnnnnnnnnnnnnnnnnnnnnnnnnnnnnnnnnnnnnnnnnnnnnnnnnnnnnnnnnnnnnnnnnnnnnnnnnnnnnnnnnnnnnnnnnnnnnnnnnnnnnnnnnnnnnnnnnnnnnnnnnnnnnnnnnnnnnnnnnnnnnnnnnnnnnnnnnnnnnnnnnnnnnnnnnnnnnnnnnnnnnnnnnnnnnnnnnnnnnnnnnnnnnnnnnnnnnnnnnnnnnnnntagaaccagtgaacgagtcatctgcgtgttgggacgcaagttcagatcgtctagagcgttgaaaccaccatgtgtcaccagcaacactgagatgtttttcctattcagcaactttgacaatggcagaaggaatttcacaactcatgtcatgaataatcaactgaatgcagcctttgtaggacaggccacccgagcaggatgtgcaccgtcgtaccgggtaaaacgcatggacatcgcgaagaacgatnnagngtgcnnnnnnnnnnnnnnnnnnnnnnnnnnnnnnnnnnnnnnnnnnnnnnnnnnnnnnnnnnnnnnnnnnnnnnnnnnnnnnnnnnnnnnnnnnnnnnnnnnnnnnnnnnnnnnnnnnnnnnnnnnnnnnnnnnnnnnnnnnnnnnnnnnnnnnnnnnnnnnnnnnnnnnnnnnnnnnnnnnnnnnnnnnnnnnnnnnnnnnnnnnnnnnnnnnnnnnnnnnnnnnnnnnnnnnnnnnnnnnnnnnnnnnnnnnnnnnnnnnnnnnnnnnnnnnnnnnnnnnnnnnnnnnnnnnnnnnnnnnnnnnnnnnnnnnnnnnnnnnnnnnnnnnnnnnnnnnnnnnnnnnnnnnnnnnnnnnnnnnnnnnnnnnnnnnnnnnnnnnnnnnnnnnnnnnnnnnnnnnnnnnnnnnnnnnnnnnnnnnnnnnnnnnnnnnnnnnnnnnnnnnnnnnnnnnnnnnnnnnnnnnnnnnnnnnnnnnnnnnnnnnnnnnnnnnnnnnnnnnnnnnnnnnnnnnnnnnnnnnnnnnnnnnnnnnnnnnnnnnnnnnnnnnnnnnnnnnnnnnnnnnnnnnnnnnnnggcagtggacatggcagagatatatactatgtggccaaagcaaacagaggccaacgagcaagtctgcctatatgccctnggggaaagtattgaatcgatcaggcagaaatgcccggtggatgatgcagatgcatcgtctcctccaaaaactgtcccgtgcctttgccgttacgccatgactccggaacgcgtcacccgacttcgcatgaaccacgtnnnnnnnnnnnnnnnnnnnnnnnnnnnnnnnnnnnnnnnnnnnnnnnnnnnnnnnnnnnnnnnnnnnnnnnnnnnnnnnnnnnnnnnnnnnnnnnnnnnnnnnnnnnnnnnnnnnnnnnnnnnnnnnnnnnnnnnnnnnnnnnnnnnnnnnnnnnnnnnnnnnnnnnnnnnnnnnnnnnnnnnnnnnnnnnnnnnnnnnnnnnnnnnnnnnnnnnnnnnnnnnnnnnnnnnnnnnnnnnnnnnnnnnnnnnnnnnnnnnnnnnnnnnnnnnnnnnnnnnnnnnnnnnnnnnnnnnnnnnnnnnnnnnnnnnnnnnnnnnnnnnnnnnnnnnnnnnnnnnnnnnnnnnnnnnnnnnnnnnnnnnnnnnnnnnnnnnnnnnnnnnnnnnnnnnnnnnnnnnnnnnnnnnnnnnnnnnnnnnnnnnnnnnnnnnnnnnnnnnnnnnnnnnnnnnnnnnnnnnnnnnnnnnnnnnnnnnnnnnnnnnnnnnnnnnnnnnnnnnnnnnnnnnnnnncaggcaccgccgagtaccgccacggaactgaatcatccgccgatctccttcggagcaccaagcgagacgttccccattacatttggggacttcaacgaaggagaaatcgaaagcttgtcttctgagctgctaactttcggagacttcctacccggagaagtggatgacttgacagatagcgactggtccacgtgctcagacacggacgacgagttactagacagggcaggtgggtatatattctcgtcggacaccggtccaggtcatttacaacagaagtcagtacgccagtcagtgctgccggtgaacaccctggaggaagtccacgaggagaagtgttacccacctaagttggatgaagtaaaggagcaactattacttaagaaactccaggagagtgcatccatggccaacagaagcaggtaccagtcgcgcaaagtagaaaacatgaaagcaacaatcatccagagactaaagagaggctgtagactatacttaatgtcagagacctcaaaagtccctacttaccggaccacatatccggcgcctgtgtactcgcctccgatcaacgtccgactgtccaaccccgagtccgcagcggcagcatgcaatgagttcctagctagaaactatccaactgtctcatcataccaaattaccgacgagtatgatgcatatttagacatggtggacgggtcggagagttgcctggatcgagcgacattcaatccgtcaaaacttaggagctacccgaaacagcacgcttaccatgcgccctccatcagaagcgctgtnnnnnnnnnnnnnnnnnnnnnnnnnnnnnnnnnnnnnnnnnnnnnnnnnnnnnnnnnnnnnnnnnnnnnnnnnnnnnnnnnnnnnnnnnnnnnnnnnnnnnnnnnnnnnnnnnnnnnnnnnnnnnnnnnnnnnnnnnnnnnnnnnnnnnnnnnnnnnnnnnnnnnnnnnnnnnnnnnnnnnnnnnnnnnnnnnnnnnnnnnnnnnnnnnnnnnnnnnnnnnnnnnnnnnnnnnnnnnnnnnnnnnnnnnnnnnnnnnnnnnnnnnnnnnnnctgcaggaagtaccaatggataggttcacagtagatatgaaaagggacgtgaaggtgactcctggtacaaagcacacagaggaaagacctaaggtgcaggttatacaggcggctgaacccttggcgacagcatacctatgtgggattcacagagagctggttaggaggctgaacgccgtcctcctacccaatgtacatacactatttgatatgtctgccgaggatttcgatgccatcatagccgcacactttaagccaggagacactgttttagaaacggacatagcctcctttgacaagagccaagatgattcacttgcgcttactgccttgatgttgttagaagatttaggggtggatcactccctgttggacttgatagaggccgcttttggagagatttccagctgtcannnnnnnnnnnnnnnnnnnnnnnnnnnnggcgccatgatgaaatctggtatgttcctaactctgttcgtcaacacattgctaaacatcaccatcgctagccgcgtgctggaagatcgtctgacaaaanccgcgtnngcggccttcatcggcgacgacaacataatacatggagtcgtctccgatgaattgatggcagccagatgcgccacttggatgaacatggaagtgaagatcatagatgcagttgtatccttgaaagncccttacntttgtgganggtttatactgcacgatactgtgacgggaacagcctgcagagtggcagacccgctaaaaaggctattcaaactgggcaaaccgctagcggcaggtgacgaacaagatgaagatagaagacgagcgctggctgacgaagtgatcagatggcaacgaacagggctaattgatgagttggagaaagcggtatactctaggtatgaagtgcagggtatatcagttgtggtaatgtccatggccacctttgcaagctccagatctaacttcgagaagctcagaggaccccagttaatnaactgacaatgcgcgcggtaccccaacagaagccacgcaggaatcggaagaataagaagcaaaagcaaaagcgacaggcgccacaaaacaacacaaaccnaaagaagcagccgcctnaaaagaaaccagctcnaaagnaaaagaagccgggccgcagagagaggatgtgcatgaaaatcgaaaatgattgtanttttgaagtcaagtatgaaggtaaggtaacaggttacgcgtgcttggtgggggacaaagtaatgaaaccagcnnnnnnnnnnnnnnnnnnnnnnnnnnnnnnnnnnnnnnnnnnnnnnnnnnnnnnnnnnnnnnnnnnnnnnnnnnnnnnnnnnnnnnnnnnnnnnnnnnnnnnnnnnnnnnnnnnnnnnnnnnnnnnnnnnnnnnnnnnnnnnnnnnnnnnnnnnnnnnnnnnnnnnnnnnnnnnnnnnnnnnnnnnnnnnnnnnnnnnnnnnnnnnnnnnnnnnnnnnnnnnnnnnnnnnnnnnnnnnnnnnnnnnnnnnnnacgcgtggtggccatagtcttaggaggagctaatgaaggagcccgtacagccctctcggtggtgacctggaataaagacattgtcactaaaatcacccccgagggggccgaagagtggagtcttgccatcccagtcatgtgcctgctggcaaataccacgttcccctgctcccagcccccttgcactccctgctgctatgaaagggaaccagaggaaaccctacgcatgcttgaggacaacgtcatgagacctgggtactatcagctgctacaagcatnnnnnnnnnnnnnnnnccacgattggaccaagctgcgttacatggacaaccacacgccagcggacgcagagagggcggggctatttgtaagaacatcagcaccgtgcacgattactggaacaatgggacacttcatcctgacccgatgtccgaaaggggaaactctgacggtgggattcactgacngtaggaagatcagtcactcatgtacgcacccatttcaccatgaccctcctgtgataggccgggagaaattccattcccgaccgcagcacnnnnnngagctgccttgcagcacgtacgtgcagagcaccgccgcaactaccgaggagatagaggtacacatgcccccagacacccctgatcgcacattgatgtcacaacagtccggcaacgtaaagatcacagttaatggccagacggtgcggtacaagtgtaattgcggtggctcaaatgaaggactaataactacagacaaagtgattaataactgcaaagttgatcaatgtcatgccgcggtcaccaatcacaaaaagtggcagtacaactcccctctggtcccgcgtaatgctgaacttggggaccgaaaaggaaaaatccacatcccgtttccgctggcaaatgtaacatgcagggtgcctaaagcaaggaaccccaccgtgacgtacgggaaaaaccaagtcatcatgctactgtatcccgaccacccaacactcctgtcctaccggaacatgggagaagaaccaaactaccaagaagagtgggtgacacataagaaggaagtcgtgctaaccgtgccgactgaagggctcgaggtcacgtggggtaacaacgagccgtataagtattggccgcagttatctacaaacggtacagcccatggccacccgcatgagataattctgtattattatgagctgtaccctactatgactgtagtagttgtgtcagtggcctcgttcgtactcctgtcgatggtgggtgtggcagtggggatgtgcatgtgcgcacgacgcagatgcannnnnnnnnnnnnnnnnnnnnnnnnnnnnnnnnnnnnnnnnnnnnnnnnnnnnnnnnnnnnnnnnnnnnnnnnnnnnnnnnnnnnnnnnnnnnnnnnnnnnnnnnnnnnnnnnnnnnnnnnnnnnnnnnnnnnnnnnnnnnnnnnnnnnnnnnnnnnnnnnnnnnnnnnnnnnnnnnnnnnnnnnnnnnnnnnnnnnnnnnnnnnnnnnnnnnnnnnnnnnnnnnnnnnnnnnnnnnnnnnnnnnnnnnnnnnnnnnagcgcgtacgaacacgtaacagtgatcccgaacacggtgggagtaccgtataagactctagtcaatagaccgggctacagtcccatggtattggagatggaactactgtcagtcactttggagccaacgctatcgcttgattacatcacgtgcgagtacaaaaccgttatcccgtctccgtacgtgaaatgctgcggtacagcagagtgcaaggacaaaaacctacctgactacagctgtaaggtcttcaccggcgtctacccatttatgtggggcggagcctnnnnnnnnnnnnnnnnngccaatgtcttcagcctggacacctttcgacaacaaaattgtggtgtacaaaggtgacgtctataacatggactacccgccctttggcgcaggaagaccaggacaatttggcgatatccaaagtcgcacacctgagagtaaagacgtctatgctaatacacaactggtactgcagagaccggctgcgggtacggtacatgtgccatactctcaggcaccatctggctttaagtattggctaaaagaacgaggggcgtcgctgcagcacacagcaccatttggctgccaaatagcaacaaacccggtaagagcggtgaattgcgccgtagggaacatgcccatctccatcgacataccgga

>MK752958

cgtctgcccgatgcgcagcgcagaagatcccgagagactcgccaattatgcgagaaagctagcatctgctgcaggaaaagtcctggacagaaacatctctggaaagatcggggacttacaagcagtaatggccgtgccagacacggagacgccaacattctgcttgcacacagatgtctcatgtagacagagagcagacgtcgcgatataccaagacgtctatgctgtacacgcacccacgtcgctataccaccaggcgattaaaggggtccgagtggcgtactgggtagggttcgacacaaccccgttcatgtacaatgccatggcgggtgcctacccctcatattcgacaaactgggcagacgagcaggtactgaaggctaagaacataggattatgttcaacagacctgacggaaggtagacgaggtaaattgtctattatgagagggaagaagctaaaaccgtgtgaccgtgtgctgttctcagtagggtcaacgctctacccggaaagccgcaagctacttaagagctggcacttaccatcggtgttccatctaaagggcaaactcagcttcacatgccgctgtgatacagtggtttcgtgcgagggctacgtcgttaagagaataacgatgagcccaggcctttatggaaaaaccacagggtatgcggtaacccaccacgcagacggattcttgatgtgcaagactaccgacacggttgacggcgaaagagtgtcattctcggtgtgcacatacgtgccggcgaccatttgtgatcaaatgaccggcatccttgctacagaagtcacgccggaggatgcacagaagctgttggtggggctgaaccagagaatagtggttaacggcagaacgcaacggaatacgaacaccatgaaaaactatctgcttcccgtggtcgcccaagccttcagtaagtgggcaaaggagtgccggaaagacatggaagatgaaaaactcctgggggtcagagaaagaacactgacctgctgctgtctatgggcattcaagaagcagaaaacacacacggtctacaagaggcctgatacccagtcaattcagaaggttcaggccgagtttgacagctttgtggtaccgagcctgtggtcgtccgggttgtcaatccccttgaggactagaatcaaatggttgttaagcaaggtgccaaaaaccgacctgatcccatatagcggggacgcccaagaagcccgggacgcagaaaaagaagcagaggaagaacgagaagcagaacngnnnnnnnnnnnnnnnnnnnnnnnnnnnnnnnnnnnnnnnnnnnntcaggtcgaaatcgacgtggaacagcttgaggacagagcgggtgcaggaataatagagactcctagaggagctatcaaagttactgcccaaccaacagaccacgtcgtgggagagtacctggtactttccccgcagaccgtactacggagccagaagctcagcctaattcacgctttggcggagcaagtgaagacgtgnnnncacagcggacgagcagggaggtacgcggtcgaagcgtacgacggccgagtcctagtgccctcaggctatgcaatctcgcctgaagacttccagagtctaagcgaaagcgcaacgatggtgtataacgaaagagagttcgtaaacagaaagctacaccacattgcgatgcacggaccagccctgaacaccgacgaagagtcgtatgagctggtgagggcagagaggacagaacacgagtacgtctacgatgtggaccagagaagatgctgtaagaaggaagaagctgcaggactggtactggtgggcgacttgactaatccgccctaccacgaattcgcatatgaagggctaaaaatccgccctgcctgcccatacaaaattgcagtcataggagtcttcggagtaccaggatctggcaagtcagctattatcaagaacctagttaccaggcaagacctggtgactagcggaaagaaagaaaactgccaagaaatcaccaccgacgtgatgagacagagaggtctagagatatctgcacgtacggttgactcgctgctcttgaatggatgcaacaggccagtcgacgtgttgtacgtagacgaggcgtttgcgtgcnnnnnnnnnnnnnnnnnnnnnnnnnnnnnnnnnnnnnnnnnnnnnnnnnnnnnnnnnnnnnnnnnnnnnnnnnnnnnnnnnnnnnnnnnnnnnnnnnnnnnnnnnnnnnnnnnnnnnnnnnnnnnnnnnnnnnnnnnnnnnnnnnnnnnnnnnnnnnnnnnnnnnnnnnnnnnnnnnnnnnnnnnnnnnnnnnnnnnnnnnnnnnnnnnnnnnnnnnnnnnnnnnnnnnnnnagtacaacaagccgattgtagtggacactacaggctcaacaaaacctgacgctggagatctcgtgttaacgtgcttcagagggtgggttaaacaactgcaaattgactatcgtggatacgaggtcatgacagcagccgcatcccaagggttaaccagaaaaggagtttatgcagttaggcaaaaagttaatgaaaacccgctctatgcatcaacgtcagagcacgtcaacgnnnncctaacacgtacggaaggtaaactggtatggaagacactttccggtgacccgtggataaagacgctgcagaacccaccgaaaggaaacttcaaagcaactatcaaggagtgggaggtggagcatgcatcaataatggcgggcatctgcagtcaccaaatgacctttgatacattccaaaacaaagccaatgtttgttgggctaagagcttggtccctatcctcgaaacagcggggataaaactaaatgataggcagtggtcccagataattcaagccttcaaagaagataaagcatactcacctgaagtagccctgaatgaaatatgcacgcgcatgtatggggtggatttagacagtgggctattttctaaaccattggtgtctgtgtattacgcggataaccactgggataataggcctggagggaagatgttcggattcaaccccgaggcagcatccattctagaaagaaagtatccattcacaaaagggaagtggaacatcaacaagnnnnnnnnnnnnnnnnnnnnnnnnntagaagactttaaccctaccaccaatattataccggccaataggagactaccacactcattagtggccgaacaccgcccagtaaaaggggaaagaatggaatggctggttaataagataaacggccaccacgtgctcctggtcagtggctataatcttgtactgcctactaagagagtcacttgggtagcgccactaggtgtccgcggagcggactacacatacaacctagagttgggtctgccagcaacgcttggcaggtatgacctagtggtcataaacatccacacaccttttcgcatacaccattaccaacagtgcgtagaccacgcaatgaaactgcaaatgctcgggggtgactcattgagactgctcaaaccgggtggctctctattgnnnnnnnnnnnnnnnnnnnnnnntagaaccagtgaacgagtcatctgcgtgttgggacgcaagttcagatcgtctagagcgttgaaaccaccatgtgtcaccagcaacactgagatgtttttcctattcagcaactttgacaatggcagaaggaatttcacaactcatgtcatgaataatcaactgaatgcagcctttgtaggacaggccacccgagcaggatgtgcaccgtcgtaccgggtaaaacgcatggacatcgcgaagaacgatnnagngtgcnnnnnnnnnnnnnnnnnnnnnnnnnnnnnnnnnnnnnnnnnnnnnnnnnnnnnnnnnnnnnnnnnnnnnnnnnnnnnnnnnnnnnnnnnnnnnnnnnnnnnnnnnnnnnnnnnnnnnnnnnnnnnnnnnnnnnnnnnnnnnnnnnnnnnnnnnnnnnnnnnnnnnnnnnnnnnnnnnnnnnnnnnnnnnnnnnnnnnnnnnnnnnnnnnnnnnnnnnnnnnnnnnnnnnnnnnnnnnnnnnnnnnnnnnnnnnnnnnnnnnnnnnnnnnnnnnnnnnnnnnnnnnnnnnnnnnnnnnnnnnnnnnnnnnnnnnnnnnnnnnnnnnnnnnnnnnnnnnnnnnnnnnnnnnnnnnnnnnnnnnnnnnnnnnnnnnnnnnnnnnnnnnnnnnnnnnnnnnnnnnnnnnnnnnnnnnnnnnnnnnnnnnnnnnnnnnnnnnnnnnnnnnnnnnnnnnnnnnnnnnnnnnnnnnnnnnnnnnnnnnnnnnnnnnnnnnnnnnnnnnnnnnnnnnnnnnnnnnnnnnnnnnnnnnnnnnnnnnnnnnnnnnnnnnnnnnnnnnnnnnnnnnnnnnnnnggcagtggacatggcagagatatatactatgtggccaaagcaaacagaggccaacgagcaagtctgcctatatgccctgggggaaagtattgaatcgatcaggcagaaatgcccggtggatgatgcagatgcatcgtctcctccaaaaactgtcccgtgcctttgccgttacgccatgactccggaacgcgtcacccgacttcgcatgaaccacgtcacaagtataattgtgtgttcctcgtttccccttccaaagtacaaaatagaaggagtgcaaaaagtcaaatgctctaaggtaatgctatttgaccacaacgtgccatcacgcgtaagtccaagggaatatagatcttccccggaggctgcacaggaagcgagtacaaccacgtcactgacgcatagccaattcgacctaagcgttgacggcgagatactacccgtcccgtcagatctggatgctgacgccccagccctagaaccagctctagacgacagggcgacacacacgttgccatccacaaccggaaaccttgcggccgtgtctgactgggtaatgagcaccgtacctgtcgcgccgcccagaagaaggcgagggagaaacctgactgtgacatgcgacgagagagaagggaacataacacccatggctagcgtccgattctttagggcagagctgcgtccggtcgtacaagaaacagcggagacgcgtgacacagcaatgtctcttcaggcaccgccgagtaccgccacggaactgaatcatccgccgatctccttcggagcaccaagcgagacgttccccattacatttggggacttcaacgaaggagaaatcgaaagcttgtcttctgagctgctaactttcggagacttcctacccggagaagtggatgacttgacagatagcgactggtccacgtgctcagacacggacgacgagttactagacagggcaggtgggtatatattctcgtcggacaccggtccaggtcatttacaacagaagtcagtacgccagtcagtgctgccggtgaacaccctggaggaagtccacgaggagaagtgttacccacctaagttggatgaagtaaaggagcaactattacttaagaaactccaggagagtgcatccatggccaacagaagcaggtaccagtcgcgcaaagtagaaaacatgaaagcaacaatcatccagagactaaagagaggctgtagactatacttaatgtcagagacctcaaaagtccctacttaccggaccacatatccggcgcctgtgtactcgcctccgatcaacgtccgactgtccaaccccgagtccgcagcggcggcatgcaatgagttcctagctagaaactatccaactgtctcatcataccaaattaccgacgagtatgatgcatatttagacatggtggacgggtcggagagttgcctggatcgagcgacattcaatccgtcaaaacttaggagctacccgaaacagcacgcttaccatgcgccctccatcagaagcgctgtaccgtccccattccagaacacactacagaatgtactggcagcagccacgaaaagaaactgcaacgtcacacagatgagggaattacccactttggactcagcagtattcaacgtggagtgttttaaaaaattcgcatgcaaccaagaatactgggaagaatttgctgccagccctattaggataacaactgagaatttaacaacctatgttactaaactaaaagggccaaaagcagcagcgctatttgcnnnnnnnnnnnnnnnnctgccactgcaggaagtaccaatggataggttcacagtagatatgaaaagggacgtgaaggtgactcctggtacaaagcacacagaggaaagacctaaggtgcaggttatacaggcggctgaacccttggcgacagcatacctatgtgggattcacagagagctggttaggaggctgaacgccgtcctcctacccaatgtacatacactatttgatatgtctgccgaggatttcgatgccatcatagccgcacactttaagccaggagacactgttttagaaacggacatagcctcctttgacaagagccaagatgattcacttgcgcttactgccttgatgttgttagaagatttaggggtggatcactccctgttggacttgatagaggccgcttttggagagatttccagctgtcannnnnnnnnnnnnnnnnnnnnnnnnnnnggcgccatgatgaaatctggtatgttcctaactctgttcgtcaacacattgctaaacatcaccatcgctagccgcgtgctggaagatcgtctgacaaaatccgcgtgcgcggccttcatcggcgacgacaacataatacatggagtcgtctccgatgaattgatggcagccagatgcgccacttggatgaacatggaagtgaagatcatagatgcagttgtatccttgaaagccccttacttttgtggagggtttatactgcacgatactgtgacgggaacagcctgcagagtggcagacccgctaaaaaggctattcaaactgggcaaaccgctagcggcaggtgacgaacaagatgaagatagaagacgagcgctggctgacgaagtgatcagatggcaacgaacagggctaattgatgagttggagaaagcggtatactctaggtatgaagtgcagggtatatcagttgtggtaatgtccatggccacctttgcaagctccagatctaacttcgagaagctcagaggaccccagttaataaactgacaatgcgcgcggtaccccaacagaagccacgcaggaatcggaagaataagaagcaaaagcaaaagcgacaggcgccacaaaacaacacaaaccaaaagaagcagccgcctaaaaagaaaccagctcaaaagaaaaagaagccgggccgcagagagaggatgtgcatgaaaatcgaaaatgattgtatttttgaagtcaagtatgaaggtaaggtaacaggttacgcgtgcttggtgggggacaaagtaatgaaaccagcnnnnnnnnnnnnnnnnnnnnnnnnnnnnnnnnnnnnnnnnnnnnnnnnnnnnnnnnnnnnnnnnnnnnnnnnnnnnnnnnnnnnnnnnnnnnnnnnnnnnnnnnnnnnnnnnnnnnnnnnnnnnnnnnnnnnnnnnnnnnnnnnnnnnnnnnnnnnnnnnnnnnnnnnnnnnnnnnnnnnnnnnnnnnnnnnnnnnnnnnnnnnnnnnnnnnnnnnnnnnnnnnnnnnnnnnnnnnnnnnnnnnnnnnnnnnacgcgtggtggccatagtcttaggaggagctaatgaaggagcccgtacagccctctcggtggtgacctggaataaagacattgtcactaaaatcacccccgagggggccgaagagtggagtcttgccatcccagtcatgtgcctgctggcaaataccacgttcccctgctcccagcccccttgcactccctgctgctatgaaagggaaccagaggaaaccctacgcatgcttgaggacaacgtcatgagacctgggtactatcagctgctacaagcatnnnnnnnnnnnnnnngccacgattggaccaagctgcgttacatggacaaccacacgccagcggacgcagagagggcggggctatttgtaagaacatcagcaccgtgcacgattactggaacaatgggacacttcatcctgacccgatgtccgaaaggggaaactctgacggtgggattcactgacagtaggaagatcagtcactcatgtacgcacccatttcaccatgaccctcctgtgataggccgggagaaattccattcccgaccgcagcacnnnnnngagctgccttgcagcacgtacgtgcagagcaccgccgcaactaccgaggagatagaggtacacatgcccccagacacccctgatcgcacattgatgtcacaacagtccggcaacgtaaagatcacagttaatggccagacggtgcggtacaagtgtaattgcggtggctcaaatgaaggactaataactacagacaaagtgattaataactgcaaagttgatcaatgtcatgccgcggtcaccaatcacaaaaagtggcagtacaactcccctctggtcccgcgtaatgctgaacttggggaccgaaaaggaaaaatccacatcccgtttccgctggcaaatgtaacatgcagggtgcctaaagcaaggaaccccaccgtgacgtacgggaaaaaccaagtcatcatgctactgtatcccgaccacccaacactcctgtcctaccggaacatgggagaagaaccaaactaccaagaagagtgggtgacacataagaaggaagtcgtgctaaccgtgccgactgaagggctcgaggtcacgtggggtaacaacgagccgtataagtattggccgcagttatctacaaacggtacagcccatggccacccgcatgagataattctgtattattatgagctgtaccctactatgactgtagtagttgtgtcagtggcctcgttcgtactcctgtcgatggtgggtgtggcagtggggatgtgcatgtgcgcacgacgcagatgcannnnnnnnnnnnnnnnnnnaccaggngctaccgtccctttcctgcttagcntaatatgctgcatcagaacagctaaagcggccacataccaagaggctgcggtatacctgtggaatgagcagnnnnnnnnnnnnnnnnnnnnnnnnnnnnnnnnnnnnnnnnnnnnnnnnnnnnnnnnnnnnnnnnnnnnnnnnnnnnnnnnnnnnnnnnnnnnnnnnnnnnnnnnnnnnnnnnnnnnnnnnnnnnnnnnnnnnnnnagcgcgtacgaacacgtaacagtgatcccgaacacggtgggagtaccgtataagactctagtcaatagaccgggctacagtcccatggtattggagatggaactactgtcagtcactttggagccaacgctatcgcttgattacatcacgtgcgagtacaaaaccgttatcccgtctccgtacgtgaaatgctgcggtacagcagagtgcaaggacaaaaacctacctgactacagctgtaaggtcttcaccggcgtctacccatttatgtggggcggagcctnnnnnnnnnnnnnnnnnnccaatgtcttcagcctggacacctttcgacaacaaaattgtggtgtacaaaggtgacgtctataacatggactacccgccctttggcgcaggaagaccaggacaatttggcgatatccaaagtcgcacacctgagagtaaagacgtctatgctaatacacaactggtactgcagagaccggctgcgggtacggtacatgtgccatactctcaggcaccatctggctttaagtattggctaaaagaacgaggggcgtcgctgcagcacacagcaccatttggctgccaaatagcaacaaacccggtaagagcggtgaattgcgccgtagggaacatgcccatctccatcgacataccgga
