# Supplementary material for: Return of the founder Chikungunya virus to its place of introduction into Brazil is revealed by genomic characterization of exanthematic disease cases
Source: Emerg Microbes Infect. 2019 Dec 27;9(1):53–7. doi: 10.1080/22221751.2019.1701954 (PMC6968431; doi:10.1080/22221751.2019.1701954)
Supplement: Supplemental Material [file TEMI_A_1701954_SM8910.zip › Supplementary_Table_1_final.doc]

**Supplementary Table 1. Epidemiological data and genome covegare for the sequenced samples.**

| **ID** | **Sample** | **Collection date** | **Date onset symptoms** | **Sex** | **Age** | **Municipality** | **State** | **Acession Number** | **Reads** | **Coverage (%)** | **Ct** |
| --- | --- | --- | --- | --- | --- | --- | --- | --- | --- | --- | --- |
| LB2 | Serum | 02/08/2018 | 30/07/2018 | F | 1 | FEIRA DE SANTANA | BA | MK156060 | 218700 | 96,7554 | 25 |
| LB3 | Serum | 24/08/2018 | 22/08/2018 | M | 11 | CONCEICAO DO JACUIPE | BA | MK156053 | 177220 | 65,806 | 26,3 |
| LB7 | Serum | 24/08/2018 | 23/08/2018 | F | 26 | CORACAO DE MARIA | BA | MK156054 | 326013 | 78,6658 | 20,2 |
| LB9 | Serum | 21/08/2018 | 20/08/2018 | M | 3 | CORACAO DE MARIA | BA | MK156055 | 73010 | 71,529 | 21 |
| LB10 | Serum | 21/08/2018 | 19/08/2018 | M | 53 | CORACAO DE MARIA | BA | MK156056 | 333495 | 63,2763 | 23,4 |
| LB12 | Serum | 29/08/2018 | 30/08/2018 | F | 46 | CAMACARI | BA | MK156061 | 105713 | 80,6299 | 27,9 |
| LB13 | Serum | 20/08/2018 | 17/08/2018 | F | 30 | CAMACARI | BA | MK156062 | 170841 | 83,6353 | 27 |
| LB15 | Serum | 30/07/2018 | 28/07/2018 | F | 23 | CAMACARI | BA | MK156058 | 224376 | 80,5113 | 21,5 |
| LB17 | Serum | 27/07/2018 | 26/07/2018 | F | 23 | SALVADOR | BA | MK156059 | 256056 | 69,8527 | 23,9 |
| LB19 | Serum | 03/09/2018 | 31/08/2018 | F | 21 | CORACAO DE MARIA | BA | MK156063 | 231622 | 86,3867 | 27,9 |
| LB20 | Serum | 03/09/2018 | 02/09/2018 | F | 67 | CORACAO DE MARIA | BA | MK156064 | 222707 | 75,7027 | 24,1 |
| LB21 | Serum | 23/08/2017 | 20/08/2017 | F | 10 | IPECAETÁ | BA | MK752950 | 20392 | 67,0014 | 24,25 |
| LB22 | Serum | 27/07/2017 | 23/07/2017 | M | 59 | ITAMARAJU | BA | MK752951 | 23963 | 64,2398 | 23,17 |
| LB23 | Serum | 22/06/2017 | 18/06/2017 | M | 17 | ITAMARAJU | BA | MK752952 | 211707 | 69,8121 | 22,49 |
| LB24 | Serum | 16/08/2017 | 14/08/2017 | F | 23 | IPECAETÁ | BA | MK752953 | 23626 | 65,3065 | 20,45 |
| LB25 | Serum | 30/08/2017 | 29/08/2017 | F | 14 | IPECAETÁ | BA | MK752954 | 17616 | 69,2956 | 19,63 |
| LB26 | Serum | 05/06/2017 | 03/06/2017 | M | 22 | COARACI | BA | MK752955 | 27192 | 63,1476 | 22,38 |
| LB27 | Serum | 30/08/2017 | 26/08/2017 | F | 13 | IPECAETÁ | BA | MK752956 | 32152 | 67,7955 | 27,12 |
| LB28 | Serum | 23/08/2017 | 22/08/2017 | F | 62 | IPECAETÁ | BA | MK752957 | 234707 | 64,9695 | 16,25 |
| LB29 | Serum | 23/08/2017 | 14/08/2017 | F | 56 | IPECAETÁ | BA | MK752958 | 20030 | 72,02 | 17,73 |
